# Peptide‐Induced Ferroelectricity in Charge‐Transfer Supramolecular Materials

**DOI:** 10.1002/adma.202514940

**Published:** 2026-01-10

**Authors:** James V. Passarelli, Yang Yang, Cara S. Smith, Jing Hao, Dhwanit R. Dave, Ashwin Narayanan, Zaida Álvarez, Broderick K. Johnson, Kelly A. Marshall, Ivan Fithian, Hiroaki Sai, Ruomeng Qiu, Charlotte L. Stern, Liam C. Palmer, Evangelos Kiskinis, Samuel I. Stupp

**Affiliations:** ^1^ Department of Chemistry Northwestern University Evanston Illinois USA; ^2^ Center For Regenerative Nanomedicine Northwestern University Chicago Illinois USA; ^3^ Division of Electromagnetic Engineering KTH Royal Institute of Technology Stockholm Sweden; ^4^ Department of Materials Science and Engineering Northwestern University Evanston Illinois USA; ^5^ Department of Biomedical Engineering Northwestern University Evanston Illinois USA; ^6^ CIBER en Bioingeniería, Biomateriales y Nanomedicina CIBER‐BBN Madrid Spain; ^7^ The Ken & Ruth Davee Department of Neurology Feinberg School of Medicine Northwestern University Chicago Illinois USA; ^8^ Northwestern University Chicago Illinois USA

**Keywords:** charge transfer, neuronal axon growth, peptide symmetry breaking, self‐assembling peptides, supramolecular ferroelectrics

## Abstract

Organic ferroelectrics are of great interest in sustainable energy conversion, information storage, flexible electronics, and potential biomedical applications as soft implants, among many other applications. Despite their broad potential, the development of organic ferroelectrics has remained limited, with only a few known examples in solid‐state systems, primarily due to the lack of well‐established design strategies compared to inorganic systems. Bio‐inspired supramolecular chemistry offers a path to create functional nanostructures that are water‐processable and biocompatible. We report here on supramolecular charge transfer (CT) systems in which peptides are covalently linked to dyads of electron‐donating and electron‐accepting moieties, creating amphiphiles that self‐assemble into nanoscale ribbons in water. The peptide chirality‐induced symmetry breaking in these crystalline nanostructures not only results in second harmonic activity but also generates ferroelectric behavior across multiple CT systems, demonstrating a versatile supramolecular approach to the design of new organic ferroelectrics. Furthermore, culturing primary neuron cells on coatings of the ferroelectric materials promoted axonal growth and enhanced action potentials, indicating improved neuronal maturity facilitated by the polar structure of the ferroelectric nanomaterials. The supramolecular strategy used here holds promise to create new water‐processable ferroelectric biomaterials, opening avenues for innovative applications in cell charge transfer, neuronal axon growth, peptide symmetry breaking, self‐assembling peptides, supramolecular ferroelectrics, proliferation, and bioelectronics.

## Graphical abstract

Bio‐inspired supramolecular charge‐transfer amphiphiles self‐assemble into nanoribbons in water, where peptide chirality‐induced symmetry breaking generates robust ferroelectricity across multiple systems. These water‐processable organic ferroelectrics also enhance neuronal outgrowth and electrophysiological maturity, offering a versatile strategy for designing functional ferroelectric biomaterials for sustainable electronics and biointerfaces.

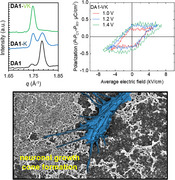

## 1 Introduction

Ferroelectric materials possess a polar structure that can be switched into bistable states by anti‐parallel external electric fields. Ferroelectricity has been an important topic in condensed matter science and continually yields advancements in applications including memory [1, 2], electro‐optical devices [3], and wearable electronics [4]. Organic ferroelectrics, given their light weight, low toxicity, and facile processing [5, 6, 7, 8, 9], present compelling alternatives to their inorganic counterparts, especially in biomedical applications such as biosensing, tissue engineering, among others [10, 11]. Utilizing supramolecular strategies, room‐temperature ferroelectric cocrystals composed of alternating stacks of electron donors and acceptors have been previously developed [12, 13, 14]. This chemical design disrupts the center of symmetry in these charge transfer (CT) cocrystals, leading to emergent physical properties such as second‐harmonic generation (SHG) and ferroelectricity [15]. Furthermore, recent achievements have also been reported in the formation of ferroelectric liquid crystals [16, 17], in which macroscopic polarization emerges through the alignment of the peptide‐based electroactive crystals and symmetry‐breaking intermolecular interactions such as hydrogen bonding [18, 19, 20, 21]. However, the use of these materials in biomedical applications and flexible electronics requires confinement of ferroelectricity to nanoscale structures in order to reduce their operating voltages [16].

We developed recently oligomeric vinylidene fluoride peptide amphiphiles (OVDF‐PAs) that self‐assemble into ultra‐low‐field ferroelectric nanostructures with lengths reaching several microns long and widths of hundreds of nanometers, typically with a thickness less than a molecular double layer [22]. In this system, we have hypothesized that ferroelectric phases containing OVDF nanocrystals are thermodynamically stable because lattice constants match with characteristic intermolecular distances in the β‐sheet secondary structures of the self‐assembling peptides. Molecular architectures based on complementary donor‐acceptor interactions can drive ordering at the scale of intermolecular interactions, impacting supramolecular co‐assembly and sorting [23, 24, 25]. Peptide materials with naphthalene diimide (NDI) and other electron donor or acceptor chromophores as hydrophobic groups have been reported to assemble into conductive supramolecular nanowires [26, 27]. Other interesting systems based on minimalist peptides have been developed that exhibit electro‐active properties such as piezoelectricity and conductivity for bioelectronics applications [28, 29, 30]. When chromophores are present in the chemical structure, the peptide unit can induce chirality within any complexes they may form [26, 31], suggesting new methods to develop symmetry‐breaking features and thus potential ferroelectricity in charge transfer systems.

We report here on supramolecular materials composed of peptide amphiphiles in which the donor and acceptor moieties are covalently linked. These molecules were designed to self‐assemble into ordered nanostructures via intermolecular CT interactions between the complementary components. We conjugated the donor‐acceptor moieties to minimalist peptides with one, two or four amino acids to create chiral secondary structures (such as *β*‐sheets) to break symmetry within the donor‐acceptor lattice, thus potentially enabling properties characteristic of noncentrosymmetric structures, including second harmonic generation and ferroelectricity. Finally, we assessed the biocompatibility of these biomolecular donor‐acceptor PA (DA‐PAs) materials by exposing them to primary mouse neurons.

## 2 Molecular Design and Self‐Assembly

**FIGURE 1:**
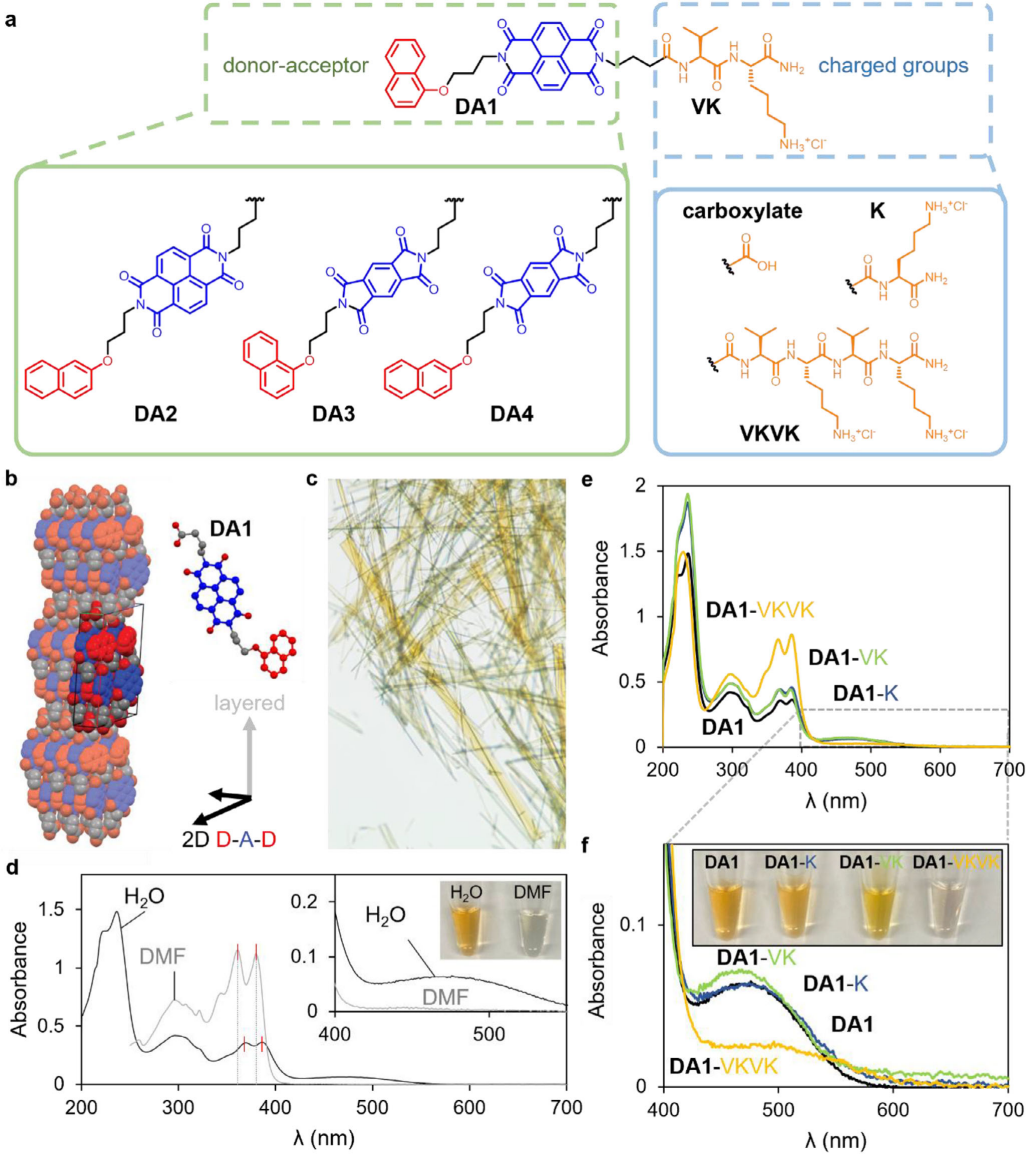
Molecular structures and self‐assembly of the donor‐acceptor peptide amphiphiles. a, Molecular structure of the DA‐PAs which include a naphthalene donor substituted with a three‐carbon alkoxy linker to the PMDI or NDI acceptor. The acceptor unit is connected by a three‐carbon linker to one of four charged groups: carboxylate, amide‐terminated lysine (K), amide‐terminated valine‐lysine (VK), or the amide‐terminated tetrapeptide VKVK. b, Crystal structure of single crystals of **DA1** obtained by slow evaporation of DMSO (the acceptor moiety is shown in blue, and the donor moiety is shown in red, and the crystal forms a layered structure with a head‐to‐tail arrangement of DA molecules within the layer and donor‐acceptor stacks). c, Photograph of crystals for **DA2** (see Figure S4 for **DA1** crystal indexing). d, UV–vis absorption spectra of **DA1** dissolved in DMF (black trace) or in water with 1 equivalent NaOH (grey trace) (the inset shows the charge transfer region of the UV–vis absorption spectra and a photograph of the 1 mM aqueous and DMF solution). e, f, UV–vis absorption spectra of aqueous solutions of **DA1** with 1 equivalent NaOH, **DA1‐K**, **DA1‐VK**, and **DA1‐VKVK** (e) magnified spectra in e in the charge transfer region (f) (the inset shows a photograph of the 1 mM aqueous solutions).

The molecular design targeted long‐range crystalline order in supramolecular assemblies containing electron donor and electron acceptor contacts using a covalent linkage between these two moieties. Naphthalene substituted with alkoxy groups in the 1 or 2 position was used as the donor and the acceptor units were either pyromellitic diimide (PMDI) or naphthalene diimide (NDI)) with substituents for aqueous solubility (Figure 1a). Incorporating donor and acceptor moieties into a single molecule could facilitate the assembly pathway of donor‐acceptor pairs with the long‐range crystallinity normally desired for electronic functions. We first synthesized carboxylic acid dyads with four permutations of the donor and acceptor moieties **DA1**–**DA4** (Figure S1). The pyromellitic variants yielded yellow solids, and the naphthalene diimides were orange in the solid state (Figure S1). We were able to obtain single crystals of **DA1**, **DA2**, and **DA3** suitable for X‐ray crystallography (see Figure 1b for the structure of **DA1**, and those of **DA2** and **DA3** are shown in Figures S2 and S3, respectively). The crystal structures revealed that molecules adopt an anti‐parallel packing such that the donor moiety of one molecule is next to the acceptor unit of the adjacent molecule, thus forming alternating donor‐acceptor p‐stacks along the crystallographic *b*‐axis. A two‐dimensional layer is defined by the *b*‐axis and the edge‐to‐edge stacking of the chromophores along the crystallographic *c*‐axis, whereas the third dimension (*a*‐axis) of this crystal is formed through the layering of these two‐dimensional chromophore sheets. Indexing of the faces of the **DA1** crystal shows that the largest dimension of the crystal corresponds to the π‐stacking axis, while the shortest dimension is parallel to the layering axis (Figure S4).

The **DA1**, **DA2**, and **DA4** amphiphiles are soluble in water at concentrations ranging from 1 to 5 mM with the addition of 1 equivalent of NaOH. The samples were then annealed at 80°C followed by a slow cooling step in an attempt to equilibrate the supramolecular structures formed, and optical absorption spectra of **DA1** in water and in *N*,*N*‐dimethylformamide (DMF) are shown in Figure 1d (see Figure S5 for the corresponding spectra of **DA2** and **DA4**). The vibronic absorption bands at 360 and 380 nm are attributed to the acceptor component of the **DA1** molecule (Figure S6). Compared to **DA1** in DMF, a bathochromic shift of these absorption bands, along with a general broadening of the UV absorption bands, is observed in water. Additionally, a broad absorption band emerges in the visible range between 400 and 550 nm in water, which is not observed in DMF. This broad absorption gives rise to the apparent orange color in solution and is attributed to charge transfer in the assembled state, whereas the DMF solution is colorless. This inference is supported by the detection of a strong charge transfer interaction in aqueous solutions and its suppression when samples are dissolved in DMF. This result suggests that charge transfer interactions are predominantly intermolecular, and that the short four‐atom linker between the donor and acceptor does not provide sufficient conformational flexibility for intramolecular charge transfer.

We then synthesized DA‐PAs with mono‐, di‐, or tetra‐peptide units coupled to the N‐terminus of **DA1**, **DA2**, **DA3**, and **DA4** using solid‐phase peptide synthesis (see Supporting Information for synthetic procedures and molecular characterization). Optical absorption spectroscopy performed on 1 mM annealed aqueous samples of DA1‐lysine·HCl (**DA1‐K**), DA1‐valine‐lysine·HCl (**DA1‐VK**), and DA1‐valine‐lysine‐valine‐lysine·2HCl (**DA1‐VKVK**) exhibits comparable intensity and position of the CT band relative to that observed in **DA1** (**Figure** **1** **e**,**f**). However, the tetrapeptide **DA1‐VKVK** shows a considerably weaker CT band as well as higher relative absorption of the 360 and 380 nm bands compared to the absorption bands centered at 300 nm. This absorption profile resembles the **DA1** monomer absorption in DMF (Figure 1d), indicating a reduced charge transfer band and weaker donor‐acceptor complexation, as further evidenced by the markedly lighter color of the solution (Figure 1f). Optical absorption spectra of **DA2**, **DA2‐K**, **DA2‐VK**, **DA3**, **DA3‐K**. **DA3‐VK**, **DA4**, **DA4‐K**, and **DA4‐VK** (Figures S7–S9) show similar CT bands to those observed in **DA‐1** containing molecules, suggesting the introduction of a single lysine or the valine‐lysine dipeptide does not suppress the CT interaction in crystallized assemblies of the molecules.

## 3 Structural Characterization of Donor–Acceptor Peptide Amphiphiles

**FIGURE 2:**
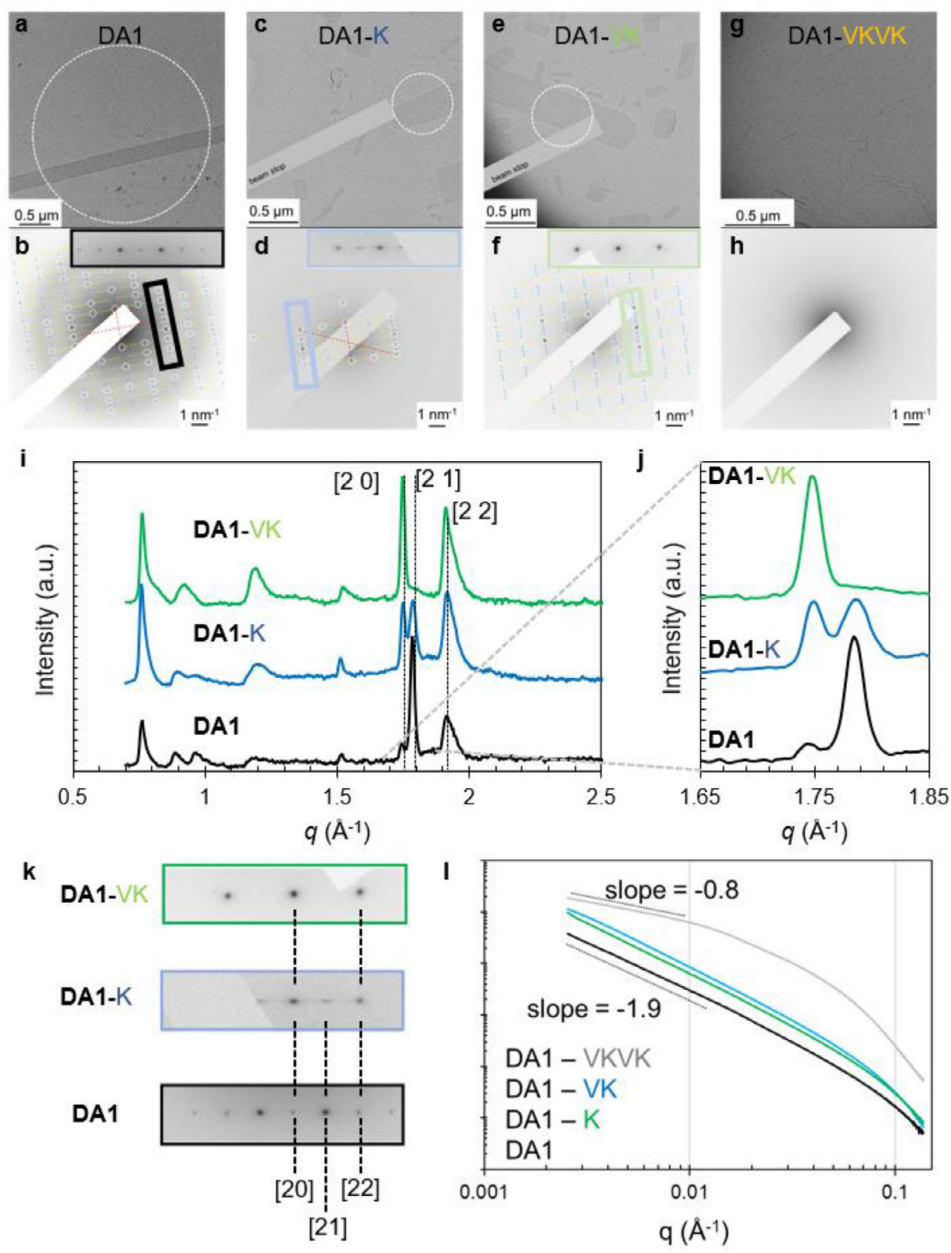
Structural characterization of donor‐acceptor peptide amphiphiles. a‐h, Transmission electron micrographs of **DA1** (a), **DA1‐K** (c), **DA1‐VK** (e), and **DA1‐VKVK** (g). Selected area electron diffraction (SAED) of **DA1** (b), **DA1‐K** (d), **DA1‐VK** (f), and **DA1‐VKVK** (h) (white dashed lines indicate the apertures of the selected areas and insets of b, d and f are enlarged from the corresponding colored regions in the diffractograms, the cross points of red dashed lines indicate the positions of the transmitted beam). i, j, Wide‐angle X‐ray scattering (WAXS) patterns for aqueous solutions of **DA1** with 1 equivalent NaOH, **DA1‐K,** and **DA1‐VK**. Enlargement of the relevant WAXS region for comparison to electron diffraction (j). k, SAED regions enlarged from the corresponding colored boxes in b, d, and f. l, Small‐angle X‐ray scattering (SAXS) patterns for aqueous solutions of DA1‐PA assemblies including **DA1** (black trace), **DA1‐K** (green trace), **DA1‐VK** (blue trace), and **DA1‐VKVK** (grey trace).

Transmission electron microscopy (TEM) was utilized to explore the effect of peptide units on supramolecular nanostructures and their internal crystalline structure. **DA1**, which does not contain any amino acids, forms ribbon‐shaped assemblies exceeding 5 µm in length and approximately 200 nm in width (Figure 2a). On the other hand, **DA1‐K** and **DA1‐VK** show more polydisperse nanostructures varying in dimensions, some maintaining the ribbon shape observed in **DA1** (short ribbons) as well as small platelets (see Figure 2c,e, respectively, and quantitative results in Figure S10). Selected area electron diffraction (SAED) of **DA1** (Figure 2b) reveals crystalline order and a rectangular unit cell with intermolecular spacings of *a* = 7 Å and *b* = 16 Å. The 7 Å spacing corresponds to the π‐stacking direction consisting of the two‐π‐stack pitch required by the antiparallel stacking along the long axis of the ribbon. The 16 Å axis is parallel to the width of the ribbons and likely represents the side‐to‐side spacing of the **DA1** chromophores, similar to that observed in the single‐crystal structure. SAED of **DA1‐K** (Figure 2d) reveals a similar unit cell to **DA1** with the same peak positions, but some smearing of the reflections is observed along the short axis of the ribbon, suggesting internal lateral disorder of the **DA1‐K** assemblies. The morphology of the nanostructures formed by **DA1‐VK** also appears similar to that of **DA1‐K** assemblies. The internal structure revealed by SAED (Figure 2f) shows reflections at the expected position for [2 0] and [2 2] planes based on the parent crystal lattices of **DA1** and **DA1‐K**. However, the [2 1] reflections that appeared smeared in **DA1‐K** are no longer observed in the **DA1‐VK** samples, revealing instead a new 2D rectangular lattice of *a* = 7 Å and *b* = 8 Å, which correspond to the long and short axes of the ribbons, respectively. The introduction of the dipeptide thus changed the unit cell, such that it consists of only one molecular repeat perpendicular to the π‐stacking axis as opposed to two in the **DA1** unit cell (see proposed stacking geometries in Figure S11). In great contrast to **DA1**, **DA1‐K**, and **DA1‐VK**, the tetrapeptide (**DA1‐VKVK)** does not form ribbons and instead organizes predominantly into thin filamentous nanostructures with no apparent crystallinity.

Since the SAED focuses on individual nanostructures in the dried state of the samples, we also carried out measurements of solution X‐ray scattering to better understand the structures of these assemblies in the liquid state (Figure 2i). Indexing the wide‐angle X‐ray scattering (WAXS) pattern of aqueous **DA1** samples reveals the same diffraction pattern observed in SAED (Figure S12). The [2 0], [2 1] and [2 2] reflections in WAXS show the same trends observed by SAED (Figure 2j,k), where there is a diminished relative intensity of the [2 1] reflection in samples of **DA1‐K** and the disappearance of this reflection in **DA1‐VK**. WAXS of **DA1‐VKVK** samples does not show any of the Bragg peaks observed in **DA1** but we did observe maxima consistent with *β*‐sheet spacing (Figure S13). Small‐angle X‐ray scattering (SAXS) scans of **DA1**, **DA1‐K** and **DA1‐VK** samples reveal slopes of approximately ‐2 in the low‐*q* region (Figure 2l), indicating the formation of ribbon‐shaped nanostructures. However, SAXS of **DA1‐VKVK** shows a slope of about ‐1 in the low‐*q* region, indicating the formation of one‐dimensional filament‐like nanostructures (Figure 2l). TEM, SAED, and transmission X‐ray scattering characterization of the other DA molecules and DA‐peptides, namely **DA2**, **DA2‐K**, **DA2‐VK**, **DA3**, and **DA3‐K**. **DA3‐VK**, **DA4**, **DA4‐K**, and **DA4‐VK** are provided in Figures S14–S16. These results reveal that both in the dried state and in solution, the headgroups of **DA1‐VK** and the other DA‐PAs form ordered nanostructures.

## 4 Symmetry Breaking in the DA‐PA nanostructures

**FIGURE 3:**
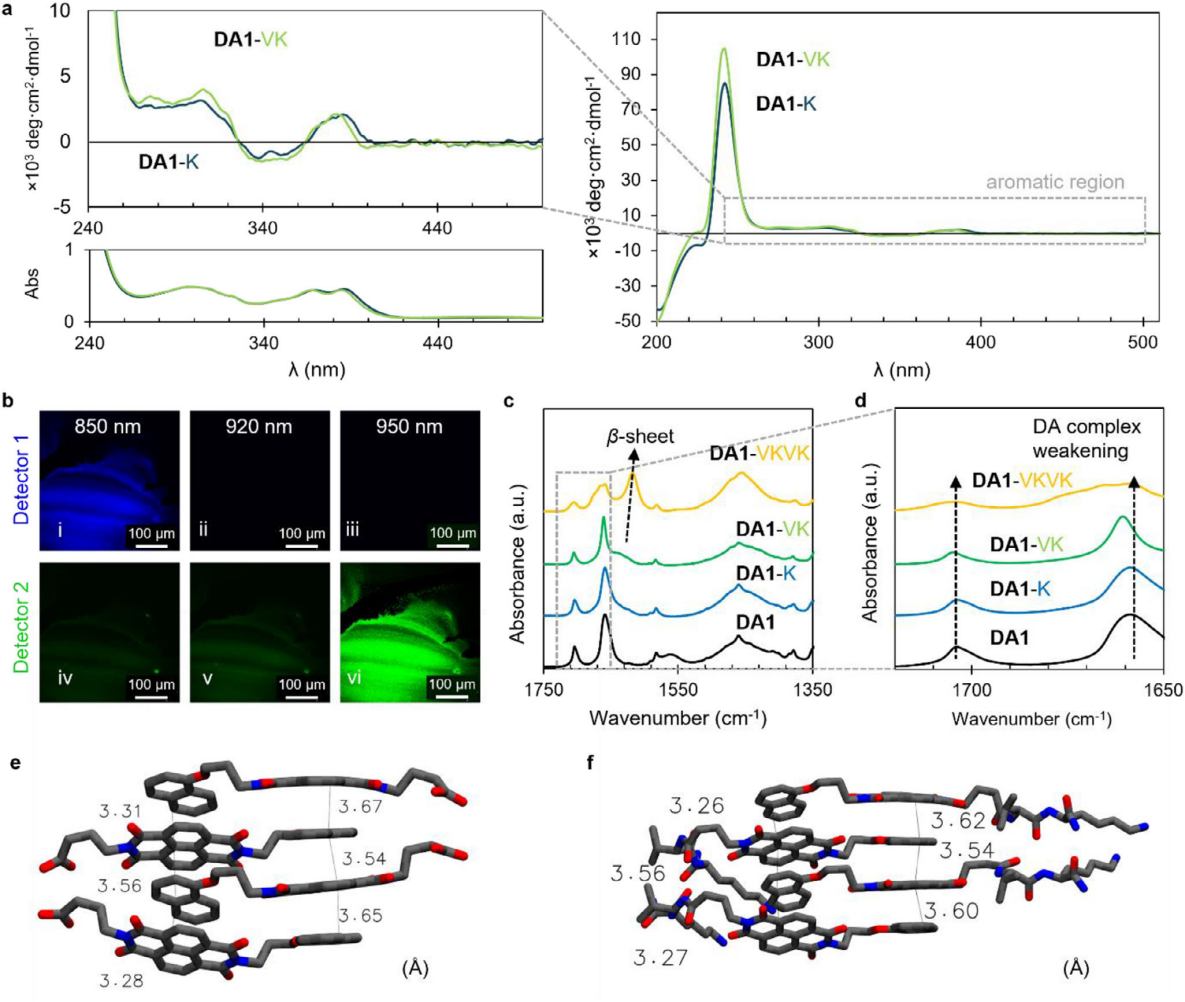
Symmetry breaking in the donor‐acceptor peptide amphiphiles crystal lattice. a, Circular dichroism spectroscopy of **DA1‐K** and **DA1‐VK** aqueous solutions enlarged in the aromatic region (top). An enlargement of UV–vis absorption data from Figure 1e is added for comparison (bottom). b, Two‐photon confocal microscopy of dried samples of **DA1‐VK**. Panels i‐iii) correspond to detector 1 with 380–450 nm detection window, iv‐vi) correspond to detector 2 with 470–550 nm detection window, i) and iv) correspond to excitation at 850 nm, which can only be imaged by detector 1, ii) and v) correspond to excitation at 920 nm, which cannot be imaged by these two detectors, and iii) and vi) correspond to excitation at 950 nm, which can only be imaged by detector 2. c, d, Infrared absorption spectroscopy for aqueous solutions of **DA1** with 1 equivalent NaOH (black trace), **DA1‐K** (blue trace), **DA1‐VK** (green trace), and **DA1‐VKVK** (orange trace). An enlargement of the imide region illustrates weakening of charge transfer interactions in **DA1‐VKVK** (d). e, f, DFT optimized structures showing π–π stacking distances (in Å) within a cluster of four molecules in **DA1**(e) and **DA1‐VK** (f).

Since ferroelectric behavior is only possible if the crystal structure lacks an inversion center, we investigated symmetry breaking within the CT complexes. Circular dichroism (CD) spectra of **DA1** did not reveal any signal in the aromatic region (Figure S17), which does not rule out the existence of chiral crystalline order but does indicate that there is not preferred handedness. In contrast, the chiral nature of the amino acid components gives rise to CD signals for solutions of **DA1‐K** and **DA1‐VK** not only in the deep‐UV region that are typical of peptides, but also peaks in the UV region suggesting the naphthalene and NDI chromophores are also in a chiral environment (Figure S6). This indicates chiral induction from the amino acids conjugated through alkyl linkers to both NDI and naphthalene chromophores. The inversion of the ellipticity of **DA1‐K** and **DA1‐VK** between 320 and 360 nm (Figure 3a) corresponds to the absorption region dominated by the naphthalene chromophore and potentially indicates an asymmetric effect on the orientation of individual chromophores. Moreover, no detectable CD signals were observed in dimethyl sulfoxide (DMSO) solutions where the DA‐PA molecules are expected to be monomeric (Figure S17), confirming that the CD signals observed in aqueous solutions of **DA1‐K** and **DA1‐VK** arise from desymmetrization induced by supramolecular self‐assembly. The less intense absorbance in the CT band region for **DA1‐K** and **DA1‐VK** in DMSO further supports the statement that the observed CT interactions predominantly arise from intermolecular interactions within the supramolecular assemblies rather than intramolecular processes (Figure S17). Two‐photon confocal microscopy was then utilized to establish which samples exhibit second harmonic generation (SHG), in order to identify the non‐centrosymmetric compounds [15]. **DA1** did not give rise to SHG (Figure S18), suggesting centrosymmetric crystallization as expected for a molecule that lacks obvious symmetry‐breaking features. SHG activity was observed for **DA1‐K**, **DA1‐VKVK** (Figures S19 and S20), and **DA1‐VK** (Figure 3b), confirming that crystalline ordering in these assemblies is non‐centrosymmetric. Compared to **DA1‐K** and **DA1‐VK**, the *β*‐sheet in **DA1‐VKVK** (Figure 3c) gives rise to a blue shift of the crossover point in ellipticity inversion and stronger CD signals in the absorption region of NDI moieties, which may indicate enhanced chiral induction to the chromophores (Figure S20). Fourier‐transform infrared spectroscopy (FTIR) of the **DA1** series (Figure 3c) confirms the presence of the amide I band (1620–1630 cm^−1^, C = O stretching) corresponding to *β*‐sheet hydrogen bonding formation in **DA1‐VK** and **DA1‐VKVK**. The carbonyl stretching of the NDI acceptor bands at 1705 cm^−1^ and 1655 cm^−1^, which has been used to estimate the degree of charge transfer [32], shows a blue shift and diminished intensity. The blue shift of carbonyl stretching bands indicates weakening of the donor‐acceptor complexation [33] as a result of the influence of the tetrapeptide headgroup (Figure 3d). These spectral changes provide additional evidence that CT interactions in DA‐PAs are predominantly assembly‐dependent and arise from intermolecular processes. CD and SHG measurements show that the chiral headgroups of **DA2‐K** and **DA2‐VK** also affect the CT interactions of the chromophores. As expected, **DA2** does not reveal CD or SHG signals, while induced chirality is observed in the chromophore absorption range of CD spectra for **DA2‐K** and **DA2‐VK**. **DA2‐VK** shows SHG activity (Figures S18 and S21), which depends on the effects of both the chiral dipeptide and its symmetry breaking in the crystalline sublattice of **DA2**.

To evaluate the molecular, electronic and structural differences between **DA1** and **DA1‐VK** molecules in the nanostructures they form, we performed DFT geometry optimizations followed by time dependent‐DFT (TD‐DFT) calculations on both monomers and clusters of four molecules arranged in the antiparallel donor‐acceptor configuration. The TD‐DFT calculations yielded theoretical UV‐Vis spectra for monomers (Figure S23) and clusters (Figure S24), with the strongest vertical excitation at ∼ 350 and ∼ 460 nm, in close agreement with the absorbance spectra of **DA1** acquired in DMF and aqueous solutions, respectively (Figure 1d), thereby validating the optimized geometries. The **DA1‐VK** cluster structure (Figure S25a) exhibited small changes in the D–A core, including reduced aromatic stacking distances (Figure 3e,f) and altered D‐A twist angles (Figure S26) compared to those in **DA1** molecules, likely arising from hydrogen bonding between VK peptide segments (Figure S25b). The VK peptides adopted asymmetric arrangements on either side of the D‐A core, indicative of conformational flexibility with similar energetic contributions from stable hydrogen bonds. Stronger hydrogen bonds with shorter hydrogen bond donor–acceptor distances were observed further away from the D–A core, indicative of the stronger peptide interaction towards the peptide amidated C‐termini (Figure S25b), where the influence of mismatched π–π stacking distances diminishes. These DFT calculations support a peptide‐induced symmetry breaking in the charge‐transfer regions of DA‐PA assemblies as part of the mechanism leading to ferroelectricity.

To further verify the chiral induction from the dipeptide headgroups, we mixed the chiral DA PAs with their enantiomers. For this purpose, we synthesized DA2‐(D) valine‐(L) lysine (**DA2‐(D)V‐K**), DA2‐(L) valine‐(D) lysine (**DA2‐V‐(D)K**), and DA2‐(D) valine‐(D) lysine (**DA2‐(D)V‐(D)K**). Different racemized systems were prepared by co‐assembly of **DA2‐(D)V‐(D)K**/**DA2‐VK**/**DA2‐(D)V‐K**/**DA2‐V‐(D)K** in a 1:1:1:1 ratio (**DD/LL/DL/LD**), **DA2‐(D)V‐K**/**DA2‐V‐(D)K** in a 1:1 ratio (**DL/LD**), and self‐assembled structures of **DA2‐(D)V‐K**, and **DA2‐V‐(D)K**. The samples revealed the formation of crystalline ribbons after annealing and are found to be stable in the dried state (Figure S27). CD spectra showed decreased chirality in co‐assemblies of the racemic PAs and the mixture of **DD/LL/DL/LD** shows the least chirality. Two‐photon confocal microscopy did not generate any SHG signal in **DL/LD** and **DD/LL/DL/LD** (Figure S29), confirming the elimination of chirality in these racemized mixtures. The co‐assembly of the enantiomers not only eliminated the net chirality of the entire sample but also excluded the possibility of any local noncentrosymmetric crystal structures, suggesting that the long‐range *β*‐sheet structures with one chiral handedness are necessary for symmetry breaking of the CT sublattice in the DA‐PA assemblies.

## 5 Ferroelectricity in DA‐PA Nanostructures

**FIGURE 4:**
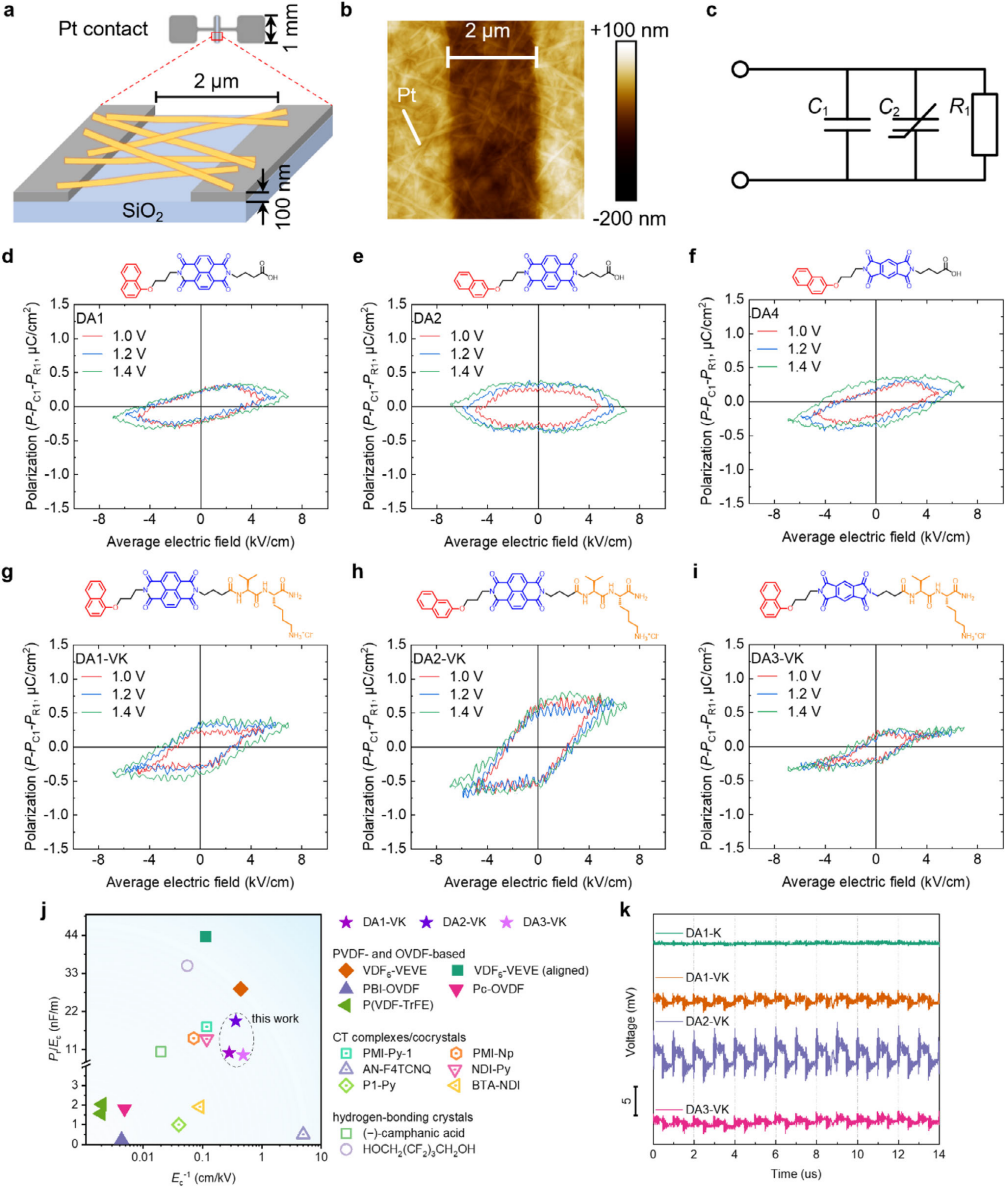
Ferroelectricity in donor–acceptor peptide amphiphile nanostructures. a, Schematic of the ferroelectric testing device. b, Atomic force microscopy image of the **DA2‐VK** coating on the test chip. **c**, Schematic diagram of the equivalent circuit for a measured sample containing a linear capacitor *C*_1_ in parallel with a non‐linear capacitor *C*_2_ and a resistor *R*_1_. d‐i, P‐E loops of **DA1** (d), **DA2** (e), **DA4** (f), **DA1‐VK** (g), **DA2‐VK** (h), and **DA3‐VK** (i), and the corresponding molecular structures. j, Comparison of *P*_r_/*E*_c_ vs. *E*_c_^−1^ for ferroelectric DA‐PA assemblies and other metal‐free room‐temperature organic ferroelectrics. See Table S2 for numerical values of *P*_r_ and *E*_c_ and definitions of the abbreviations. k, Piezoelectric voltage waveforms of the DA‐PA samples under ultrasonic stimulation (see the fast Fourier transform analysis in Figure S43). The voltage curves in (k) are offset vertically for clarity.

Based on the observed structural features of these systems, we were interested in exploring the potential use of these materials as thin‐film organic ferroelectrics. Polarization vs. electric field curves (P‐E loops) were collected on dried drop‐cast samples of DAs and DA‐PAs using photolithographically patterned platinum‐electrode test chips with a 2 µm gap on a SiO_2_ substrate (Figure 4a,b). Measurements of P‐E hysteresis loops require the removal of the contribution of conduction loss *P_R_*_1_ and linear capacitance *P_C_*_1_ to reveal the nonlinear ferroelectric component (*C*_2_) (Figure 4c, see Methods). Given the nonuniform electric field generated by the in‐plane electrodes, the polarization strength was calibrated using our previously reported method [22], and the P‐E loops were plotted using the average electric field applied across the electrode gap. Further details can be found in the Experimental Section [22]. As shown in Figures 4g‐h, the P‐E loops of **DA1‐VK** and **DA2‐VK** samples cast from annealed solutions exhibit coercive fields (*E*_c_) of about ±2.5 kV/cm and significant remnant polarization (*P*_r_), demonstrating ferroelectric properties of the nanostructures at room temperature. The *P*_r_ of **DA2‐VK** is higher than **DA1‐VK** although they have similar donor‐acceptor moieties. This is most likely due to the disruption of the extended ribbons of **DA1‐VK** into predominantly small platelet‐like structures with an average length below 1 µm (Figures S10c and S35), rendering them incapable of bridging the 2 µm gap between the electrodes. In contrast, **DA2‐VK** forms crystalline ribbons averaging approximately 3 µm in length along the polar CT axes (Figure 4b, Figures S10c and S14e). We did not observe hysteresis for the P‐E loops of unannealed **DA2‐VK** (Figure S36), which shows small fibrous nanostructures (Figure S22), demonstrating that the long nanoribbon structures are necessary for the ferroelectric polarization of the DA‐PA samples. When we changed the NDI electron acceptor moiety to PMDI in **DA3‐VK**, smaller P‐E loops were obtained with an *E*_c_ of about ±1.5 kV/cm and lower remnant polarization strengths compared to **DA1‐VK** and **DA2‐VK** (Figure 4i), even though large bundles of the assemblies were observed in the dried **DA3‐VK** samples (Figure S37). This behavior may be attributed to the weak CT interactions of the oblique lattice structures formed within **DA3‐VK** assemblies (Figure S15). As shown in Figure 4j, ferroelectric **DA‐PA** assemblies exhibit a comparable low‐field ferroelectric polarization (*P*_r_/*E*_c_) to the recently reported high‐performance ferroelectric CT complexes and other metal‐free room‐temperature supramolecular ferroelectrics (Table S2). We further examined the piezoelectric responses of DA‐PA assemblies under ultrasonic stimulation using an ultrasound therapy device (see Experimental Section and Figure S42). As shown in Figure 4k, ferroelectric DA‐VK samples generate piezoelectric voltage waveforms that match the device's working frequency of 1.0 MHz. In contrast, the non‐ferroelectric **DA1‐K** sample exhibits negligible voltage signals. Fast Fourier transform (FFT) of the waveforms reveals that **DA2‐VK** exhibits the highest piezoelectric voltage (Figure S43) compared to **DA1‐VK** and **DA3‐VK** samples, which correlates well with its observed highest remnant polarization considering the electrostriction approximation (see Supporting Information below Figure S43).

Measurements of P‐E loops for **DA1**, **DA2**, and **DA4** did not reveal any stable ferroelectric polarization (Figures 4d‐f) but exhibited lossy dielectric behavior featuring cigar‐shaped loops instead [34]. This is not surprising given the lack of obvious symmetry‐breaking elements in these and the absence of any observed SHG activity. Measurements of **DA1‐VKVK** samples also did not reveal any evidence of ferroelectricity (Figure S36). This is attributed to the weak CT interactions disrupted by the strong *β*‐sheets formed by tetrapeptides as demonstrated by FTIR (Figure 3c,d) and UV–vis spectra (Figure 1f). WAXS pattern for **DA1‐VKVK** reveals a hydrogen‐bonding spacing of 4.65 Å (Figure S13), which is very different from the intermolecular spacing of **DA1** (*a* = 7 Å and *b*/2 = 8 Å, as illustrated in Figure S11). This results in low crystallinity within the fibrous nanostructures (Figure S13), which must not be forming ferroelectric domains of significant dimension, if any. Similar competition of amide hydrogen bonding over π–π stacking interactions were observed in our previous studies on other conjugated systems, leading to reduced structural order [35]. Interestingly, despite similar chirality signals in CD and SHG activity as **DA1‐VK**, **DA1‐K** does not exhibit ferroelectric switching but instead exhibits lossy dielectric behavior, and neither do **DA2‐K**, **DA3‐K**, or **DA4‐K** (Figure S38). This is because the monopeptide lysine, which does not form *β*‐sheet crystals during annealing in water (Figure 3c), cannot induce sufficient asymmetry to perturb the crystalline structure of the chromophore molecular segment, as demonstrated by SAED of **DA1‐K** (Figure 2d). For the racemized systems, ferroelectric hysteresis is not observed in the P‐E loops of **DL/LD** and **DD/LL/DL/LD** (Figure S40). We conclude that the peptide termini introduce symmetry breaking in the packing of molecules within the assemblies which is required for ferroelectricity in the donor‐acceptor systems. Furthermore, we found that the morphology and polarization strength was reproducible across different batches of ferroelectric DA‐PA assemblies (Figure S44), indicating robust processing conditions and potential for scalable fabrication.

## 6 Primary Neuron Growth on DA‐PA Coatings

**FIGURE 5:**
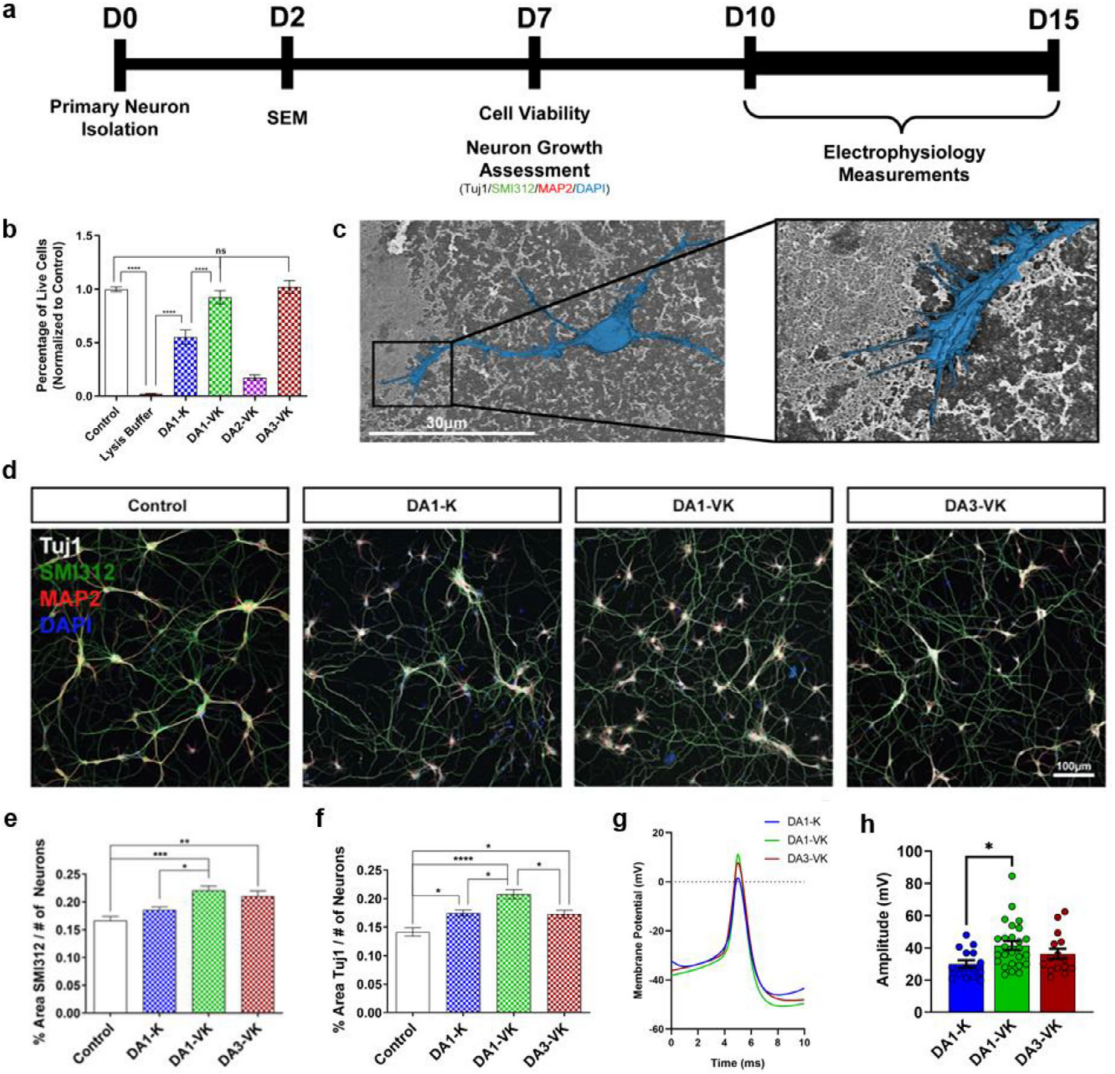
Primary cortical neuron survival and growth supported on coatings of donor–acceptor peptide amphiphile nanostructures. a, A schematic timeline for neuron experiments. b, Ratio of live to total cells (normalized to the control) after primary neurons were cultured for one week on coatings of DA‐PA material. Visualized live and dead cells are shown in Figure S48. **c**, Representative scanning electron microscopy (SEM) micrographs of a primary neuron (false‐colored in blue) cultured on a **DA3‐VK** coating for 48 h. d, Representative confocal images of neurons grown on DA‐PA coatings for one week. Primary neurons were stained with Tuj1 (neural cytoskeleton, white), MAP2 (mature dendrites, red), SMI312 (mature axons, green), and DAPI (nuclei, blue) (a PDL coating control was used). e, Quantification of the percent area of SMI312‐positive neurite coverage per field normalized to the number of DAPI‐positive nuclei. f, Quantification of the percent area of Tuj1‐positive neurite coverage per field normalized to the number of DAPI positive nuclei. g, Representative action potential traces of neurons cultured on DA‐PA coatings for 10–15 days. h, Quantification of the action potential amplitude of neurons cultured on DA‐PA coatings for 10–15 days. For (b, e, f, h) a one‐way ANOVA with a Tukey's multiple comparisons test (α = 0.05) was performed: (*) p < 0.05, (**) p < 0.01, (***) p < 0.001, (****) p < 0.0001.

Because of their inherent surface polarization capable of electrostatically binding bioactive ions and proteins, ferroelectric and piezoelectric materials have already been used to improve cell growth [36, 37], differentiation [38, 39, 40], and migration in vitro and in vivo in bone, neural, cardiac, and skin tissues [9, 13, 41]. However, most inorganic ferroelectric materials have limited clinical relevance due to their mechanical rigidity and the presence of toxic metals such as lead in some of the common materials (e.g., Pb(Zr,Ti)O_3_). In this context, further development of soft organic ferroelectric materials for biological applications is an interesting area but remains limited at this point [13, 42, 43]. To determine whether our novel organic DA‐PA materials exhibit any biological activity, we investigated the response of primary cortical neurons in vitro to ferroelectric (**DA1‐VK, DA2‐VK, DA3‐VK**) and non‐ferroelectric (**DA1‐K, DA1‐VKVK**) DA‐PA coatings (Figure 5a). The results showed that the ferroelectric **DA1‐VK** and **DA3‐VK** had similar levels of viability relative to the commonly used poly‐D‐lysine (PDL) coating as the control sample, in fact, higher than the non‐ferroelectric **DA1‐K** compound, while the ferroelectric **DA2‐VK** and non‐ferroelectric **DA1‐VKVK** did not support neuron survival (Figure 5b). The reduced cell viability observed on **DA2‐VK** and **DA1‐VKVK** samples is attributable to the inherent cytotoxicity of the naphthalene moieties [44]. Although hydrophobic naphthalene units are embedded between two layers of peptides and charged groups that effectively shield them from cellular exposure on the top and bottom surfaces of the ribbon‐like assemblies, the narrow (**DA2‐VK**) and fibrous (**DA1‐VKVK**) assemblies (Figure S10b) may allow increased exposure of naphthalene moieties to cells along their lateral edges. This structural feature could contribute to the lower cell viability observed on these samples compared to wider ribbon‐like assemblies. Neurons grown on **DA1‐K**, **DA1‐VK,** and **DA3‐VK** coatings possessed extended neural processes and showed signs of growth cone formation, suggesting favorable binding and growth along the coating surface (Figure 5c, Figure S47) [45]. Moving forward, we were interested in establishing whether the biocompatible and ferroelectric **DA1‐VK** and **DA3‐VK** coatings were also able to enhance neurite growth and complexity. After one week (Figure 5d), neurons grown on the ferroelectric **DA1‐VK** and **DA3‐VK** coatings exhibited larger surface areas of axonal networks (SMI312 marker), normalized to the number of DAPI‐positive (nuclear 4,6‐diamidino‐2‐phenylindole stained) cells per field of neurites (Figure S50b), compared to either the PDL control or the non‐ferroelectric **DA1‐K** (Figure 5e). Additionally, Figure 5d shows that **DA1‐VK** and **DA3‐VK** coatings reveal a significant increase in β‐tubulin III surface area relative to the PDL control (Figure 5f). This marker, also known as Tuj1, is a microtubule protein marker for neuronal identity and is important in axon guidance. These results suggest that the biocompatible ferroelectric DA‐PA coatings can enhance neurite and axon growth without the need for external electrical or mechanical stimulation [11].

We also investigated the electrophysiological activity of neurons grown on DA‐PA coatings for 10–15 days by measuring action potential (AP) properties using whole‐cell current‐clamp techniques (Figure S51a). We found that neurons cultured on **DA1‐VK** had a statistically greater AP amplitude than those cultured on non‐ferroelectric **DA1‐K** (Figures 5g,h). Considering that the spontaneous polarization of ferroelectric DA‐PA nanostructures is aligned with their long π‐π stacking axes, we propose that the electric field induced by the ferroelectric DA‐PA nanostructures electrostatically binds and protects secreted proteins (such as neurotrophic growth factors) and ions present in the media with a concentration gradient along the length of the DA‐PA fibers. This ferroelectric‐mediated distribution of bioactive ions, similar to the gradients of proteins that guide neuron growth in the central nervous system [46, 47, 48, 49, 50], can stimulate neuronal maturation.

## 7 Conclusion

We have reported on donor‐acceptor peptide amphiphile molecules that self‐assemble in water to form crystalline nanostructures, in which the chirality of the *β*‐sheet forming peptides induces symmetry breaking within the donor‐acceptor lattice, leading to second harmonic generation and ferroelectricity. Variations in the donor‐acceptor moieties validate this peptide‐induced symmetry‐breaking approach for developing new ferroelectric materials from charge transfer systems. Primary cortical neurons cultured on the ferroelectric DA‐PA coatings exhibit enhanced neurite growth. Electrophysiological experiments further highlight that neurons grown on the ferroelectric DA‐PA coatings show enhanced AP features, indicating a more functionally mature culture. Our findings suggest a versatile design strategy for new biocompatible organic ferroelectric materials that could be interfaced with neural tissues for bioelectronic functionality. Given that our DA‐PA materials can be processed via low‐temperature self‐assembling in aqueous solution, they are compatible with existing flexible substrates and microfabrication techniques, offering potential for flexible electronics, biosensing, and low‐power devices.

## 8 Experimental Section

All starting materials were purchased from MilliporeSigma and all solvents from Fisher Chemical unless otherwise noted. Chromatography was performed using silica gel as the stationary phase.

### 8.1 Crystallographic Characterization

Crystals of **DA1**, **DA2,** and **DA3** suitable for X‐ray diffraction were grown through slow evaporation of dimethyl sulfoxide solutions for **DA1** and **DA3**, and *N*,*N*‐dimethylformamide solutions for **DA2**. Single‐crystal X‐ray diffraction measurements were collected on a Rigaku Cu‐Synergy Instrument equipped with a CuKα microfocus source, hybrid photon counting detector, and Oxford cryostream system at 100 K. Data collection, integration, and space group determination were performed using CrysAlis Pro. Phasing, initial solution, and structure refinement were performed using SHELXT through the Olex2 graphical user interface. Crystallographic details are provided in the Supporting Information. Crystallographic data for the structures reported in this paper have been deposited with the Cambridge Crystallographic Data Centre under deposition numbers CCDC 2476618–2476620.

### 8.2 Solution Preparation

Solutions of **DA1** and **DA2** were prepared by dissolving each powder in deionized (DI) water to achieve the desired concentration followed by the addition of 1 equivalent of sodium hydroxide using a 250 mM stock solution. The solutions were then sonicated using a bath sonicator for 10 min and the solid was found to completely dissolved. Subsequently, the solutions were annealed at 80°C in a water bath (approximately 3 L) for 30 min, followed by a slow cooling to room temperature. Solutions of **DA4** were prepared in the same way as **DA1** and **DA2** except that the solutions were not annealed, since annealing was found to cause degradation of the molecules. Conditions were not found to effectively dissolve **DA3** in water. Solutions of DA‐PA samples were prepared by adding the appropriate amount of DI water to the solid powder to reach the indicated concentrations, followed by the same sonication and annealing procedures as applied for **DA1** and **DA2**. All DA‐PA samples were annealed before analysis unless specifically mentioned otherwise. For the co‐assembly of the racemized peptide amphiphiles, the mixtures of the molecules were first dissolved in HFIP (5 mM), dried by rotary evaporation, dissolved in DI water (5 mM), and then annealed following the aforementioned procedures.

### 8.3 Transmission Electron Microscopy (TEM)

All TEM experiments were carried out on a JEOL 1230 TEM fitted with a tungsten filament at 100 kV, using a Gatan 831 CCD camera. Samples were typically prepared from 1 mM starting concentrations, which were diluted 20 times with DI water no more than 10 min before grid preparation. A volume of 5 µL of the diluted samples was applied to EMS CF200‐Cu grids. After 30 s, the liquid was wicked away, followed by the addition of 2 wt% uranyl acetate. After another 30 s, the liquid was wicked away, and 5 µL of DI water was added and left for 1 min. The liquid was wicked away and the grid was allowed to dry in air for at least 30 min.

### 8.4 Ultraviolet‐Visible Absorption Spectroscopy

The typical solution concentration utilized for optical absorption is 0.2 mM, prepared by five‐fold dilution of the 1 mM DA or DA‐PA solutions. All UV–vis absorption spectroscopy was carried out using a 2 mm path length quartz cuvette and an Ocean Optics QEPro Spectrophotometer.

### 8.5 Circular Dichroism Measurements

Samples were prepared by diluting DA or DA‐PA solutions to 50 µM before measurements. Measurements were conducted using a Jasco J‐815 CD instrument with a 2 mm path length quartz cuvette. Data were collected from 190 to 500 at 2 nm intervals. Linear dichroism (LD) spectra of DA1‐PA samples were also measured to rule out LD contributions to CD signals.

### 8.6 Second Harmonic Measurements

Two‐photon confocal microscopy images were obtained using an adapted procedure [15] on a Nikon A1R MP+ Multiphoton Confocal Microscope, equipped with a Coherent Ti:Sapphire Chameleon Vision S Laser, mode‐locked at 80 MHz with a 75 fs pulse duration. The incident laser wavelength was tunable from 690 to 1040 nm. Reflected and transmitted photons were recorded by detectors above and below the sample plane, with detection windows from 380 to 450 nm and 470 to 500 nm. The irradiation power across all incident wavelengths was set at 800 mW. Two‐photon confocal microscopy images and emission spectrum on racemized samples were obtained using a Leica SP8 Multiphoton microscope with detection windows from 380 to 500 and 400 to 450 nm corresponding to the full range and SHG active range of the emission spectrum at the excitation wavelength of 850 nm. Emission intensity spectrum data were acquired every 10 nm. Dried films of DA and DA‐PA samples were sandwiched between a glass microscope slide and a coverslip to separate the crystals from the water‐immersion objective lens. All samples were irradiated in the dark at room temperature under ambient conditions.

### 8.7 DFT Calculations

DFT and TD‐DFT calculations were performed using Gaussian 16 Revision C.01. Initial structures for **DA1** and **DA1‐VK** were constructed using Avogadro 1.2.0 [51]. Geometry optimization for the single molecules was performed using the wB97XD functional [52] at the 6–31G+(d,p) level of theory with a water (ε = 78.3553) solvation model implemented with SMD (solvation model based on density) [53]. TD‐DFT calculations were also performed using the same set of parameters on the optimized geometry. Gaussview 6 was used for visualization of the optimized structures and the HOMO/LUMO molecular orbitals. Using the crystal structure for **DA1**, a cluster of four molecules (252 atoms/1120 electrons/‐ 4 charge) was used to perform a geometry optimization at the 6–31G(d,p) level of theory using the same functional and solvent model described above. This geometry optimized **DA1** core was then substituted with VK‐NH_2_ peptides and further geometry optimization was carried out for a cluster of **DA1‐VK molecules** (412 atoms/1616 electrons/+ 4 charge). This was performed using the computationally feasible B3LYP functional with an additional D3 version of Grimme's empirical dispersion with Becke–Johnson damping [54] at the 6–31G(d) level of theory. Optimized geometries were visualized and rendered using VMD 1.9.3 [55], which was also used to measure distances, angles, and also to identify hydrogen bonds.

### 8.8 Ferroelectric Devices Preparation and P‐E Loop Measurements

DA and DA‐PA samples at either 1 or 5 mM were deposited on OFET test chips with a 300 nm thick SiO_2_ insulation bottom layer and photolithographic platinum electrodes of 2 µm channel width (Ossila, S403A1). The cross‐section of the electrode is 1 mm wide and 100 nm thick. The samples were dried in a vacuum overnight before electrical tests. Polarization hysteresis loops were measured using a ferroelectric tester (Radiant Technologies Precision LC) at room temperature. Double bipolar triangle waveforms were applied with a single loop period of 50 ms, a pre‐loop delay between the two loops of 10 ms, and maximum voltages of 0.8∼1.4 V. The second loop was recorded as the original P‐E loop data. The resistance of the samples, *R*_1_, was measured using the ferroelectric tester. A DC voltage of 1 V was applied while the leakage current was recorded for 10 s until the polarization process was complete. The frequency‐domain impedance spectrum was measured using AutoLab PGSTAT‐128N (voltage amplitude of 0.05 to 0.5 V) and Solartron 1260 (voltage amplitude of 0.2 to 1.0 V) at room temperature to obtain the linear capacitance of the sample, *C*_1_. The ferroelectric component was obtained after subtracting the contribution of *C*_1_ and *R*_1_ from the original P‐E loop (see Section S6.2). Given the nonuniform electric field generated by the in‐plane electrodes, the polarization strengths (*P*‐*P*_C1_‐*P*_R1_) of the P‐E loops were multiplied by a calibration coefficient equal to 0.45 based on the measured thicknesses of the samples (around 500 nm, measured by AFM), according to the method reported in our previous study using the same experimental setup [22]. In addition, the coercive field values observed on the average electric field axes of the P‐E loops presented here should be calibrated by multiplying by a coefficient equal to 1.53 [22].

### 8.9 Device Preparation and Piezoelectric Response Measurements

DA‐PA coatings (**DA1‐K**, **DA1‐VK**, **DA2‐VK**, **DA3‐VK**), with thicknesses of about 50 µm, were prepared by repeatedly casting 5 mM annealed aqueous solutions onto individual channels of interdigitated indium tin oxide (ITO) electrodes. These channels were isolated by a plastic protective membrane on the ITO‐patterned surface of the glass substrate (Ossila, S161‐20) (Figure S42a). Each ITO electrode channel features a constant gap size of 50 µm and a length of 30 (5×6) mm (Figure S42b). The ITO electrodes were connected to the four probes (AC coupling) and the ground of an oscilloscope to record piezoelectric voltage signals. To apply uniform ultrasonic stimulation to the samples, a 2‐mm‐thick foam spacer was inserted between the ITO glass substrate and an ultrasound therapy device (US Pro 2000, Richmar), with a square void filled with conductive gel under the measured region (Figure S42c). The piezoelectric responses of all DA‐PA samples were recorded at the same time under an ultrasonic stimulation at an output power of 0.32 W (±20%) (power mode: L) and a working frequency of 1.0 MHz (±10%). For a more quantitative comparison of the piezoelectric voltages, a 4096‐point fast Fourier transform (FFT) of the voltage waveforms were performed (Figure S43).

### 8.10 Coating Procedure for In Vitro Assessment

A solution of 0.01 mg/mL poly‐D‐lysine hydrobromide (MW 70,000‐150,000; Sigma–Aldrich, Cat. No. P6407) was prepared in DI water and applied to glass coverslips or tissue culture treated plastic well plates. These were incubated at 37°C overnight. The poly‐D‐lysine hydrobromide solution was then removed, and the coatings were washed twice with sterile DI water and left to dry overnight prior to in vitro studies. A 5 mM DA‐PA solution, prepared as previously described, was diluted to 1 mM in DI water immediately prior to coating preparation. 100 µL of 1 mM DA‐PA solution was carefully pipetted onto PDL coated glass coverslips or PDL coated tissue culture treated plastic well plates and allowed to dry overnight (Figure S45). DA‐PA coatings were prepared and found to remain after 48 h under cell culture conditions (Figure S43).

### 8.11 IACUC Standard Statement

All animal housing and procedures were performed in accordance with the Public Health Service Policy on Humane Care and Use of Laboratory Animals. All procedures were approved by the Northwestern University Institutional Animal Care and Use Committee.

### 8.12 Neuron Media Preparation

250 µL penicillin/streptomycin (Sigma–Aldrich, Cat. No. P4333), 70 µL L‐Glutamine (Gibco, Cat. No. 25030081), 155 µL sodium bicarbonate (Gibco, Cat. No. 25080084) and 1.25 mL of NHS (Gibco, Cat. No. 16‐050‐122) were added to 25 mL of neurobasal media (Gibco, Cat. No. 21103049) to create *pre‐plating media*. 500 µL penicillin/streptomycin, 125 µL L‐Glutamine, 290 µL sodium bicarbonate, 500 µL NHS and 500 µL glutamate (370 µg/mL milliQ, Millipore Sigma, Cat. No. 49621) were added to 50 mL of neurobasal media to create *Media 1*. 500 µL penicillin/streptomycin, 125 µL L‐Glutamine and 290 µL sodium bicarbonate were added to 50 mL of neurobasal media to create *Media 2*. All media were sterile‐filtered through a 0.22 µm membrane (Millipore, Cat. No. S2GPU05RE, Cat. No. SCGP00525). The media were then incubated at 5% CO_2_ and 37°C for at least 1 h. 1 mL of B27 supplement (Gibco, Cat. No. 17504044) was added to *Media 1* and *Media 2* immediately prior to use.

### 8.13 E16 Primary Mouse Cortical Neuron Dissection and Culture

E16 primary mouse cortical neurons were dissected from embryonic mouse brains following a previously established protocol, summarized as follows [56]: Embryos were removed from a timed pregnant CD1 mouse (Charles River), sacrificed by cervical dislocation, at embryonic day 16 (E16). Dissected cortices were preserved in ice‐cold Hanks’ Balanced Salt Solution (Gibco, Cat. No. 14‐170‐112) supplemented with 1% penicillin/streptomycin throughout the dissection until they were transferred into 5 mL of 0.25% Trypsin/EDTA (Gibco, Cat. No. 25200072) solution with 250 µL of 4000 U/mL DNAse I (Worthington Biochemical, Cat. No. LS002006). After 10 min, cortices were placed and then mechanically dissociated in 5 mL of pre‐plating media and 250 µL of 4000 U/mL DNAse I. The supernatant was removed and placed into a new Falcon tube to remove debris. The dissociated cortices were then centrifuged for 5 min at 1000 rpm. After the supernatant was removed, cells were first resuspended before being transferred into a cell culture‐treated flask filled with additional pre‐plating media. The flask was incubated for 40 min at 37°C to improve the culture purity. Glial cells attached to the tissue‐culture treated surface of the flask while embryonic cortical neurons remained in suspension. Suspended neurons were next filtered through a 100 µm cell strainer (Corning, Cat. No. CLS431752) before being centrifuged again for 5 min at 1000 rpm, after which the supernatant was removed, and the pellet resuspended into media 1. A 1:50 dilution of the cell solution to media 1 was prepared to count the total number of cells with a hemocytometer. After seeding, cells were maintained at 5% CO2 and 37°C for 24 h All of *media 1* was then removed and then replaced with *media 2*. Half of the media was exchanged with fresh m*edia 2* every 4–5 days for the duration of the experiment.

### 8.14 Scanning Electron Microscopy (SEM)

E16 primary cortical neurons cultured on DA‐PA coatings for 48 h were fixed in a 2.5% glutaraldehyde (Electron Microscopy Sciences, Fisher Scientific, Cat. No. 50‐262‐18)/2% paraformaldehyde (Electron Microscopy Sciences, Fisher Scientific, Cat. No. 043368.9 M) solution prepared in 1x PBS (Gibco, Cat. No. 10010) for at least 20 min. Fixed samples then underwent an ethanol exchange. Samples were incubated in a series of ethanol solutions every 10 min. The following ethanol solutions were used for this process, in order: 30%, 40%, 50%, 60%, 70%, 80%, 85%, 90%, 95% and 100%. Samples were left in 100% ethanol for at least 30 min prior to being critical point dried. Critical point drying (CPD) was performed, with a purge time of 20 min, on a Tousimis Samdri‐795. Dried samples were preserved under vacuum until the day of SEM imaging. Immediately prior to imaging, samples were coated with two layers of osmium using an SPF osmium coater. Osmium was deposited at a thickness of 8 nm per layer, resulting in a final osmium coating thickness of 16 nm. SEM imaging was performed on a Hitachi SU8030 located in the NUANCE facility at Northwestern University at an accelerating voltage of 2.0 kV.

### 8.15 Cell Viability Experiments

E16 primary cortical neurons were seeded directly onto DA‐PA coatings at a density of 60,000 cells per well in a 24‐well plate. Neurons were stained with acridine orange (AO) and propidium iodide (PI) using a Cyto3D Live‐Dead Assay Kit (TheWell Bioscience, #BM01) to visualize live and dead cells. Imaging and analysis were performed using an Essen Bioscience IncuCyte S3 located in the ANTEC facility at Northwestern University.

### 8.16 Immunocytochemistry

E16 primary cortical neurons were cultured on DA‐PA coatings for 48 h or 1 week. At the desired endpoint, samples were fixed for 10 min in 4% paraformaldehyde (Fisher Scientific, Cat. No. 043368.9 M). Subsequently, samples were washed twice with 1x PBS (Gibco, Cat. No. 10010) and once with a 1x PBS blocking buffer supplemented with 5% NHS (Gibco, Cat. No. 16‐050‐122) and 0.1% Triton X‐100 (Fisher Bioreagents, Cat. No. BP151‐500). The samples were then incubated in a fresh blocking buffer for 2.5 to 3 h before applying the primary antibody solution. The desired primary antibody solution, prepared in blocking buffer, was added to the samples and then left to incubate at room temperature for 30 min before being moved to 4°C overnight. The next day, coverslips were removed from 4°C and incubated at room temperature for 30 min. They were then washed three times with a blocking buffer for 15 min each. Next, samples were incubated with 350 µL of secondary antibody solution prepared in a blocking buffer for a minimum of 2 h, protected from light. Following one wash in blocking buffer, samples were incubated for 10 min with the nuclear stain DAPI (1:1000, ThermoFisher, D1306), prepared in blocking buffer. Samples were then washed three times with blocking buffer and three times with 1x PBS for 20 min each. Coverslips were immediately mounted onto glass slides with Immuno‐Mount solution (ThermoScientific, Cat. No. 9990402) and stored at 4°C until imaging. All confocal images were taken on the Nikon A1R (B) GaAsP at the Center for Advanced Microscopy/Nikon Imaging Center (CAM) at Northwestern University. ***Primary antibodies***: 1:2000 MAP2 (Rb, Biolegend, #840601), 1:1000 SMI312 (Ms, Biolegend, #NC1239357), and 1:1000 Tuj1 (Chk, Abcam, #ab41489). ***Secondary Antibodies***: 1:1000 Alexa Fluor 488 (Ms, Invitrogen, A‐21202), 1:1000 Alexa Fluor 555 (Rb, Invitrogen, A‐31572), and 1:1000 Alexa Fluor 647 (Chk, Invitrogen, A‐21449).

### 8.17 Neuron Morphometric Analysis

Primary axon length was measured using the “NeuronJ” plugin in Fiji. The longest SMI312‐positive neurite on SMI312‐positive neurons was traced. The number of cell aggregates per area was manually quantified in Fiji using the “Cell Counter” plugin. Cell aggregates were defined as three or more cells clustered together. The number of DAPI‐positive cells per area was manually counted using the “Cell Counter” plugin in Fiji. To analyze the network area of Tuj1‐positive neurites after 1 week in culture, maximum projection images of the channel containing the protein of interest were processed by first applying the “Tubeness” filter (σ = 0.6), then a Huang threshold (over/under), and then auto‐converting the image into a mask. The area fraction, or the percent of the image covered in highlighted pixels, was quantified using the “Measure” function in Fiji. This value was then normalized to the number of DAPI‐positive cells present in the image, which was counted manually as described above. To analyze the network area of SMI312‐positive neurites after 1 week in culture, maximum projection images of the channel containing the protein of interest were processed by first applying a Gaussian Blur (σ = 2) followed by the “Tubeness” filter (σ = 0.6). A Huang threshold (over/under) was then applied before auto‐converting the image into a mask. The area fraction, or the percent of the image covered in highlighted pixels, was quantified using the “measure” function in Fiji. This value was then normalized to the number of DAPI‐positive cells present in the image, which was counted manually as described above.

### 8.18 Current Clamp

Cortical neurons from E16 mouse embryos were isolated on “day 0” and cultured on glass coverslips coated with **DA1‐K**, **DA1‐VK**, or **DA3‐VK** or a PDL coating control. At 10–15 days post‐dissection whole‐cell current‐clamp recordings were performed as previously described [57]. Neurons were visually identified using an inverted Olympus IX51 microscope equipped with a 40X objective. Recording pipettes were made of glass capillaries using a horizontal Sutter P‐1000 puller yielding a 2–4 MΩ resistance pipette when filled with standard K‐methyl sulfate intracellular solution containing (in mM): 120 K‐MeSO_4_, 10 KCl, 10 HEPES, 10 Na2‐phosphocreatine, 4 Mg‐ATP, 0.4 Na3‐GTP, pH 7.35 adjusted with KOH; osmolality 285–290 mOsm/Kg. The recording chamber was continuously perfused with oxygenated aCSF bath solution (in mM): 125 NaCl, 26 NaHCO_3_, 2.5 KCl, 1.25 NaH_2_PO_4_, 1 MgSO_4_, 22 glucose, 2 CaCl_2_, pH 7.35 at 32 to 35°C; osmolality 310–315 mOsm/Kg. Current‐clamp recordings were acquired using a Multiclamp 700B amplifier (Molecular Devices, USA) and Clampex 11.1 software (Molecular Devices, USA). Signals were digitized with a sampling rate of 10 kHz (filtered at 3 kHz). Access resistance was monitored throughout recording and cells were excluded from analysis if the value significantly deviated from the initial measurement. Analysis of current clamp data was completed in Clampfit 11.2 (Molecular Devices, USA) and Easy Electrophysiology software (RRID:SCR_021190).

### 8.19 Current Clamp Analysis

Resting membrane potential (RMP, mV) was measured immediately after establishing a whole‐cell patch clamp configuration. All reported RMP values were corrected for the liquid junction potential, calculated to be ‐8.2 mV. Input resistance (R_input_, MΩ) was calculated from the change in voltage (∆V_m_) measured at the end of hyperpolarizing current steps (‐100 to ‐20 pA). R_input_ values were derived from the slope of the linear ordinary squares fit, with X = ∆I_m_ and Y = ∆V_m_. Cells were then held at ‐65 mV (V_hold_), and action potentials evoked using 200or 500 ms current injection ramps. Neurons were classified as “Fire” if able to evoke at least one spike with an action potential (AP) peak (mV) reaching greater than –5 mV. Neurons unable to produce a spike were classified as “No Fire”. All experimental groups had at least 50% of cells able to generate an AP (Figure S51). All neurons that met quality control criteria were considered when evaluating intrinsic cell properties, which were consistent across conditions (Figure S52a–c). Only neurons classified as “Fire” were evaluated for differences in AP property parameters (Figure S53a). Action potential properties (Figure S53a) were measured from the first action potential evoked during current ramp depolarization. The minimum current required to elicit an action potential is defined as the AP Rheobase (pA). Action potential threshold (V_thresh_) was defined as the voltage (mV) at which the first derivative of each action potential reached the cutoff value of 15 mV/ms. AP amplitude (mV) was calculated as the difference between the peak and threshold. The fast afterhyperpolarization (fAHP) was the minimum voltage value within the 10 ms following the action potential peak. The maximum rise‐ and decay‐slope values were defined as the maximum absolute dV/dt value calculated from a regression over two sample points from the first derivative. The half‐width (ms) was calculated as the time between the two half‐amplitude samples (on the rise and decay). Data was interpolated to 200 kHz for improved accuracy of rise, decay and half‐width measurements.

### 8.20 Statistical Analysis

Data analysis was performed with GraphPad Prism software (version 9.5.0). Comparisons among three or more groups were conducted using one‐way ANOVA with a Tukey's multiple comparisons test unless otherwise indicated. The statistical tests and parameters used for each experiment are reported in the corresponding figure legends. For cell viability and confocal data, error bars represent at least 30 images from 2 separate dissections. All error bars shown in graphs represent the standard error mean unless otherwise indicated. For current clamp experiments, error bars represent at least 15 neurons from three separate dissections.

## Author Contributions

J.V.P designed and synthesized the DA‐PA molecules, performed spectroscopy experiments, TEM and crystallography characterizations, and wrote the manuscript. Y.Y. performed device preparation and electrical measurements, designed the racemization experiments, and wrote the manuscript. C.S.S. performed all the in vitro experiments and some sample characterizations, analyzed the biological data, and contributed to manuscript preparation. J.H. and Y.Y. performed the data processing of P‐E loops. D.R.D performed DFT calculations. Z.A. performed two‐photon confocal microscopy. B.K.J. performed two‐photon confocal microscopy and CD measurements in racemization experiments. K.A.M performed patch clamp experiments and analyzed the data. H.S. and R.Q. performed X‐ray scattering measurements. C.L.S. contributed to crystallography characterizations. L.C.P. helped write the manuscript. E.K. supervised patch clamp experiments. S.I.S. supervised the research and wrote the manuscript. All authors contributed to data analysis and manuscript preparation.

## Conflicts of Interest

The authors declare no conflicts of interest.

## Supporting information




**Supporting File**: adma72077‐sup‐0001‐SuppMat.pdf.

## Data Availability

The datasets generated during and/or analyzed during the current study are available from the corresponding authors.
