# Supplementary material for: Peptide‐Induced Ferroelectricity in Charge‐Transfer Supramolecular Materials
Source: Adv Mater. 2026 Jan 10;38(11):e14940. doi: 10.1002/adma.202514940 (PMC12921339; doi:10.1002/adma.202514940)
Supplement: Supplementary file 1 — Supporting File: adma72077‐sup‐0001‐SuppMat.pdf. [file ADMA-38-e14940-s001.pdf]

Supporting Information

Peptide-Induced Ferroelectricity in Charge-Transfer Supramolecular Materials

James V. Passarelli[†1], *Yang Yang*[†2], *Cara S. Smith*[2,5], *Jing Hao*[3], *Dhwanit R. Dave*[2], *Ashwin Narayanan*[4], *Zaida Álvarez*[2,6], *Broderick K. Johnson*[1], *Kelly A. Marshall*[7], *Ivan Fithian*[1], *Hiroaki Sai*[2], *Ruomeng Qiu*[2], *Charlotte L. Stern*[1], *Liam C. Palmer*[1,2], *Evangelos Kiskinis*[2,8], *and Samuel I. Stupp*[*,1,2,4,5,8]

[1]Department of Chemistry, Northwestern University, Evanston, Illinois 60208, USA. [2]Center for Regenerative Nanomedicine, Northwestern University, 303 E Superior, Chicago, Illinois 60611. [3]Division of Electromagnetic Engineering, KTH Royal Institute of Technology, Stockholm, 100 44, Sweden. [4]Department of Materials Science and Engineering, Northwestern University, Evanston, Illinois 60208, USA. [5]Department of Biomedical Engineering, Northwestern University, Evanston, IL 60208, USA. [6]CIBER en Bioingeniería, Biomateriales y Nanomedicina, CIBER-BBN, Madrid 28029, Spain. [7]The Ken & Ruth Davee Department of Neurology, Feinberg School of Medicine, Northwestern University, Chicago, IL 60611, USA. [8]Department of Medicine, Northwestern University, Chicago, Illinois 60611, USA.

[†]These authors contributed equally to this work.

*Corresponding author: s-stupp@northwestern.edu

Table of Contents

List of Figures **3**

List of Table **4**

1 Organic molecule synthesis and molecular characterization **5**

1.1 Synthesis of donor-acceptor (DA) dyads 5

1.2 Synthesis of DA-Peptides 10

2 Crystal structures and CT interactions of CT complexes **21**

2.1 Crystal structures of the DA complexes 21

2.2 UV-vis absorption spectra of DA complexes and their precursors 23

2.3 UV-vis absorption spectra of DA-PA assemblies 25

2.4 Selected area electron diffraction (SAED) and wide-angle X-ray scattering (WAXS) of DA and DA-PA assemblies 30

3 Chiral peptide induced symmetry breaking in DA-PA crystals **35**

4 DFT geometry optimization and calculations **39**

5 Racemized DA-PA assemblies **41**

6 Electrical properties of DA-PA crystals **44**

6.1 Linear capacitance and resistance values of the DA-PA samples 44

6.2 Data processing of P-E loops 47

6.3 Morphologies and electrical properties of DA-PA samples 49

6.4 Electrical properties of racemized DA-PA samples 51

6.5 Low-field ferroelectric properties and piezoelectric responses of DA-PA samples 52

7 Neuron experiments **56**

7.1 Neuronal culture on DA-PA coatings 56

7.2 Whole-cell current-clamp experiments 60

Reference: **62**

List of Figures

Supplementary Figure 1. Photograph of DA1, DA2, DA3, and DA4 in the solid state. 21
Supplementary Figure 2. DA2 Single Crystal structure. 21
Supplementary Figure 3. DA3 Single Crystal structure. 22
Supplementary Figure 4. An image of a DA1 single crystal with face indexing. 22
Supplementary Figure 5. UV-Vis absorbance of DA2 and DA4 in water and DMF 23
Supplementary Figure 6. UV-Vis absorbance of DA1 in water and DMF................................. 24
Supplementary Figure 7. UV-Vis absorbance of DA2-PAs in water and DMF.......................... 25
Supplementary Figure 8. UV-Vis absorbance of DA3-PAs in water and DMF.......................... 26
Supplementary Figure 9. UV-Vis absorbance of DA4-PAs in water and DMF.......................... 27
Supplementary Figure 10. Quantitative dimensions of DA-PA assemblies. 28
Supplementary Figure 11. Proposed molecular packing for DA1 and DA1-VK crystal lattices. 29
Supplementary Figure 12. SAED and WAXS patterns of DA1 assemblies. 30
Supplementary Figure 13. WAXS pattern of DA1-VKVK assemblies...................................... 31
Supplementary Figure 14. SAED and WAXS patterns of DA2-PA assemblies. 32
Supplementary Figure 15. SAED and WAXS patterns of DA3-PA assemblies. 33
Supplementary Figure 16. SAED and WAXS patterns of DA4-PA assemblies. 34
Supplementary Figure 17. Circular dichroism (CD) and linear dichroism (LD) spectroscopy of DA1-PAs... 35
Supplementary Figure 18. Two-photon confocal microscopy of dried samples of DA1 and DA2 assemblies. 36
Supplementary Figure 19. DA1-K two-photon confocal microscopy reveals SHG activity........ 36
Supplementary Figure 20. CD spectroscopy and two-photon confocal microscopy of DA1-VKVK assemblies.. 37
Supplementary Figure 21. CD spectroscopy and two-photon confocal microscopy of DA2-PA assemblies. 38
Supplementary Figure 22. TEM, UV-Vis and WAXS of freshly dissolved DA2-VK samples... 38
Supplementary Figure 23. DFT optimized geometry and TD-DFT calculations of DA1 and DA1-VK monomers. 39
Supplementary Figure 24. DFT optimized geometry and TD-DFT calculations for a cluster of four DA1 molecules. 39
Supplementary Figure 25. DFT calculations for a cluster of four DA1-VK molecules. 40
Supplementary Figure 26. DFT optimized structures for clusters of DA1 and DA1-VK. 40
Supplementary Figure 27. AFM images of the racemized DA2-PA nanostructures................... 41
Supplementary Figure 28. CD absorption spectroscopy of the racemized DA2-PA nanostructures. 42
Supplementary Figure 29. Two-photon confocal microscopy of the racemized DA2-PA nanostructures. 43
Supplementary Figure 30. Complex capacitance of DA1-PA assemblies.................................. 44
Supplementary Figure 31. Complex capacitance of DA2-PA assemblies.................................. 45
Supplementary Figure 32. Complex capacitance of DA3-VK and DA4.................................... 46
Supplementary Figure 33. Leakage current of the samples under a DC voltage of 1.0 V. 46
Supplementary Figure 34. Data processing of P-E loops measured on DA2-VK assemblies...... 48

Supplementary Figure 35. TEM micrograph of DA1-VK assemblies. 49
Supplementary Figure 36. P-E loops of DA1-VKVK assemblies and DA2-VK prepared from unannealed solution. 49
Supplementary Figure 37. AFM morphology of the dry-state DA and DA-PA nanostructures on the testing substrate. 49
Supplementary Figure 38. The P-E loops of (a) DA1-K, (b) DA2-K, (c) DA3-K and (d) DA4-K. 50
Supplementary Figure 39. Resistance and linear capacitance of DA-K samples. 50
Supplementary Figure 40. The P-E loops of racemized DA-PA samples. 51
Supplementary Figure 41. Resistance and linear capacitance of racemized DA-PA samples. 51
Supplementary Figure 42. The experimental setup for measuring the piezoelectric response of DA-PA samples. 53
Supplementary Figure 43. Fast Fourier transform (FFT) analysis of the piezoelectric voltage waveforms in Figure 4k. 54
Supplementary Figure 44. P-E loops and morphologies of a different batch of ferroelectric DA-PA samples. 55
Supplementary Figure 45. Evaporation method for preparing DA-PA coated poly-D-lysine (PDL) glass coverslips. 56
Supplementary Figure 46. Scanning electron microscopy (SEM) images of DA-PA coated PDL glass coverslips prepared via the evaporation method after 48 hours of culture. 56
Supplementary Figure 47. SEM images of neurons cultured on DA-PA coatings after 48 hours. 57
Supplementary Figure 48. Representative fluorescent images of neurons cultured on DA-PA coatings after one week. 58
Supplementary Figure 49. Quantification of DAPI positive cells per field after a 48-hour culture. 58
Supplementary Figure 50. Quantifications of cell aggregates and DAPI positive cells after a one-week culture. 59
Supplementary Figure 51. Whole-cell current-clamp experiments. 60
Supplementary Figure 52. Average values of (a) resting membrane potential voltage, (b) cell capacitance, and (c) input resistance of primary cortical neurons cultured for 12-14 days on DA-PA coatings. 61
Supplementary Figure 53. Action potential properties of neurons cultured for 10-15 days on DA-PA coatings. 61

List of Table

Supplementary Table 1. The C_l and R_l values of the samples calculated from the frequency-domain impedance and leakage current measurements. 47
Supplementary Table 2. Comparison of P_r, E_c and P_r/E_c values among ferroelectric DA-PA assemblies and other metal-free room-temperature organic ferroelectrics. 52

1 Organic molecule synthesis and molecular characterization

1.1 Synthesis of donor-acceptor (DA) dyads

The donor-acceptor carboxylic acid dyads were synthesized through the coupling of the corresponding naphthalene-alkoxy-*n*-propyl amine and *tert*-butyl 4-aminobutanoate. The final products were formed by deprotection with trifluoracetic acid.

NMR Characterization: NMR spectra (^{1}H and ^{13}C) were collected on a 500 MHz Bruker Avance III HD system with a TXO Prodigy Probe. Deuterated solvents (dimethyl sulfoxide-*d*6 and chloroform-*d*) were purchased from Sigma–Aldrich and all chemical shifts are reported from spectra internally referenced to the solvent residual.

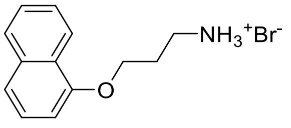

1-Naphthalene-*O*-propyl-NH$_3$Br (1): The corresponding *tert*-butyloxycarbonyl (BOC) protected amine was treated with hydrobromic acid to produce **1**.

^{1}H NMR (500 MHz, DMSO-*d*$_6$) δ 8.18 (ddd, *J* = 7.7, 2.0, 0.8 Hz, 1H), 7.93 – 7.85 (m, 1H), 7.81 (s, 3H), 7.57 – 7.46 (m, 3H), 7.43 (dd, *J* = 8.3, 7.5 Hz, 1H), 6.97 (dd, *J* = 7.6, 1.0 Hz, 1H), 4.25 (t, *J* = 5.9 Hz, 2H), 3.10 (q, *J* = 6.3 Hz, 2H), 2.16 (dq, *J* = 7.9, 6.1 Hz, 2H).

^{13}C NMR (126 MHz, DMSO-*d*$_6$) δ 153.12, 133.41, 126.90, 125.87, 125.59, 124.68, 124.25, 120.87, 119.48, 104.62, 64.17, 35.96, 26.34.

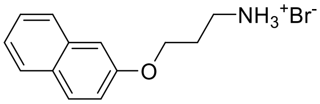

2-Naphthalene-*O*-propyl-NH$_3$Br (2): Using previously reported methods[1], 2-hydroxynaphthalene was substituted to produce **2**.

^{1}H NMR (500 MHz, DMSO-*d*$_6$) δ 7.87 – 7.77 (m, 3H), 7.76 – 7.72 (m, 3H), 7.47 (ddd, *J* = 8.2, 6.8, 1.3 Hz, 1H), 7.39 – 7.30 (m, 2H), 7.18 (dd, *J* = 8.9, 2.6 Hz, 1H), 4.19 (t, *J* = 6.1 Hz, 2H), 3.02 (dd, *J* = 7.9, 6.9 Hz, 2H), 2.12 – 2.03 (m, 2H).

^{13}C NMR (126 MHz, DMSO-*d*$_6$) δ 156.64, 134.67, 129.81, 129.01, 128.02, 127.11, 126.96, 124.15, 119.11, 107.27, 65.09, 36.94, 27.30.

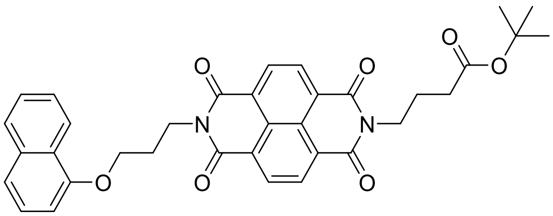

DA1-OtBu (3): To a 50 mL round bottom flask (RBF), was added 1.00 g (3.54 mmol) of **1**, 0.623 g (3.54 mmol) of 2-methyl-2-propanyl 4-aminobutanoate, and 20 mL of dimethylformamide (DMF). To this flask was added 0.756 g (7.40 mmol) of triethylamine. The resulting mixture was stirred at room temperature until all solids dissolved. To this was added 0.950 g (3.54 mmol) of naphthalenetetracarboxylic dianhydride followed by 10 mL of DMF to transfer solids from the sides of the flasks into the reaction mixture. The RBF was then transferred to an oil bath set to 100 °C and stirred for 2 hours. The resulting solution was allowed to cool and then concentrated under reduced pressure by rotary evaporation to approximately 10 mL of solvent. This solution was then added dropwise to 500 mL of water. The resulting orange precipitate was recovered by vacuum filtration. This solid was allowed to dry on the frit overnight. The resulting orange solid was dissolved in dichloromethane and loaded onto silica gel. Pure product **3** was obtained after column chromatography with 0.1% methanol (MeOH) in dichloromethane (DCM) switching to 0.5% MeOH in DCM after the first impurity band eluded.

^{1}H NMR (500 MHz, $CDCl_3$) δ 8.64 (q, J = 7.6 Hz, 4H), 7.91 – 7.85 (m, 1H), 7.61 (dt, J = 8.2, 1.0 Hz, 1H), 7.36 – 7.29 (m, 2H), 7.26 – 7.25 (m, 1H), 7.00 (ddd, J = 8.2, 6.8, 1.3 Hz, 1H), 6.76 (dd, J = 6.0, 2.6 Hz, 1H), 4.56 (t, J = 6.9 Hz, 2H), 4.34 (t, J = 5.6 Hz, 2H), 4.29 – 4.23 (m, 2H), 2.49 – 2.41 (m, 2H), 2.39 (t, J = 7.4 Hz, 2H), 2.07 (p, J = 7.4 Hz, 2H), 1.43 (s, 10H).

^{13}C NMR (126 MHz, $CDCl_3$) δ 172.05, 163.06, 162.85, 154.22, 134.18, 130.81 (d, J = 1.7 Hz), 127.17, 126.66, 126.54, 126.52, 126.43, 125.93, 125.85, 125.17, 124.64, 121.45, 120.16, 104.47, 80.50, 66.31, 40.14, 38.90, 33.13, 28.08, 27.87, 23.49.

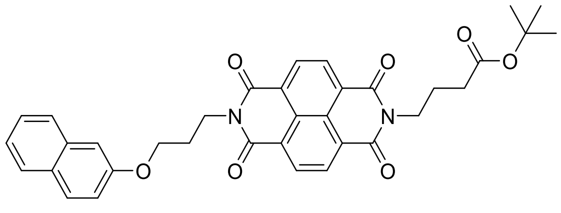

DA2-OtBu (4): Using the same method as compound **3**, compound **4** was prepared. In this reaction, 1.8 g (6.4 mmol) of **2**, 1.24 g (6.40 mmol) of 2-methyl-2-propanyl 4-aminobutanoate, 1.46 g (14.5 mmol) of TEA, and 1.55 g (5.80 mmol) of naphthalenetetracarboxylic dianhydride were utilized.

^{1}H NMR (500 MHz, $CDCl_3$) δ 8.73 (d, J = 0.9 Hz, 4H), 7.70 (dd, J = 8.1, 1.2 Hz, 1H), 7.65 (dd, J = 8.3, 1.1 Hz, 1H), 7.59 (d, J = 8.9 Hz, 1H), 7.40 (ddd, J = 8.2, 6.8, 1.3 Hz, 1H), 7.30 (ddd, J = 8.1, 6.8, 1.2 Hz, 1H), 7.05 (d, J = 2.5 Hz, 1H), 6.91 (dd, J = 8.9, 2.5 Hz, 1H), 4.49 (t, J = 7.0 Hz, 2H), 4.30 – 4.22 (m, 4H), 2.37 (dt, J = 17.2, 6.9 Hz, 4H), 2.07 (p, J = 7.3 Hz, 2H), 1.42 (s, 9H).

^{13}C NMR (126 MHz, $CDCl_3$) δ 172z.06, 162.96, 162.86, 156.61, 134.44, 130.99, 130.97, 129.23, 128.91, 127.57, 126.69 (d, J = 1.3 Hz), 126.60, 126.58, 126.31, 123.61, 118.67, 106.67, 80.48, 66.07, 40.22, 38.62, 33.15, 28.06, 27.87, 23.45.

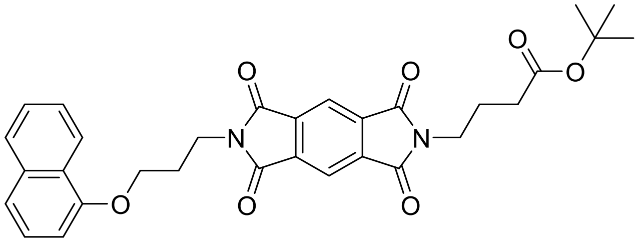

DA3-OtBu (5): Using the same method as compound **3**, compound **5** was prepared. In this application, 2.25 g (7.97 mmol) of **1**, 1.56 g (7.97 mmol) of 2-methyl-2-propanyl 4-aminobutanoate, 1.69 g (16.7 mmol) of TEA, and 1.74 g (7.97 mmol) of pyromellitic dianhydride were utilized.

^{1}H NMR (500 MHz, $CDCl_3$) δ 8.09 (s, 2H), 8.01 (dq, *J* = 9.0, 0.8 Hz, 1H), 7.71 – 7.65 (m, 1H), 7.39 – 7.27 (m, 4H), 6.75 (dd, *J* = 7.2, 1.5 Hz, 1H), 4.27 (t, *J* = 5.6 Hz, 2H), 4.08 (t, *J* = 6.8 Hz, 2H), 3.78 (t, *J* = 6.9 Hz, 2H), 2.43 – 2.34 (m, 2H), 2.31 (t, *J* = 7.3 Hz, 2H), 2.00 (p, *J* = 7.1 Hz, 2H), 1.43 (s, 9H).

^{13}C NMR (126 MHz, $CDCl_3$) δ 171.73, 166.32, 166.14, 154.06, 137.12, 136.99, 134.34, 127.33, 126.20, 125.85, 125.24, 124.98, 121.72, 117.99, 104.60, 80.72, 65.83, 37.97, 36.65, 32.78, 28.06, 28.02, 23.77.

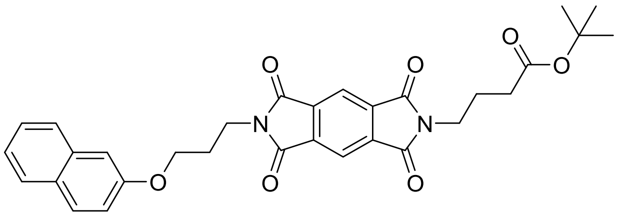

DA4-OtBu (6): Using the same method as compound **3**, compound **5** was prepared. In this application, 1.5 g (5.3 mmol) of **2**, 1.04 g (5.31 mmol) of 2-methyl-2-propanyl 4-aminobutanoate, 1.12 g (11.2 mmol) of TEA, and 1.16 g (5.31 mmol) of pyromellitic dianhydride were utilized.

^{1}H NMR (500 MHz, $CDCl_3$) δ 8.24 (s, 1H), 7.73 (dd, *J* = 8.1, 1.1 Hz, 1H), 7.70 – 7.63 (m, 1H), 7.41 (ddd, *J* = 8.2, 6.8, 1.3 Hz, 1H), 7.31 (ddd, *J* = 8.1, 6.8, 1.2 Hz, 0H), 7.06 (d, *J* = 2.6 Hz, 1H), 6.96 (dd, *J* = 8.9, 2.5 Hz, 1H), 4.17 (t, *J* = 5.8 Hz, 1H), 4.02 (t, *J* = 6.9 Hz, 1H), 3.80 (t, *J* = 6.9 Hz, 1H), 2.34 – 2.25 (m, 2H), 2.00 (p, *J* = 7.1 Hz, 1H), 1.42 (s, 5H).

^{13}C NMR (126 MHz, $CDCl_3$) δ 171.75, 166.25, 166.20, 156.50, 137.23 (d, *J* = 1.5 Hz), 134.44, 129.44, 129.02, 127.63, 126.73, 126.39, 123.71, 118.59, 118.21, 106.73, 80.71, 65.59, 38.06, 36.39, 32.80, 28.05, 23.73.

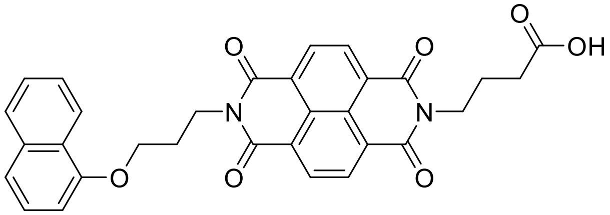

DA1 (7): To compound **3**, was added 10 mL of DCM and 10 mL of trifluoroacetic acid (TFA). The resulting solution was stirred for 30 min. Following this, the solvent was removed by rotary evaporation. The resulting product was used without further purification.

^{1}H NMR (500 MHz, DMSO-d_6) δ 12.05 (s, 1H), 8.62 (d, J = 0.9 Hz, 4H), 7.93 – 7.87 (m, 1H), 7.75 (dt, J = 8.2, 1.0 Hz, 1H), 7.43 – 7.33 (m, 3H), 7.14 (ddd, J = 8.2, 6.8, 1.2 Hz, 1H), 6.91 (dd, J = 6.6, 2.1 Hz, 1H), 4.40 (t, J = 6.9 Hz, 2H), 4.30 (t, J = 5.7 Hz, 2H), 4.12 (t, J = 7.0 Hz, 2H), 2.33 (dt, J = 15.8, 6.8 Hz, 4H), 1.93 (p, J = 7.2 Hz, 2H).

^{13}C NMR (126 MHz, DMSO-d_6) δ 174.47, 163.31, 163.25, 154.29, 134.28, 130.78, 127.71, 126.88 – 126.71 (m), 126.65, 126.57, 121.51, 120.21, 66.66, 38.58, 31.74, 27.80, 23.37.

Meas. m/z	Sum Formula	m/z	Ion Formula	err [ppm]	rdb	Adduct
535.150614	C31H24N2O7	535.151075	C31H23N2O7	0.9	21	M-H

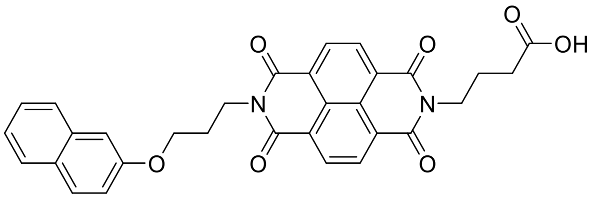

DA2 (8): To compound **3**, was added 10 mL of DCM and 10 mL of trifluoroacetic acid (TFA). The resulting solution was stirred for 30 min. Following this, the solvent was removed by rotary evaporation. The resulting product was used without further purification.

^{1}H NMR (500 MHz, DMSO-d_6) δ 12.03 (s, 1H), 8.66 (s, 3H), 7.81 – 7.76 (m, 1H), 7.76 – 7.70 (m, 2H), 7.42 (ddd, J = 8.3, 6.8, 1.3 Hz, 1H), 7.31 (ddd, J = 8.1, 6.8, 1.2 Hz, 1H), 7.23 (d, J = 2.6 Hz, 1H), 4.32 (t, J = 6.9 Hz, 2H), 4.23 (t, J = 6.0 Hz, 2H), 4.12 (t, J = 6.9 Hz, 2H), 2.35 (t, J = 7.3 Hz, 2H), 2.23 (p, J = 6.5 Hz, 2H), 1.93 (p, J = 7.2 Hz, 2H).

^{13}C NMR (126 MHz, DMSO-d_6) δ 174.49, 163.31, 156.79, 134.66, 130.85, 130.81, 129.63, 128.87, 127.93, 127.07, 126.91, 126.84, 126.79, 126.73, 126.67, 123.97, 118.97, 107.21, 66.44, 38.38, 31.76, 27.69, 23.36.

Meas. m/z	Sum Formula	m/z	Ion Formula	err [ppm]	rdb	Adduct
535.150331	C31H24N2O7	535.151075	C31H23N2O7	1.4	21	M-H

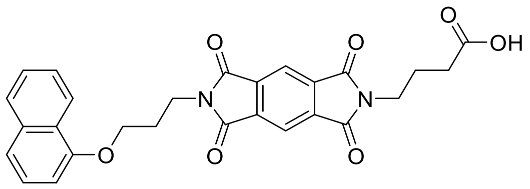

DA3 (9): To compound **3**, was added 10 mL of DCM and 10 mL of trifluoroacetic acid (TFA). The resulting solution was stirred for 30 min. Following this, the solvent was removed by rotary evaporation. The resulting product was used without further purification.

^{1}H NMR (500 MHz, DMSO) δ 12.09 (s, 1H), 8.09 (s, 2H), 7.98 (dp, *J* = 8.4, 0.7 Hz, 1H), 7.80 (dt, *J* = 8.2, 0.9 Hz, 1H), 7.48 – 7.28 (m, 4H), 6.90 (dd, *J* = 7.4, 1.3 Hz, 1H), 4.24 (t, *J* = 5.7 Hz, 2H), 3.93 (t, *J* = 6.7 Hz, 2H), 3.66 (t, *J* = 6.8 Hz, 2H), 2.34 – 2.21 (m, 4H), 1.85 (p, *J* = 7.0 Hz, 2H).

^{13}C NMR (126 MHz, DMSO) δ 174.31, 166.87, 166.82, 154.23, 137.43, 137.34, 134.39, 127.82, 126.69, 121.71, 120.32, 117.49, 105.51, 66.16, 37.90, 36.28, 31.38, 27.96, 23.67.

Meas. m/z	Sum Formula	m/z	Ion Formula	err [ppm]	rdb	Adduct
485.134899	C27H22N2O7	485.135425	C27H21N2O7	1.1	18	M-H

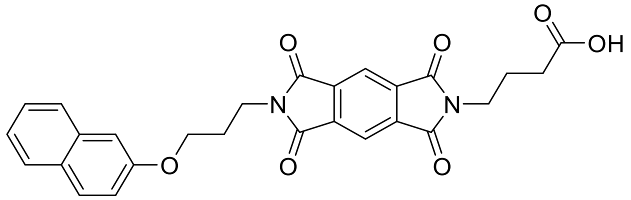

DA4 (10): To compound **3**, was added 10 mL of DCM and 10 mL of trifluoroacetic acid (TFA). The resulting solution was stirred for 30 min. Following this, the solvent was removed by rotary evaporation. The resulting product was used without further purification.

^{1}H NMR (500 MHz, DMSO-d_6) δ 12.08 (s, 1H), 8.16 (s, 2H), 7.82 – 7.68 (m, 3H), 7.43 (ddd, *J* = 8.3, 6.8, 1.3 Hz, 1H), 7.32 (ddd, *J* = 8.1, 6.8, 1.2 Hz, 1H), 7.22 (d, *J* = 2.5 Hz, 1H), 6.98 (dd, *J* = 8.9, 2.5 Hz, 1H), 4.17 (t, *J* = 5.9 Hz, 2H), 3.87 (t, *J* = 6.7 Hz, 2H), 3.67 (t, *J* = 6.8 Hz, 2H), 2.50 (p, *J* = 1.9 Hz, 8H), 2.31 (t, *J* = 7.2 Hz, 2H), 2.17 (p, *J* = 6.4 Hz, 2H), 1.86 (p, *J* = 7.0 Hz, 2H).

^{13}C NMR (126 MHz, DMSO) δ 174.33, 166.89 (d, *J* = 2.0 Hz), 156.74, 137.49, 137.44, 134.65, 129.71, 128.90, 127.95, 127.09, 126.82, 124.01, 118.95, 117.55, 107.21, 66.00, 37.92, 36.14, 31.40, 27.84, 23.66.

Meas. m/z	Sum Formula	m/z	Ion Formula	err [ppm]	rdb	Adduct
485.135079	C27H22N2O7	485.135425	C27H21N2O7	0.7	18	M-H

1.2 Synthesis of DA-Peptides

PA molecules were synthesized using standard Fmoc-solid-phase peptide chemistry using a Rink amide resin (100-200 mesh, Millipore Sigma). Fmoc deprotection was performed using 20% 4-methylpiperidine in DMF for 20 min. The resin was then washed with DMF and swollen with DCM. Amino acid couplings were performed with 4 equiv. protected amino acid, 4 equiv. hexafluorophosphate benzotriazole tetramethyl uronium (HBTU), and 6 equiv. DIEA in 50% DMF/50% for 1 hour. Coupling of DA tails were performed with 1.1 equiv. of DA-OH, 1.05 equiv. HBTU, and 2 equiv. DIEA in 50% DMF/50% overnight. DA-OH did not initially dissolve completely resulting in a cloudy suspension. After overnight reaction, the supernatant above resin beads was clear and resin had taken color of DA-OH. The crude DA-PA was cleaved from the resin using 19 mL of trifluoracetic acid, 0.2 mL of water, 0.2 mL of triisopropyl silane, and 0.6 mL of DCM. This cleavage solution was added to the resin and incubated with shaking for 2 hours. The resultant supernatant was collected and reduced in volume followed by precipitation in diethyl ether. The resulting solid was recovered by centrifugation. Purification was performed using a Teledyne Isco CombiFlash fitted with a reverse phase C18 column. Mass spectra acquired in acetonitrile/water (50:50 vol.%) mixture eluent are presented below. All peptides were amide terminated and subsequently ion-exchanged with HCl before use.

DA1-K:

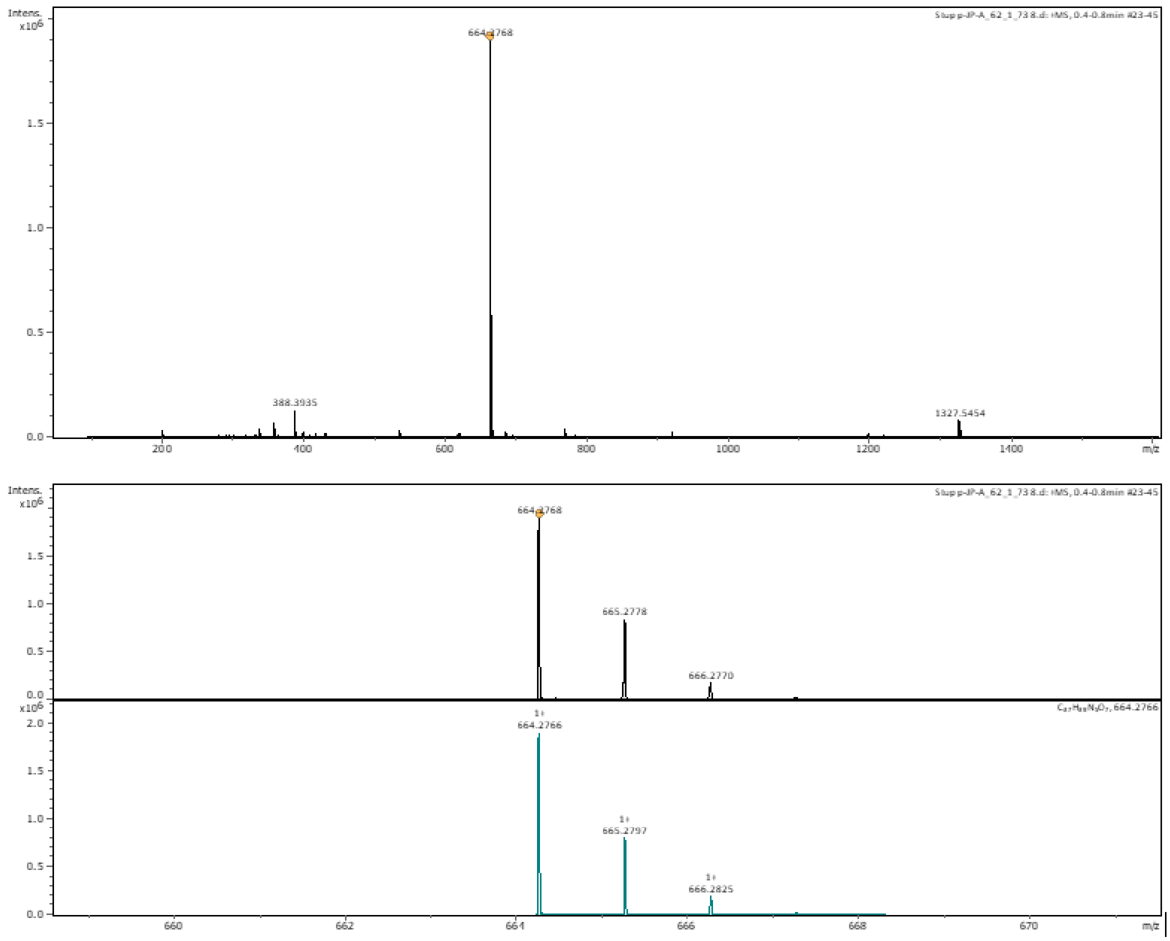

Meas. m/z	Sum Formula	m/z	Ion Formula	err [ppm]	rdb	Adduct
664.276752	C37H37N5O7	664.276575	C37H38N5O7	-0.3	22	M+H

DA1-VK:

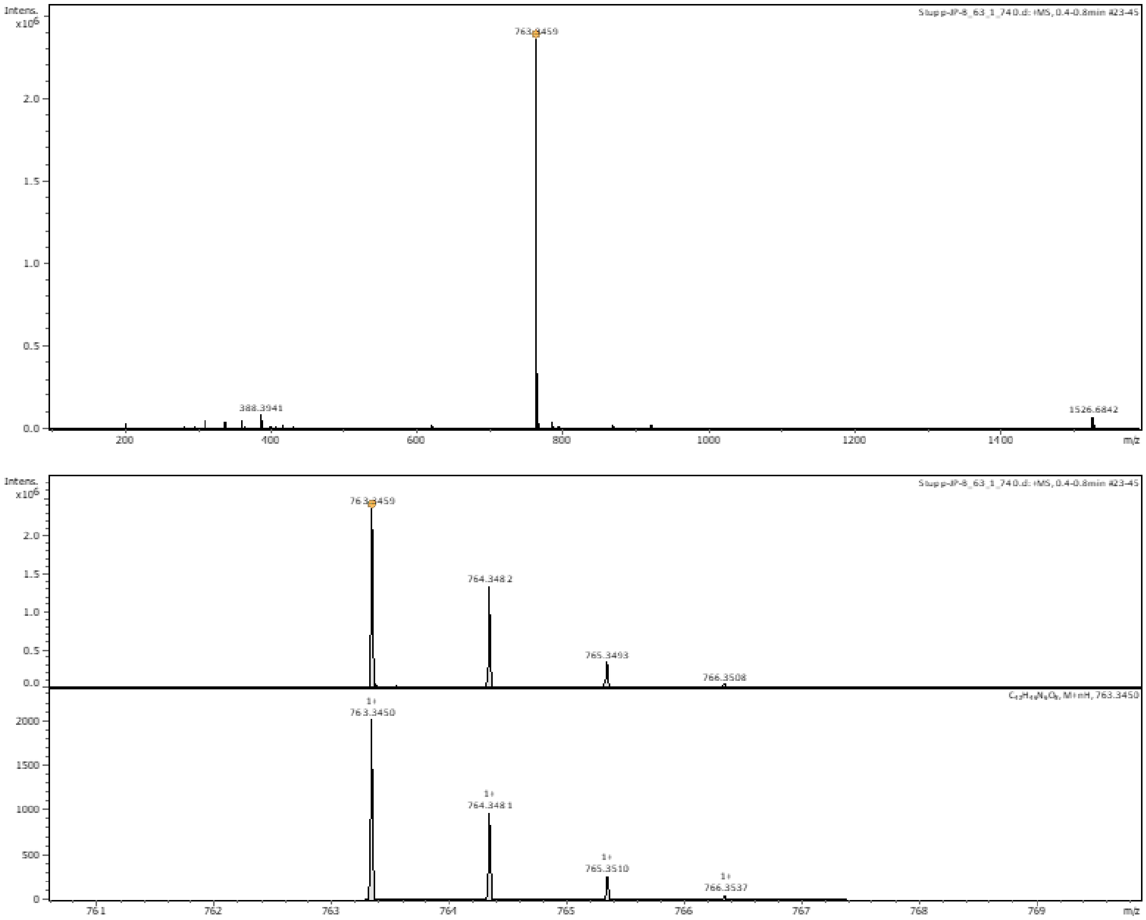

Meas. m/z	Sum Formula	m/z	Ion Formula	err [ppm]	rdb	Adduct
763.345915	C42H46N6O8	763.344989	C42H47N6O8	-1.2	23	M+H

DA1-VKVK:

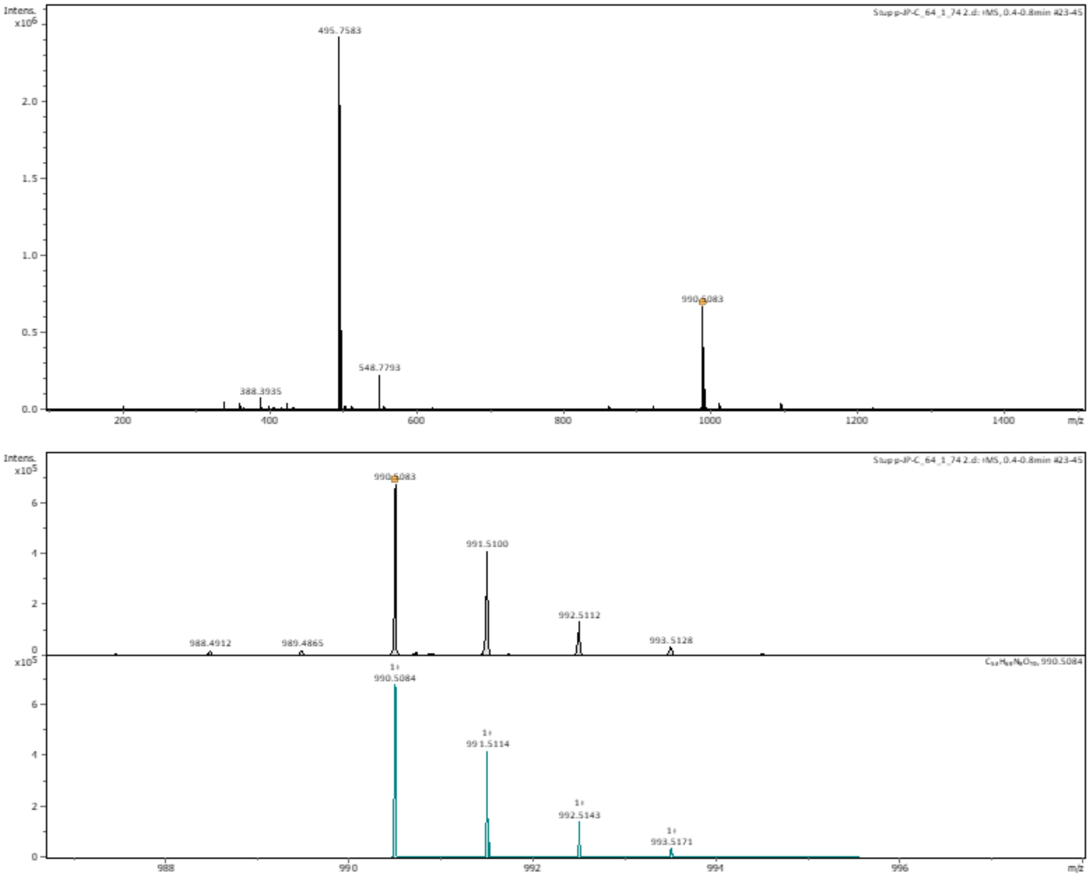

Meas. m/z	Sum Formula	m/z	Ion Formula	err [ppm]	rdb	Adduct
495.758337	C53H67N9O10	495.757821	C53H69N9O10	-1.0	25	M+H
990.508265	C53H67N9O10	990.508366	C53H68N9O10	0.1	25	M+H

DA2-K:

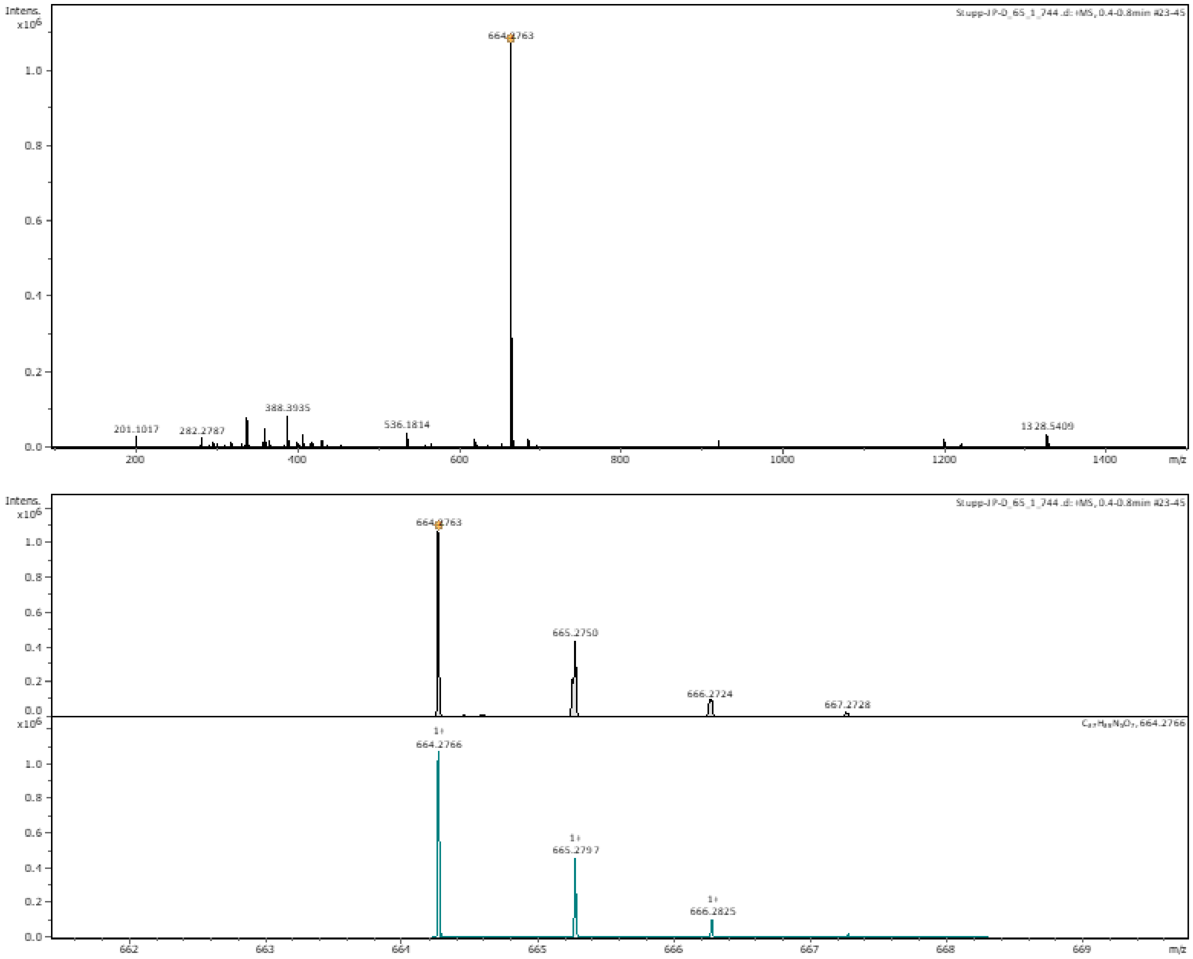

Meas. m/z	Sum Formula	m/z	Ion Formula	err [ppm]	rdb	Adduct
664.276292	C37H37N5O7	664.276575	C37H38N5O7	0.4	22	M+H

DA2-VK:

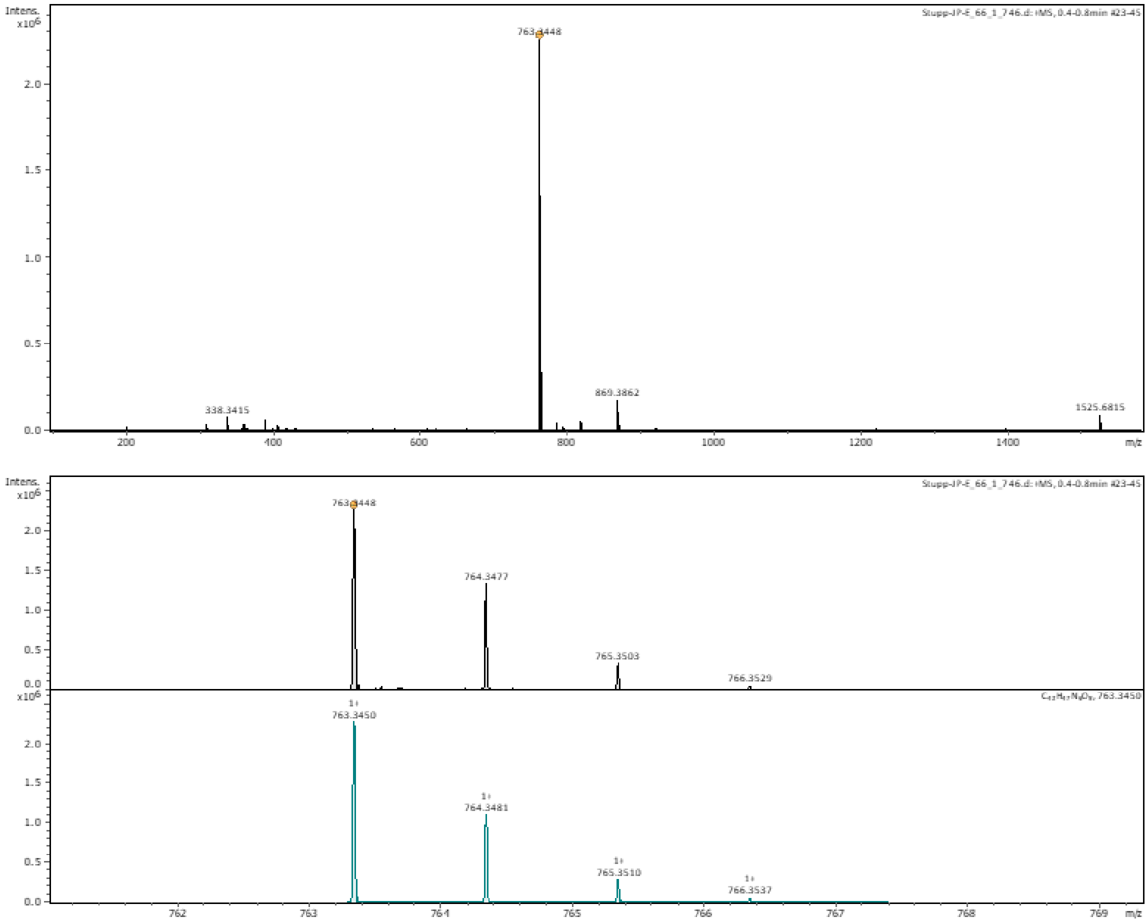

Meas. m/z	Sum Formula	m/z	Ion Formula	err [ppm]	rdb	Adduct
763.344845	C42H46N6O8	763.344989	C42H47N6O8	0.2	23	M+H

DA3-K:

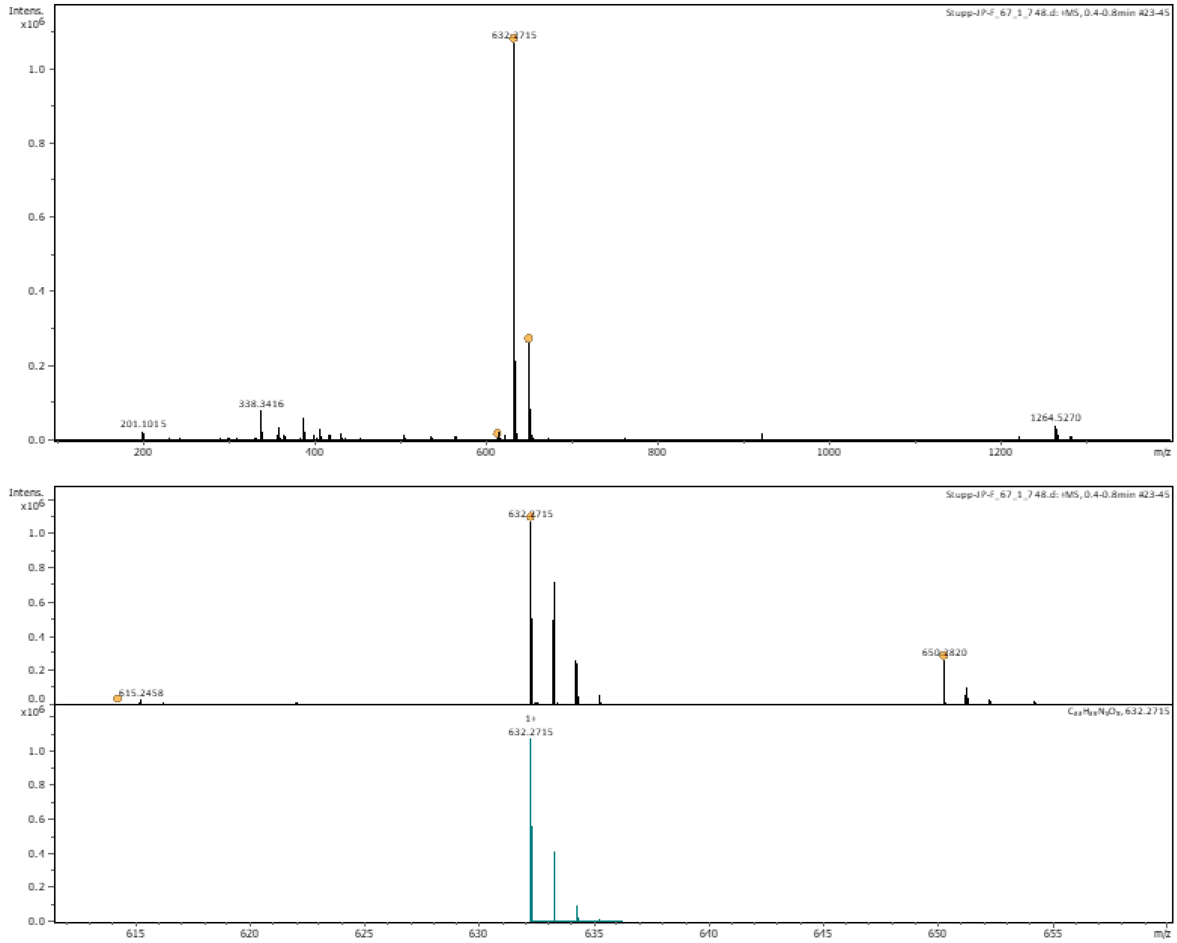

Meas. m/z	Sum Formula	m/z	Ion Formula	err [ppm]	rdb	Adduct
614.260935	C33H35N5O7	614.260925	C33H36N5O7	0	19	M+H
632.2715	C33H37N5O8	632.27149	C33H38N5O8	0	18	M+H
650.281992	C33H39N5O9	650.282054	C33H40N5O9	0.1	17	M+H
650.281992	C33H36N4O9	650.282054	C33H40N5O9	0.1	17	M+NH4

DA3-VK:

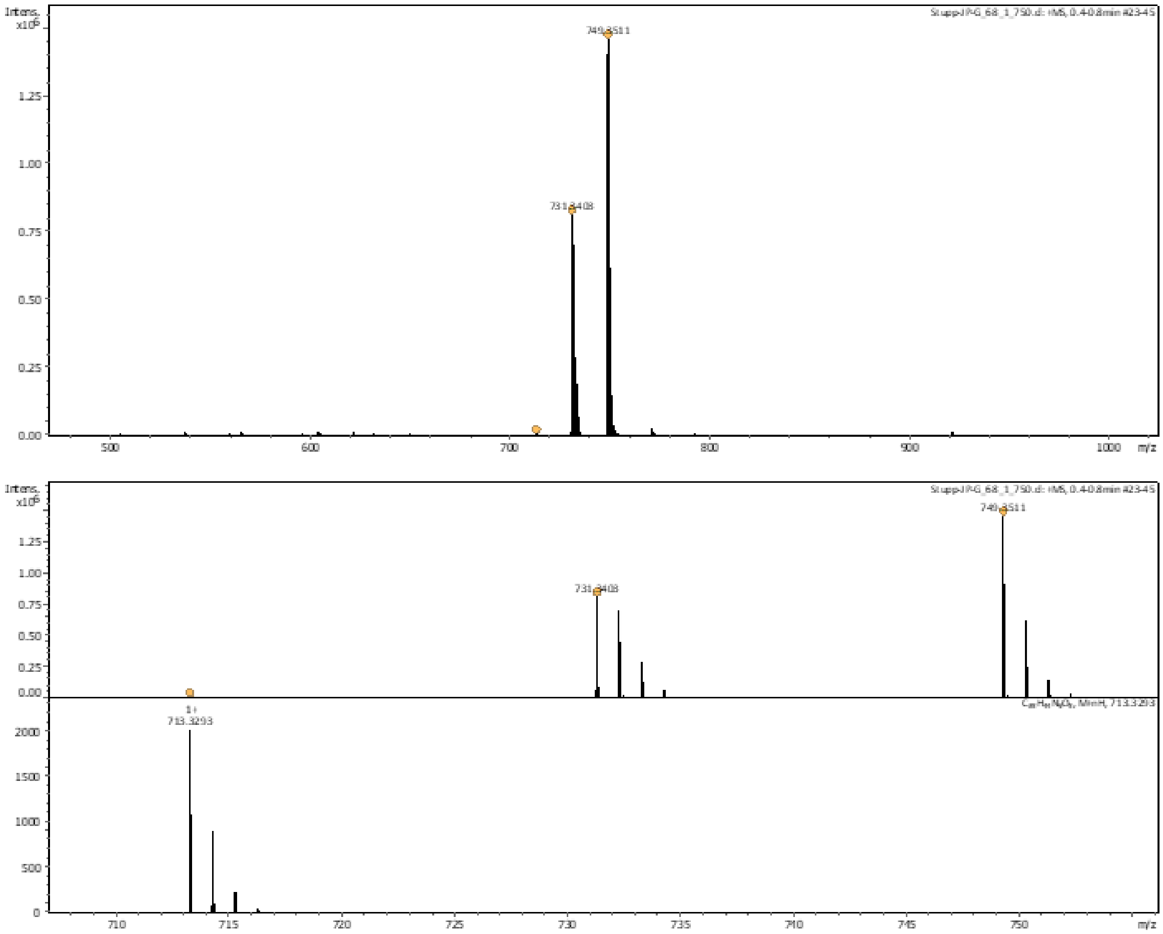

Meas. m/z	Sum Formula	m/z	Ion Formula	err [ppm]	rdb	Adduct
713.329998	C38H44N6O8	713.329339	C38H45N6O8	-0.9	20	M+H
713.329998	C38H41N5O8	713.329339	C38H45N6O8	-0.9	20	M+NH4
731.340341	C38H46N6O9	731.339904	C38H47N6O9	-0.6	19	M+H
731.340341	C38H43N5O9	731.339904	C38H47N6O9	-0.6	19	M+NH4
749.351091	C38H48N6O10	749.350468	C38H49N6O10	-0.8	18	M+H
749.351091	C38H45N5O10	749.350468	C38H49N6O10	-0.8	18	M+NH4
749.351091	C39H41N9O6	749.351806	C39H45N10O6	1	23	M+NH4

DA4-K:

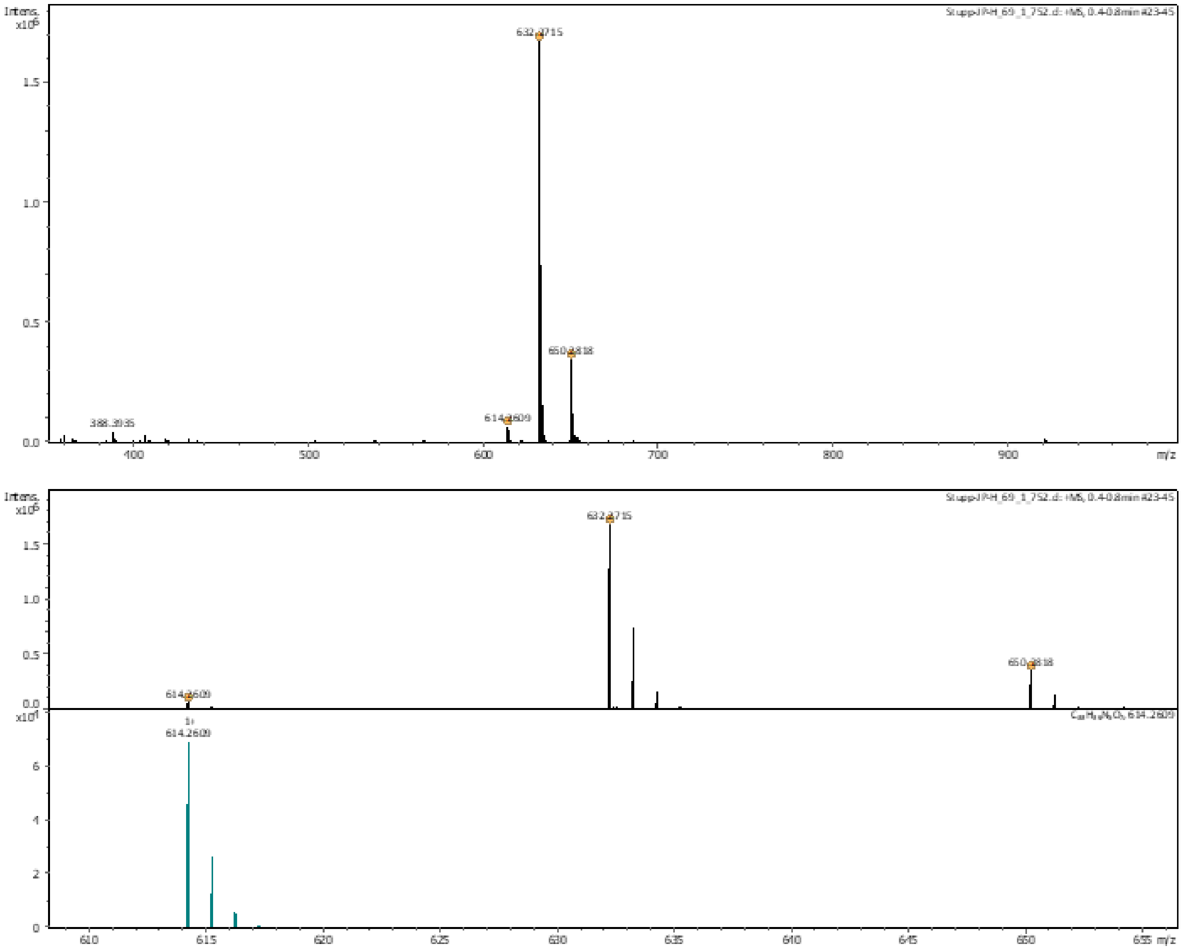

Meas. m/z	Sum Formula	m/z	Ion Formula	err [ppm]	rdb	Adduct
614.260902	C33H35N5O7	614.260925	C33H36N5O7	0	19	M+H
632.271531	C33H37N5O8	632.27149	C33H38N5O8	-0.1	18	M+H
650.281804	C33H39N5O9	650.282054	C33H40N5O9	0.4	17	M+H

DA4-VK:

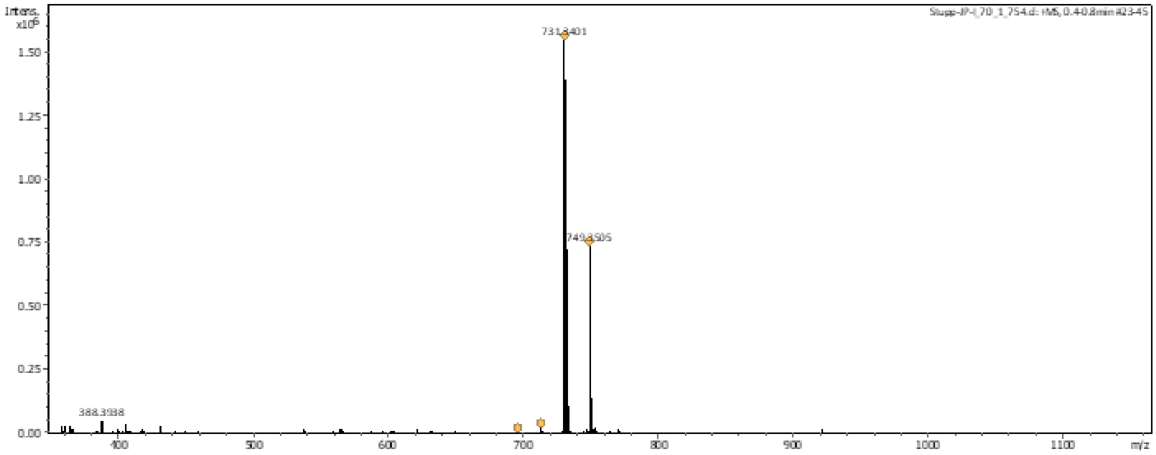

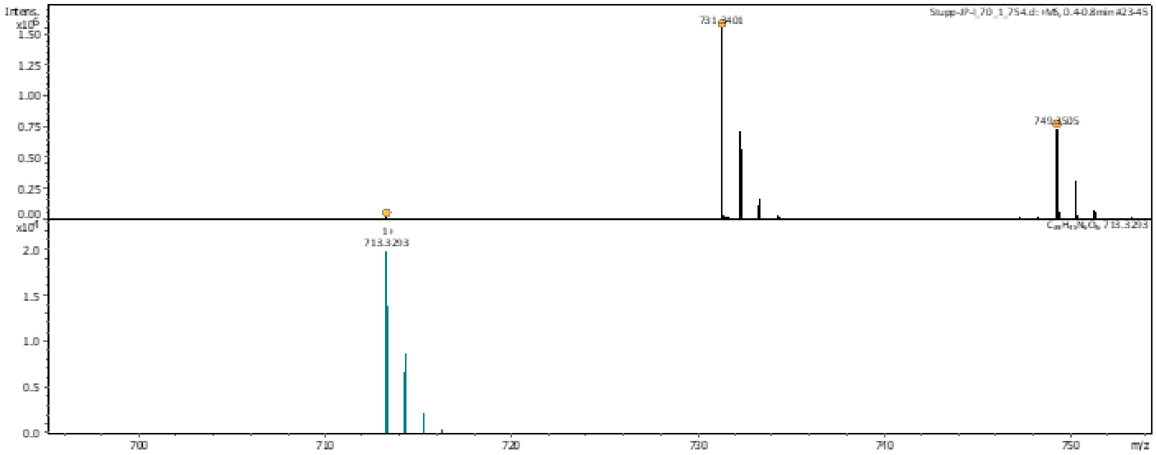

Meas. m/z	Sum Formula	m/z	Ion Formula	err [ppm]	rdb	Adduct
696.302453	C38H41N5O8	696.30279	C38H42N5O8	0.5	21	M+H
713.32954	C38H44N6O8	713.329339	C38H45N6O8	-0.3	20	M+H
713.32954	C38H41N5O8	713.329339	C38H45N6O8	-0.3	20	M+NH4
731.340119	C38H46N6O9	731.339904	C38H47N6O9	-0.3	19	M+H
749.350513	C38H48N6O10	749.350468	C38H49N6O10	-0.1	18	M+H

DA2-(D)V-K:

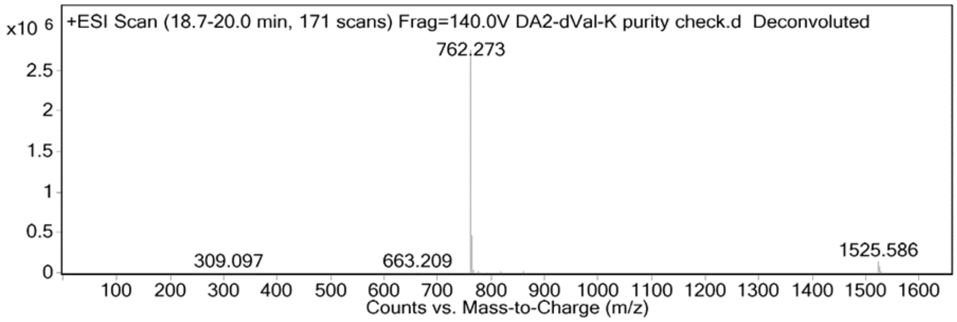

MS-ESI (m/z): [M] calc. for $C_{42}H_{45}N_6O_8$ 762.34, found 762.27.

DA2-V-(D)K:

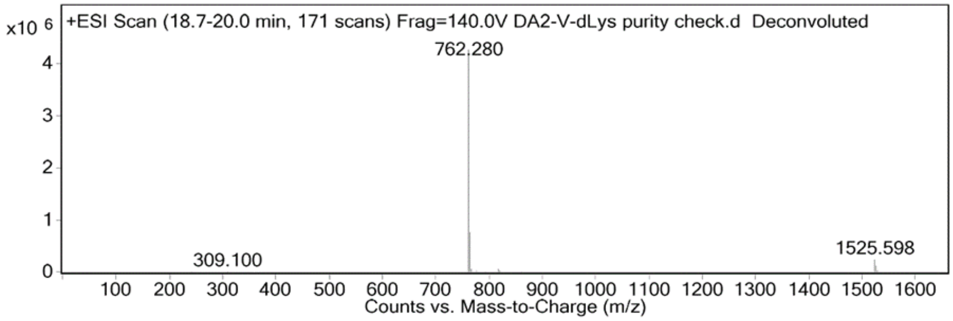

MS-ESI (m/z): [M] calc. for $C_{42}H_{45}N_6O_8$ 762.34, found 762.28.

DA2-(D)V-(D)K:

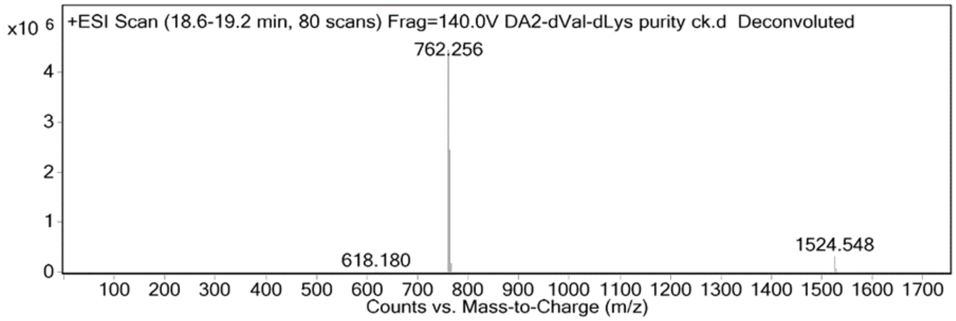

MS-ESI (m/z): [M] calc. for $C_{42}H_{45}N_6O_8$ 762.34, found 762.26.

2 Crystal structures and CT interactions of CT complexes

2.1 Crystal structures of the DA complexes

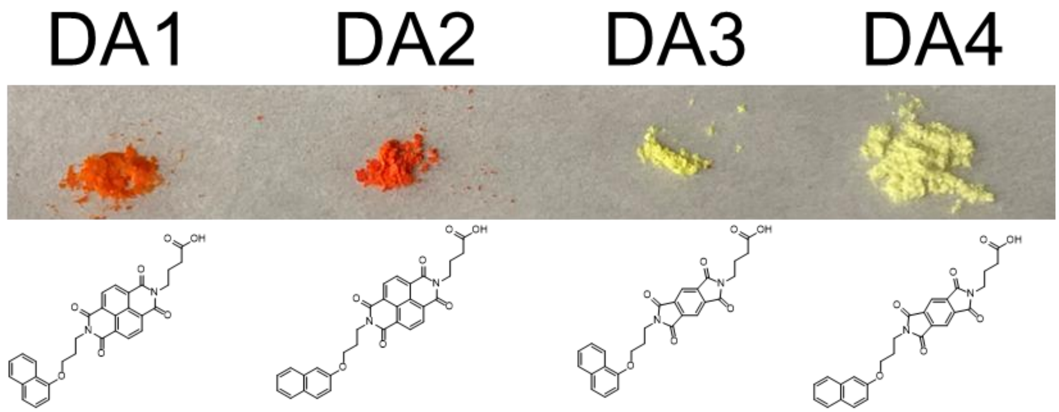

Supplementary Figure 1. Photograph of DA1, DA2, DA3, and DA4 in the solid state.

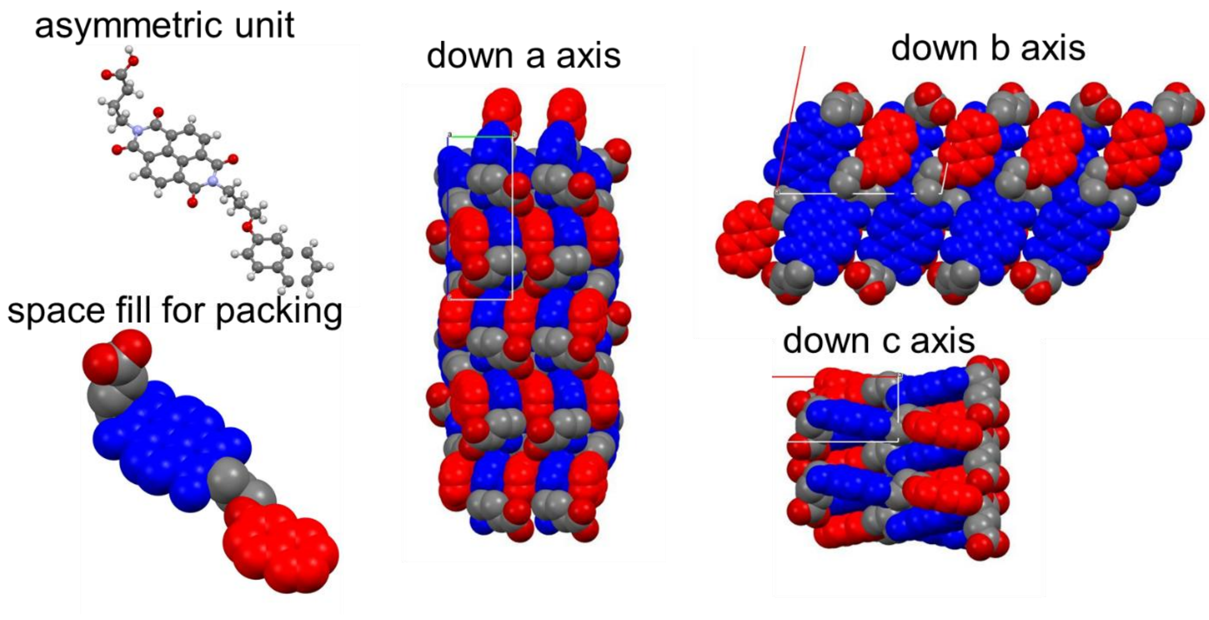

Supplementary Figure 2. DA2 Single Crystal structure.

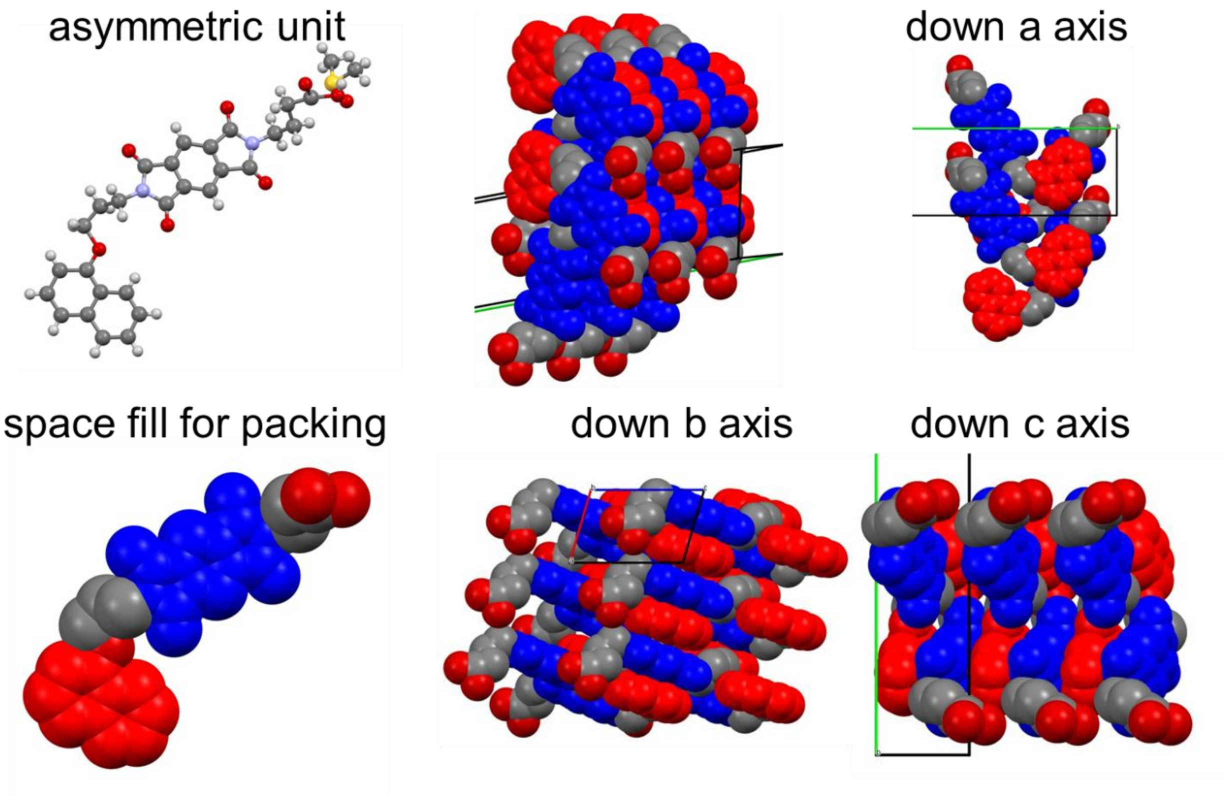

Supplementary Figure 3. DA3 Single Crystal structure.

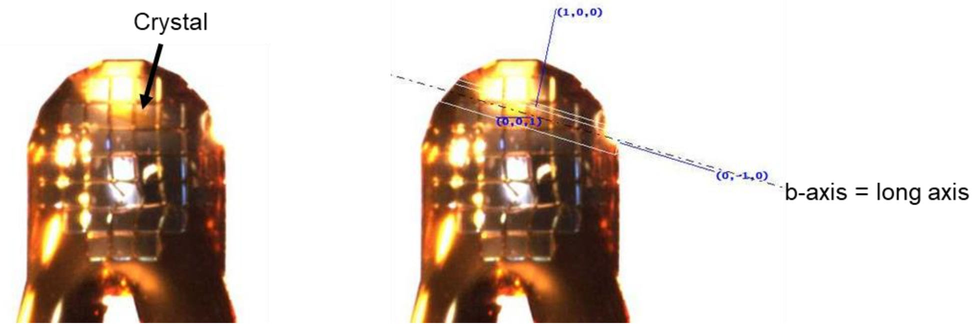

Supplementary Figure 4. An image of a DA1 single crystal with face indexing.

2.2 UV-vis absorption spectra of DA complexes and their precursors

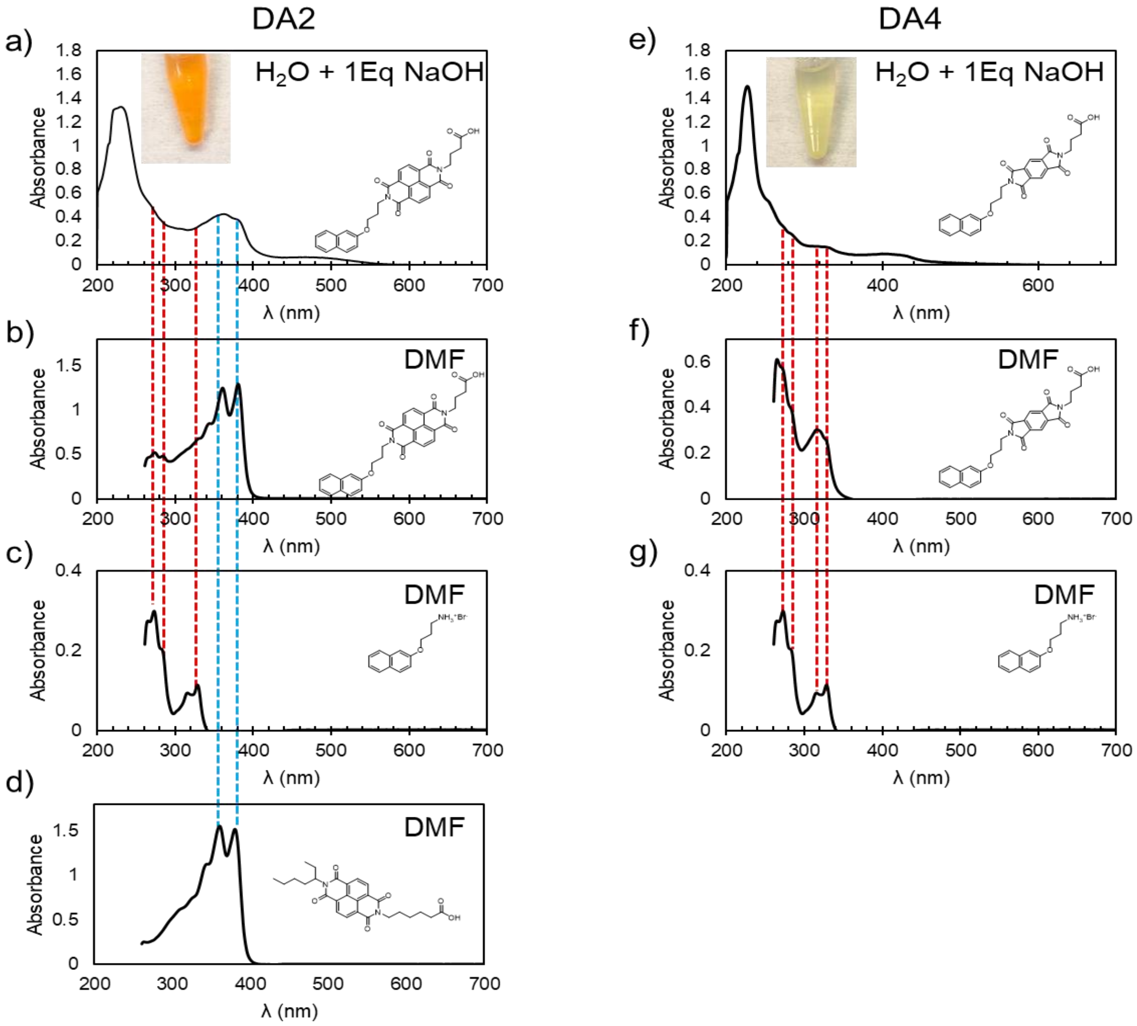

Supplementary Figure 5. UV-Vis absorbance of DA2 and DA4 in water and DMF. Path length equals 2 mm for all samples. (a) UV-Vis absorbance of **DA2** dissolved in water at 1 mM with addition of 1 equiv. sodium hydroxide and annealed. Sample diluted to 0.2 mM before measurement. (b) UV-Vis absorbance of **DA2** in DMF at 0.25 mM. (c) Naphthalene precursor for **DA2** in DMF at 0.5 mM. (d) Naphthalene diimide in DMF at 0.5 mM. Blue dashed lines denote acceptor absorbance features and red lines donor absorbance features. (e) UV-Vis absorbance of **DA4** dissolved in water at 1 mM with addition of 1 equiv. sodium hydroxide aged for 24 hours without annealing. Sample diluted to 0.2 mM before measurement. (f) UV-Vis absorbance of **DA4** in DMF at 0.25 mM. (c) naphthalene precursor for **DA2** in DMF at 0.5 mM. Red lines denote donor absorbance features.

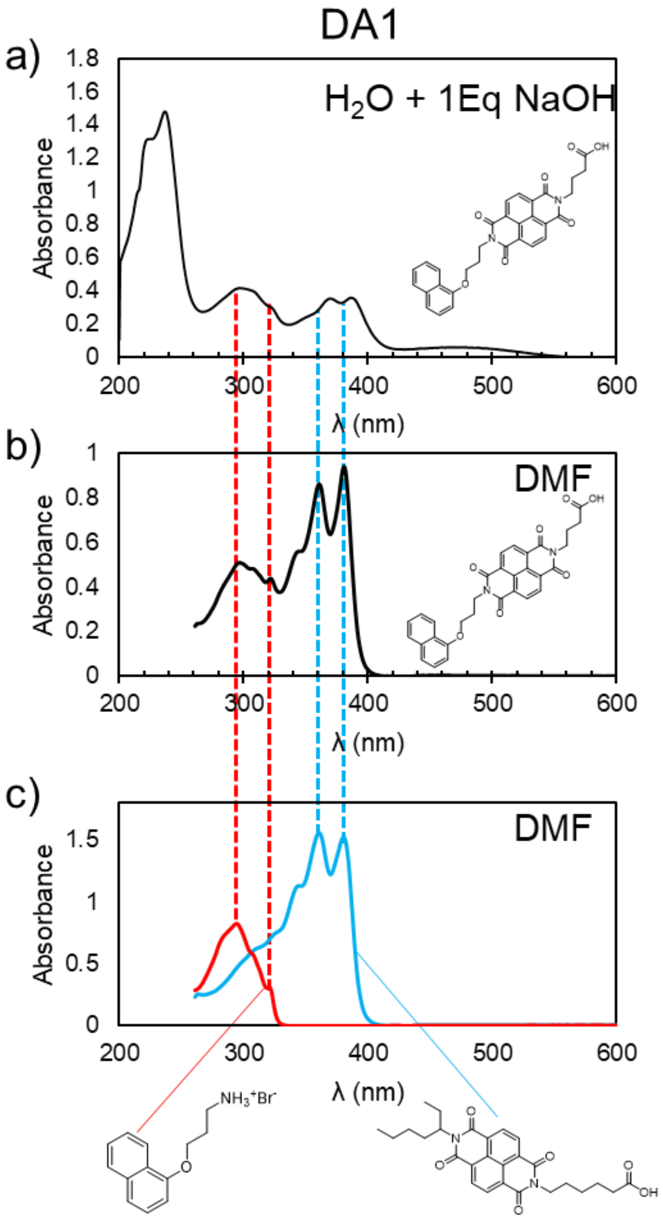

Supplementary Figure 6. UV-Vis absorbance of DA1 in water and DMF. (a) UV-Vis absorbance of **DA1** dissolved in water at 1 mM with addition of 1 equiv. sodium hydroxide and annealed. Sample was diluted to 0.2 mM before measurement. (b) UV-Vis absorbance of **DA1** in DMF at 0.25 mM. (c) Naphthalene precursor for **DA1** in DMF at 0.5 mM (red trace) and naphthalene diimide in DMF at 0.5 mM (blue trace). Blue dashed lines denote acceptor absorbance features and red lines donor absorbance features. Path length = 2 mm for all samples.

2.3 UV-vis absorption spectra of DA-PA assemblies

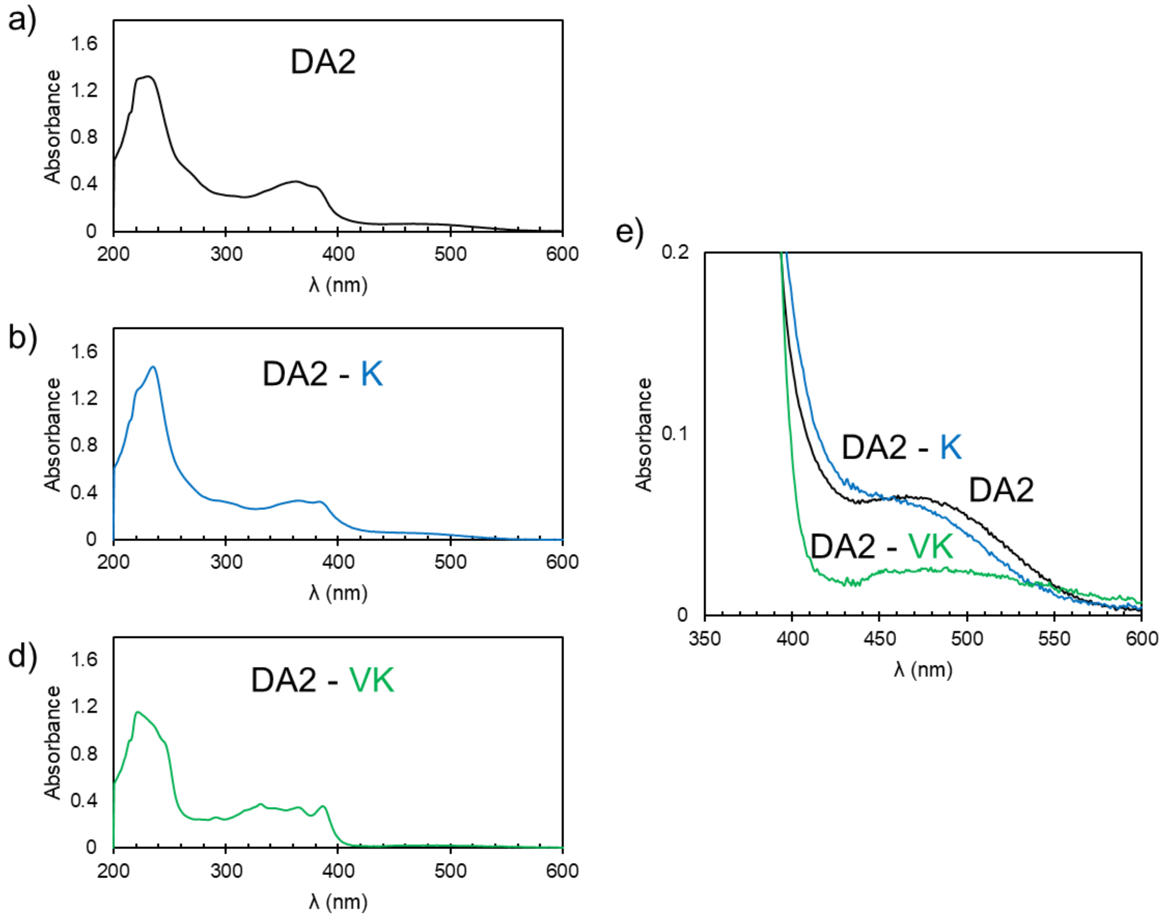

Supplementary Figure 7. UV-Vis absorbance of DA2-PAs in water and DMF. (a) **DA2** dissolved in water with 1 equiv. sodium hydroxide-grey trace. (b) **DA2-K**. (d) **DA2-VK**. (e) Overlay of CT band region. All samples were annealed at 1 mM and diluted to 200 mM before measurement.

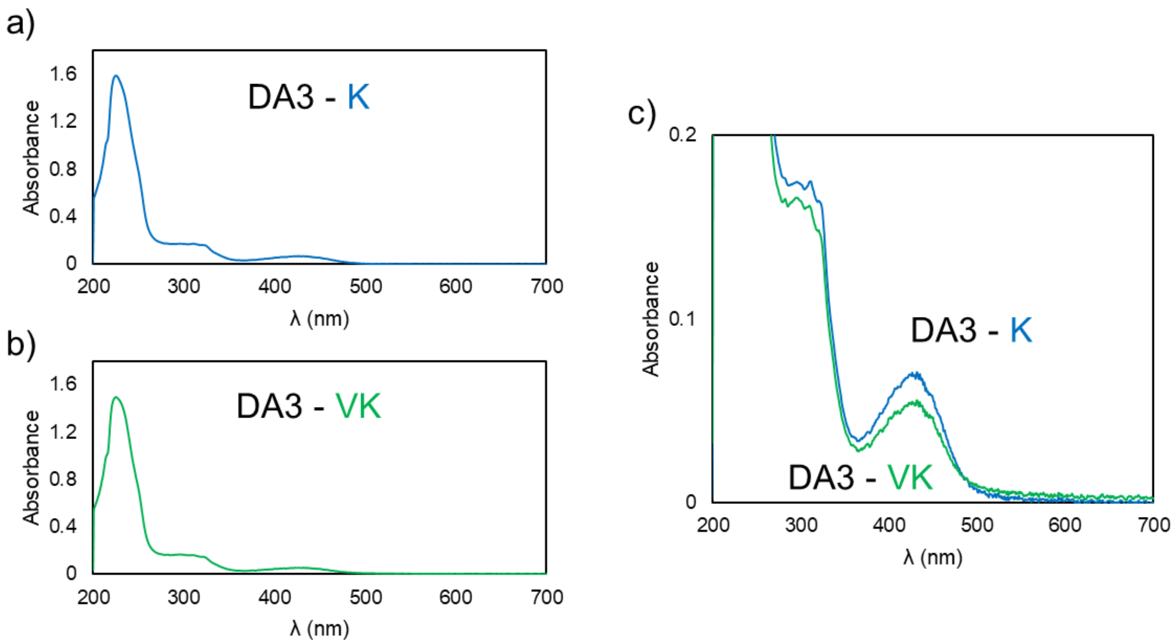

Supplementary Figure 8. UV-Vis absorbance of DA3-PAs in water and DMF. (a) **DA3-K**. (c) **DA3-VK**. (c) Overlay of CT band region. All samples were annealed at 1 mM and diluted to 200 mM before measurement.

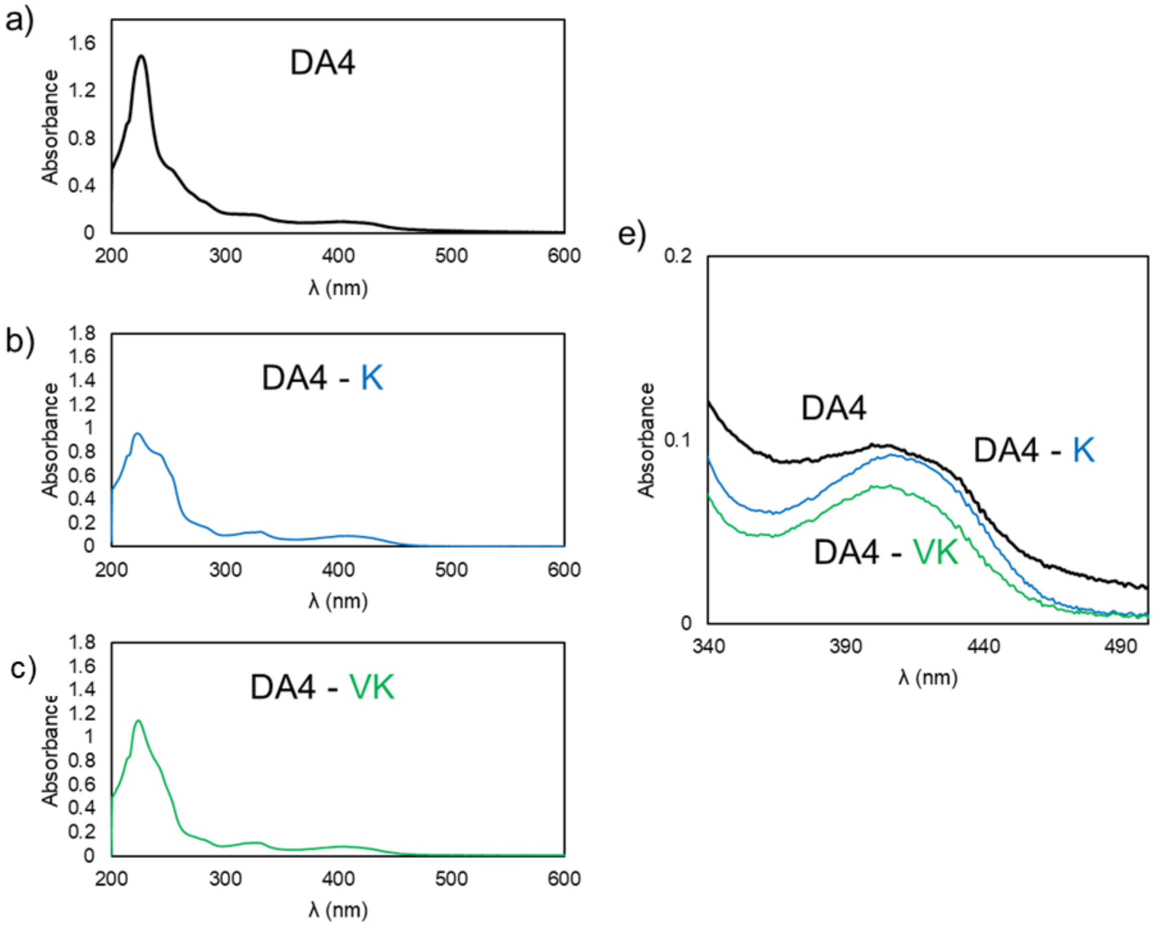

Supplementary Figure 9. UV-Vis absorbance of DA4-PAs in water and DMF. (a) **DA4** dissolved in water with 1 equiv. sodium hydroxide. Dissolved at 1 mM not annealed. (b) **DA4-K**. (c) **DA4-VK**. (e) Overlay of CT band region. **DA4-K** and **DA4-VK** were annealed at 1 mM and diluted to 200 mM before measurement.

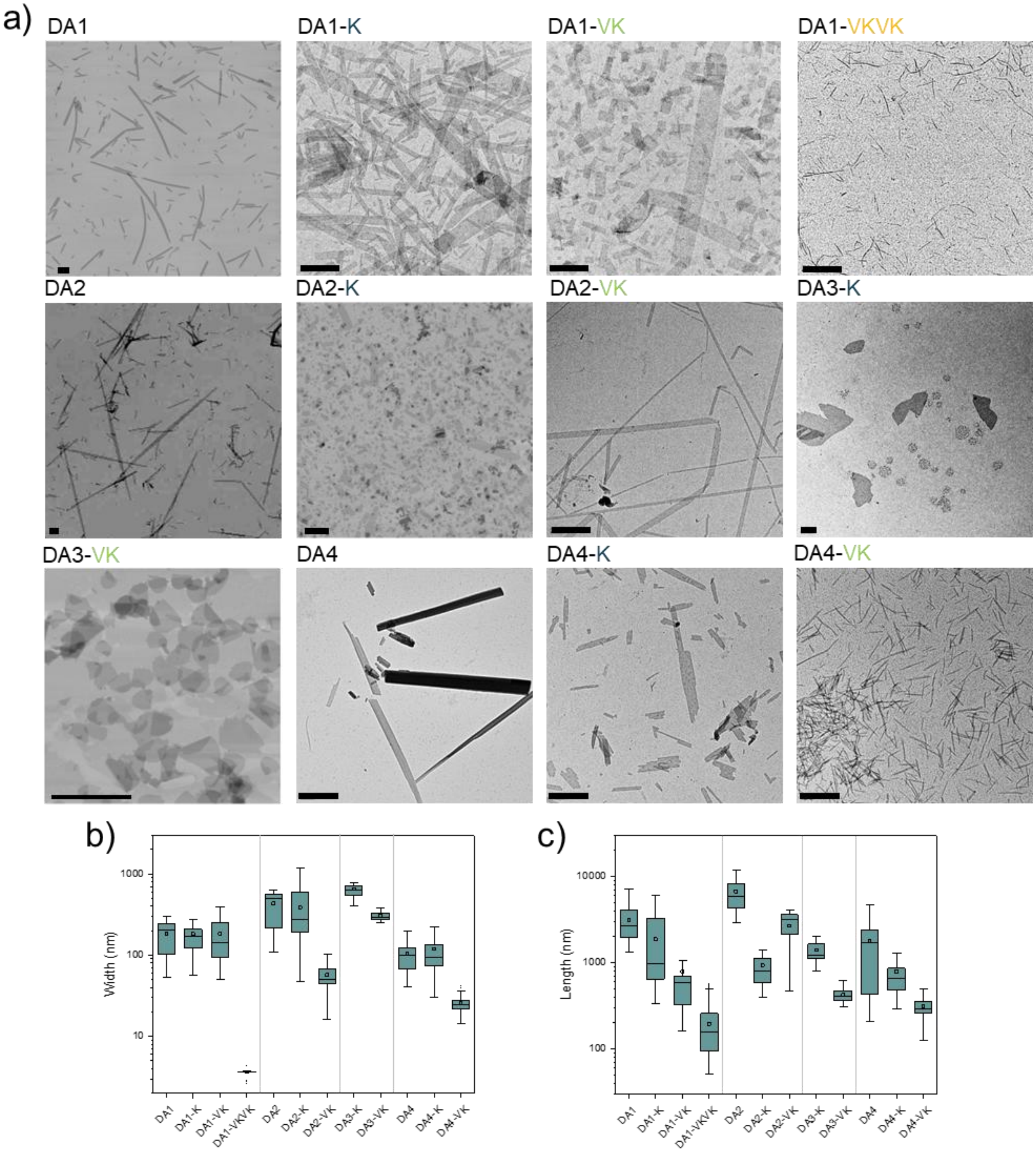

Supplementary Figure 10. Quantitative dimensions of DA-PA assemblies. (a) Representative images, (b) length and (c) width distributions of assemblies formed by DA-PAs. The scale bars in (a) represent 1 μm. The box plots in (b) and (c) show the first (25%) and third (75%) quartiles, median and mean values, and 1.5 interquartile range as the error bars obtained from more than 10 measurements.

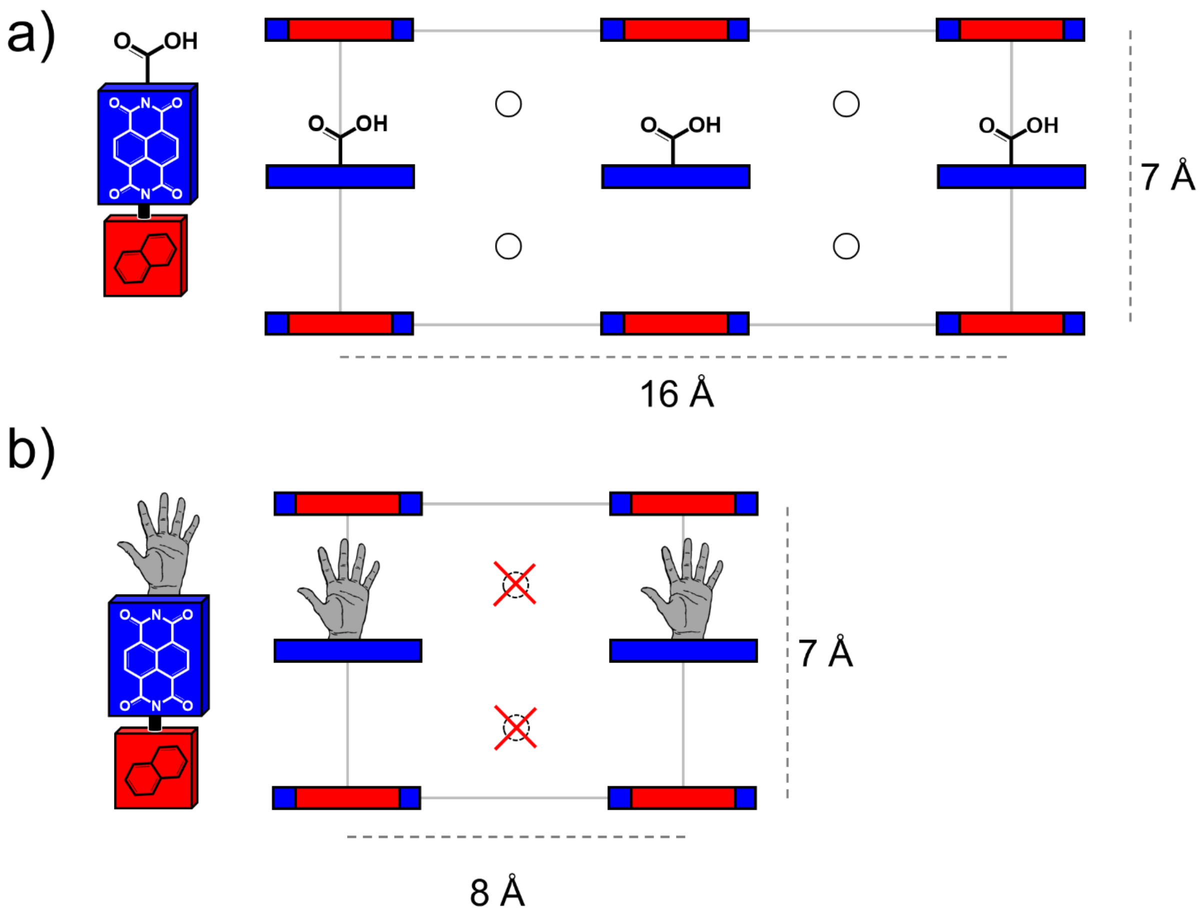

Supplementary Figure 11. Proposed molecular packing for DA1 and DA1-VK crystal lattices. (a) **DA1** centrosymmetric crystal structure with unit cell 7 Å × 16 Å. No chirality in molecular design and no observed SHG. (b) Proposed structure of **DA1-VK** with chiral dipeptide and SHG observed. Unit cell by diffraction is ½ the lateral dimension of **DA1**.

2.4 Selected area electron diffraction (SAED) and wide-angle X-ray scattering (WAXS) of DA and DA-PA assemblies

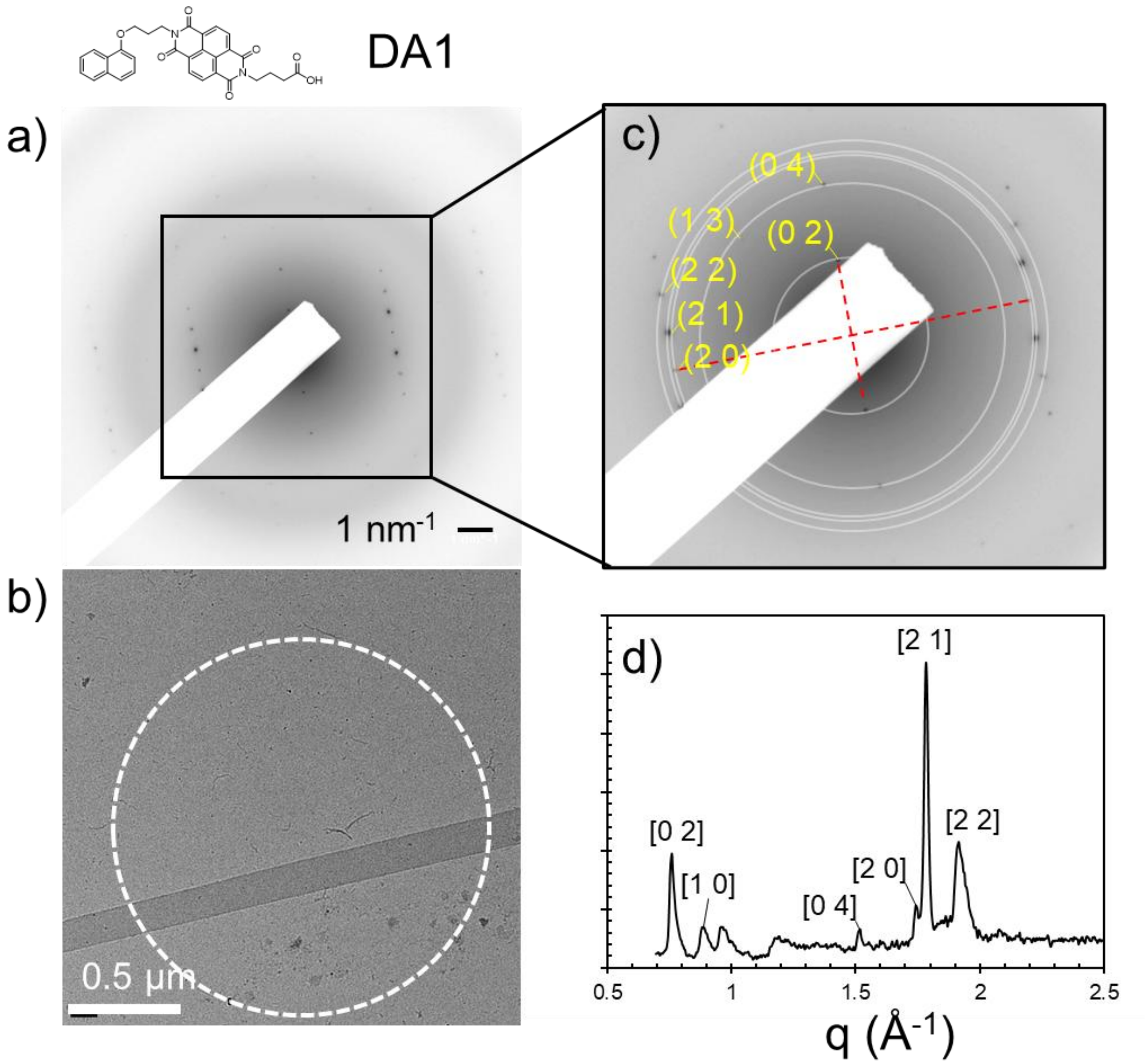

Supplementary Figure 12. SAED and WAXS patterns of DA1 assemblies. (a) **DA1** SAED and (c) indexing of diffraction pattern. (b) Corresponding micrograph with white circle indicating selected area. (d) Transmission WAXS with indexing of reflections.

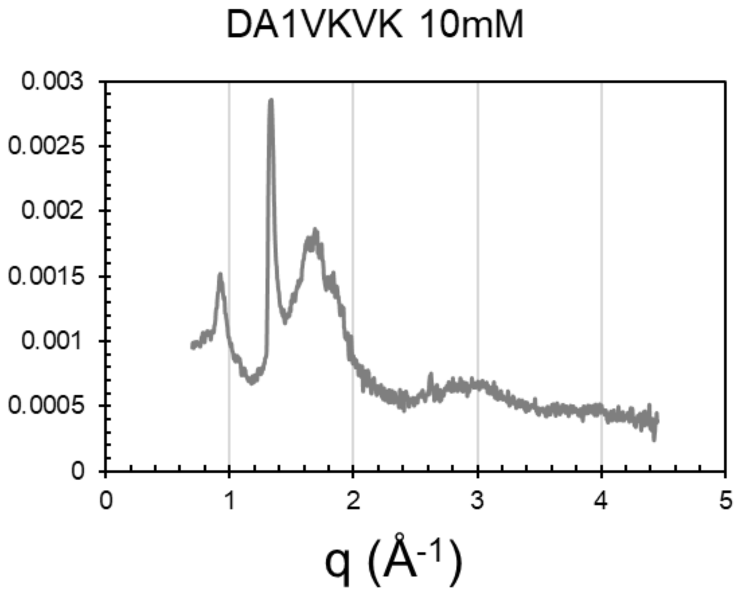

Supplementary Figure 13. WAXS pattern of DA1-VKVK assemblies. The WAXS pattern shows little relation to parent 2D lattices of **DA1** or **DA1-VK** but instead shows strong β-sheet signature.

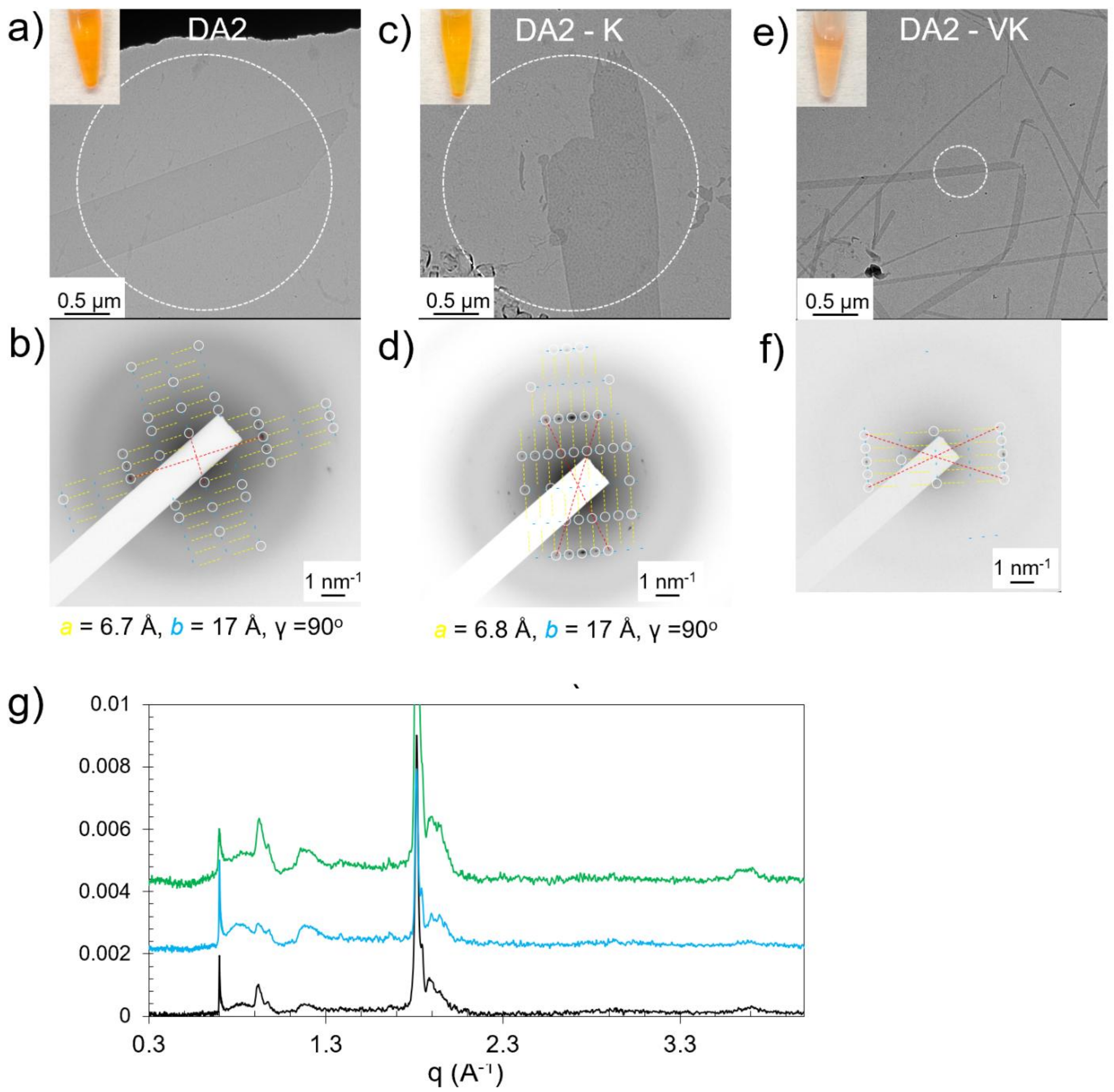

Supplementary Figure 14. SAED and WAXS patterns of DA2-PA assemblies. Transmission electron microscopy (TEM) of (a) **DA2**, (c) **DA2-K**, and (e) **DA2-VK**. SAED of (b) **DA2**, (d) **DA2-K**, (f) **DA2-VK**. (g) Transmission WAXS of **DA2** (black trace), **DA2-K** (blue trace), and **DA2-VK** (green trace).

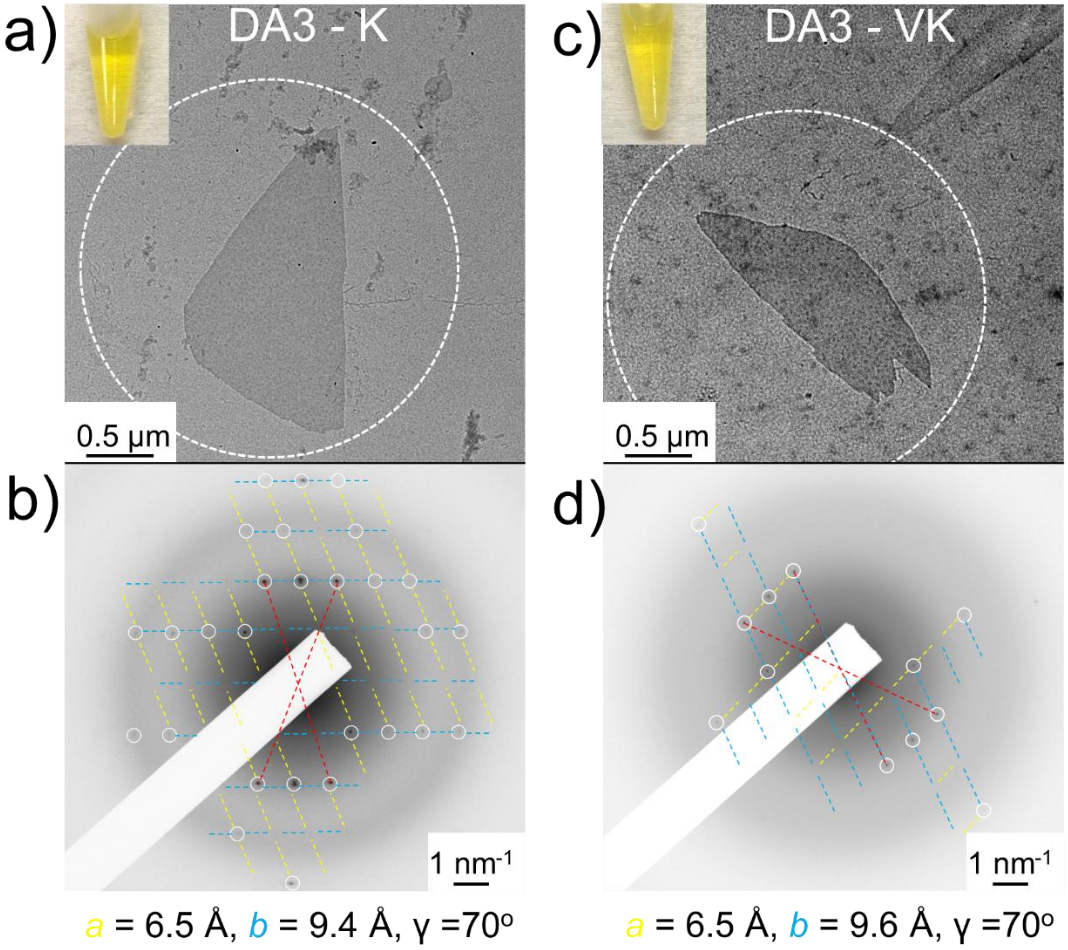

Supplementary Figure 15. SAED and WAXS patterns of DA3-PA assemblies. TEM of (a) **DA3-K** and (c) **DA3-VK**. SAED of (b) **DA3-K** and (d) **DA3-VK**.

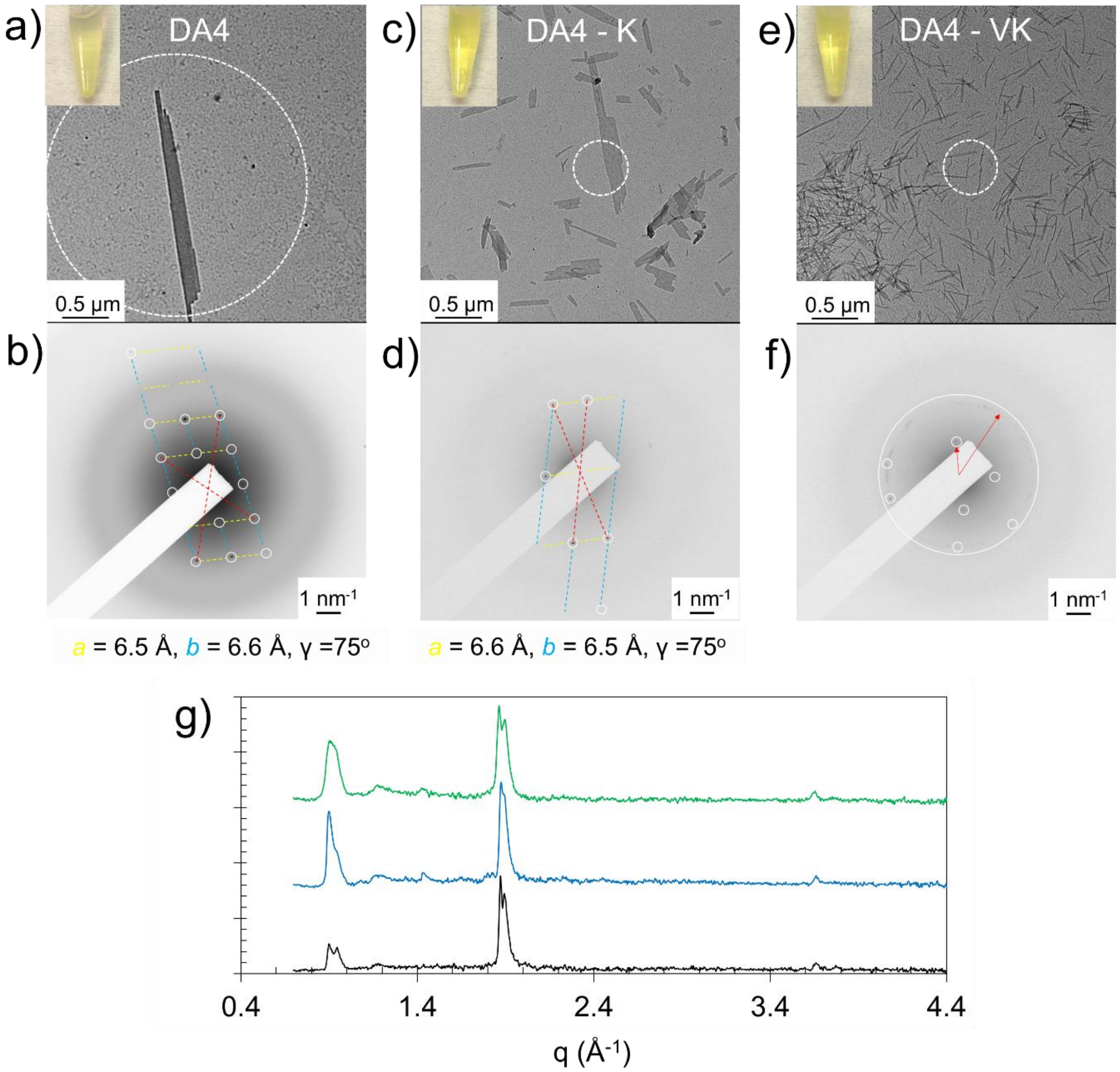

Supplementary Figure 16. SAED and WAXS patterns of DA4-PA assemblies. TEM of (a) **DA4**, (c) **DA4-K**, (e) **DA4-VK**. SAED of (b) **DA4**, (d) **DA4-K**, and (f) **DA4-VK**. Transmission WAXS of **DA4** (black trace), **DA4-K** (blue trace), and **DA4-VK** (green trace).

3 Chiral peptide induced symmetry breaking in DA-PA crystals

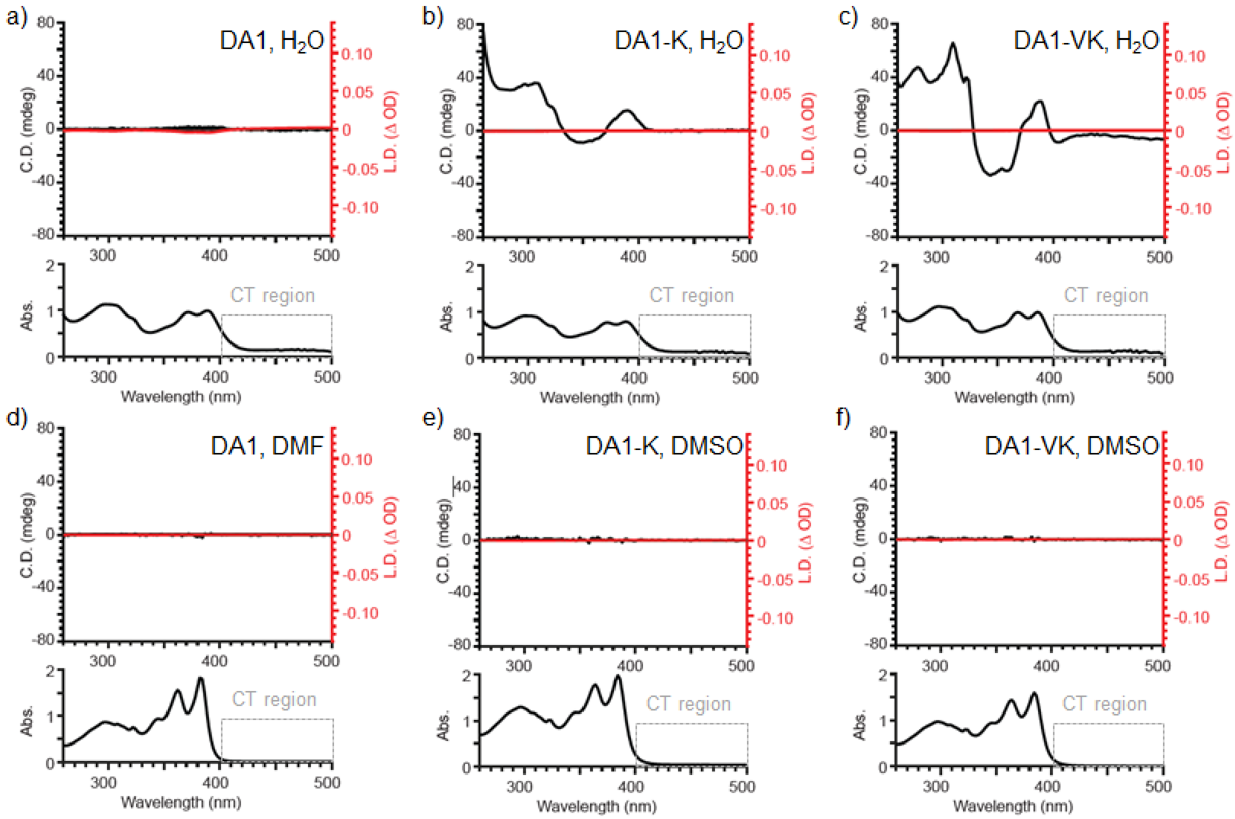

Supplementary Figure 17. Circular dichroism (CD) and linear dichroism (LD) spectroscopy of DA1-PAs in aqueous and organic media at the aromatic region. (a-c) CD and LD spectra of (a) **DA1**, (b) **DA1-K** and (c) **DA1-VK** in aqueous solutions. (d-f) CD and LD spectra of (a) **DA1** in DMF, (b) **DA1-K** in DMSO, and (c) **DA1-VK** in DMSO. **DA1** is dissolved with the addition of 1 equiv. NaOH in aqueous solutions. UV-Vis spectra of the samples are added below for comparison with the CT interaction region highlighted in dashed squares.

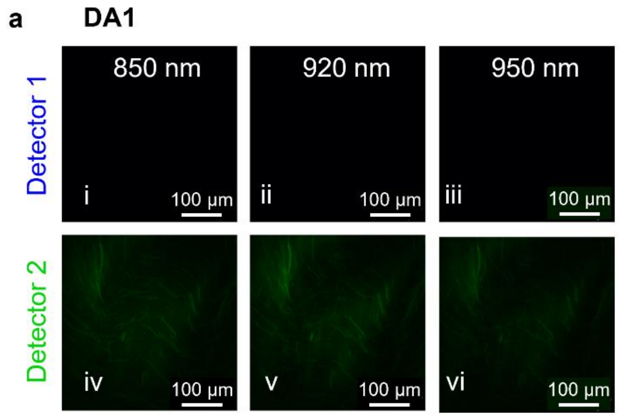

Supplementary Figure 18. Two-photon confocal microscopy of dried samples of DA1 and DA2 assemblies. (a) **DA1** and (b) **DA2** do not reveal clear SHG behavior. Detector 1 has a 380-450 nm detection window, and detector 2 has a 470-550 nm detection window.

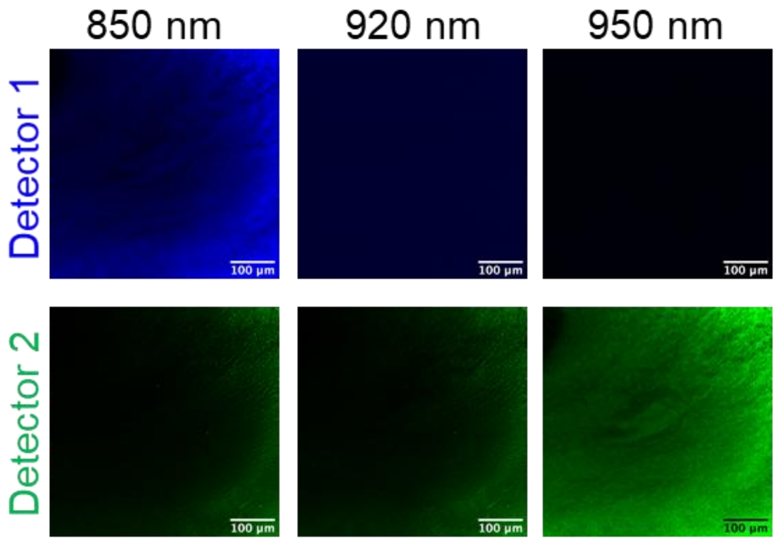

Supplementary Figure 19. DA1-K two-photon confocal microscopy reveals SHG activity. Detector 1 has a 380-450 nm detection window and detector 2 has a 470-550 nm detection window.

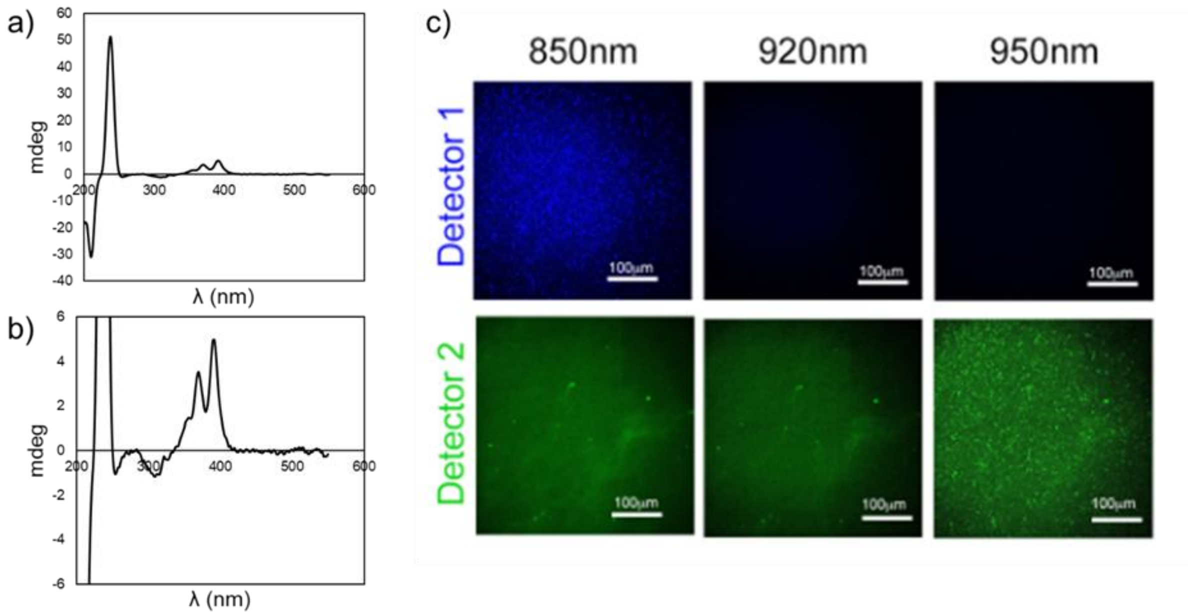

Supplementary Figure 20. CD spectroscopy and two-photon confocal microscopy of DA1-VKVK assemblies. (a) CD of **DA1-VKVK** and (b) y-scaled view. (c) **DA1-VKVK** two-photon confocal microscopy shows SHG. Detector 1 has a 380-450 nm detection window and detector 2 has a 470-550 nm detection window.

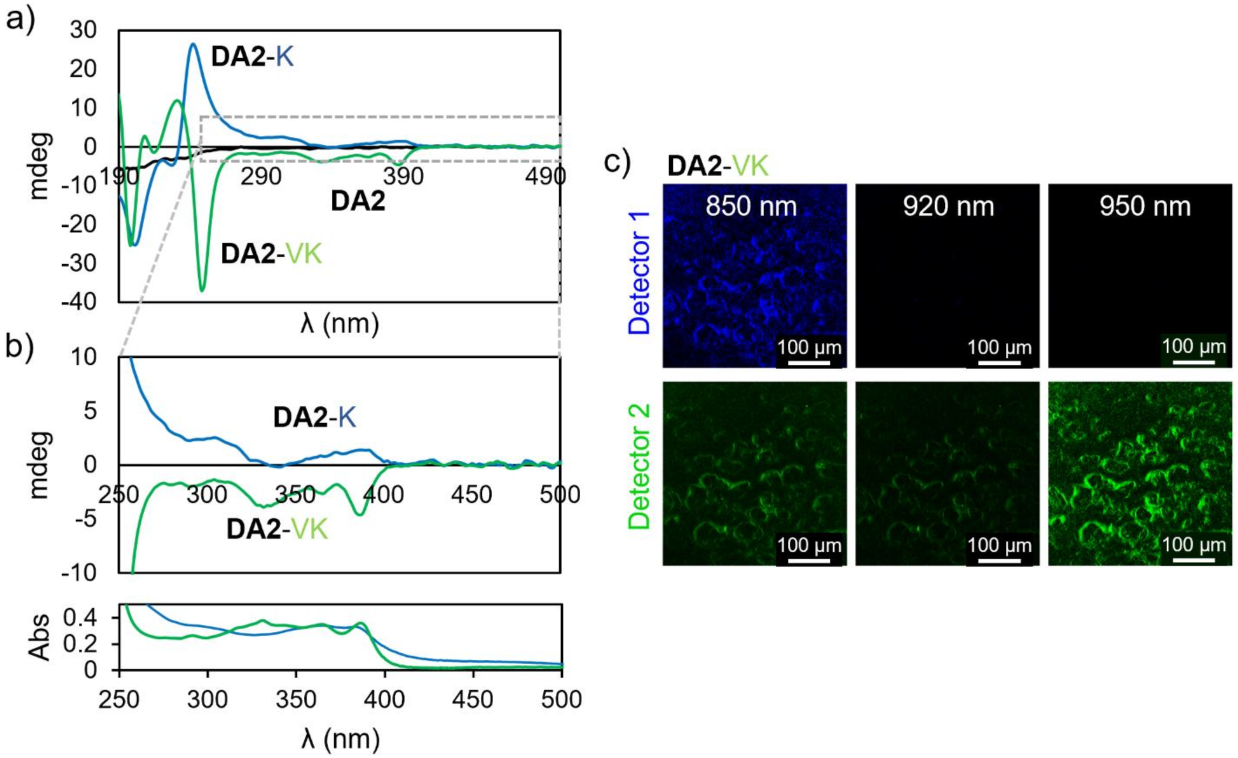

Supplementary Figure 21. CD spectroscopy and two-photon confocal microscopy of DA2-PA assemblies. (a) Circular dichroism absorption spectroscopy of aqueous solutions of **DA2** with 1 equiv. sodium hydroxide (black trace), **DA2-K** (blue trace), and **DA2-VK** (green trace). (b) Enlargement of aromatic region with UV-Vis absorption data for comparison (bottom). (c) **DA2-VK** two-photon confocal microscopy reveals SHG activity. Detector 1 has a 380-450 nm detection window, detector 2 has a 470-550 nm detection window.

Comparing the optical absorption spectra, the CT absorbance of **DA2-VK** is lower than that of **DA2-K** and the UV absorption bands are sharper than those of **DA2-K** (Supplementary Figure 21). These results support a distinct influence of the dipeptide on the structure of the DA chromophores despite insignificant changes to the observed electron or X-ray diffraction. Interestingly, **DA2-VK** has substantial pathway-dependent self-assembly behavior. For example, freshly dissolved solutions of **DA2-VK** produce one-dimensional fibrous structures that are not SHG active and show little crystalline ordering by transmission X-ray WAXS (Supplementary Figure 22). However, upon annealing, there is a change to the nanostructure morphology towards ribbons with widths greater than 50 nm and the appearance of crystalline ordering (Supplementary Figure 14).

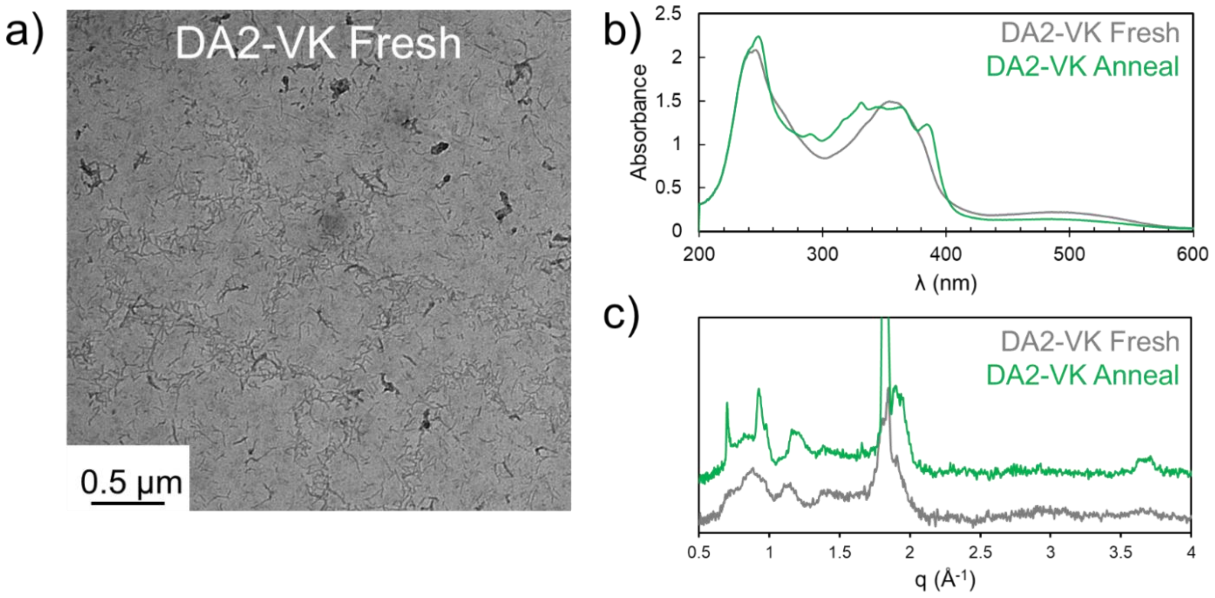

Supplementary Figure 22. TEM, UV-Vis and WAXS of freshly dissolved DA2-VK samples. (a) TEM micrograph showing small nanostructures. (b) Absorption spectroscopy showing CT absorption with broad UV absorption features (grey). (c) Transmission WAXS showing less ordering in the freshly dissolved sample (grey) as compared to annealed sample (green).

4 DFT geometry optimization and calculations

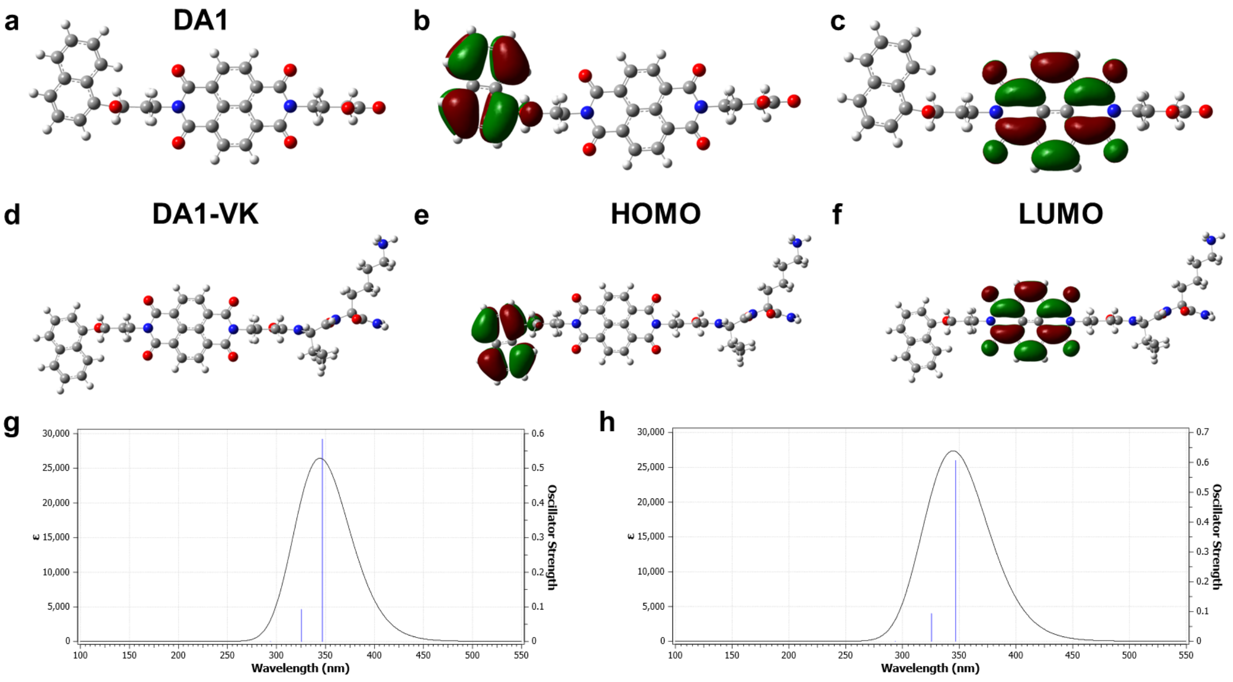

Supplementary Figure 23. DFT optimized geometry and TD-DFT calculations of DA1 and DA1-VK monomers. (a) **DA1** and (d) **DA1-VK** with the HOMO (b, e) localized on the naphthalene unit and the LUMO (c, f) on the NDI unit. (g) TD-DFT computed UV-Vis spectrum for a monomer of **DA1**. (h) TD-DFT computed UV-Vis spectrum for a monomer of **DA1-VK**.

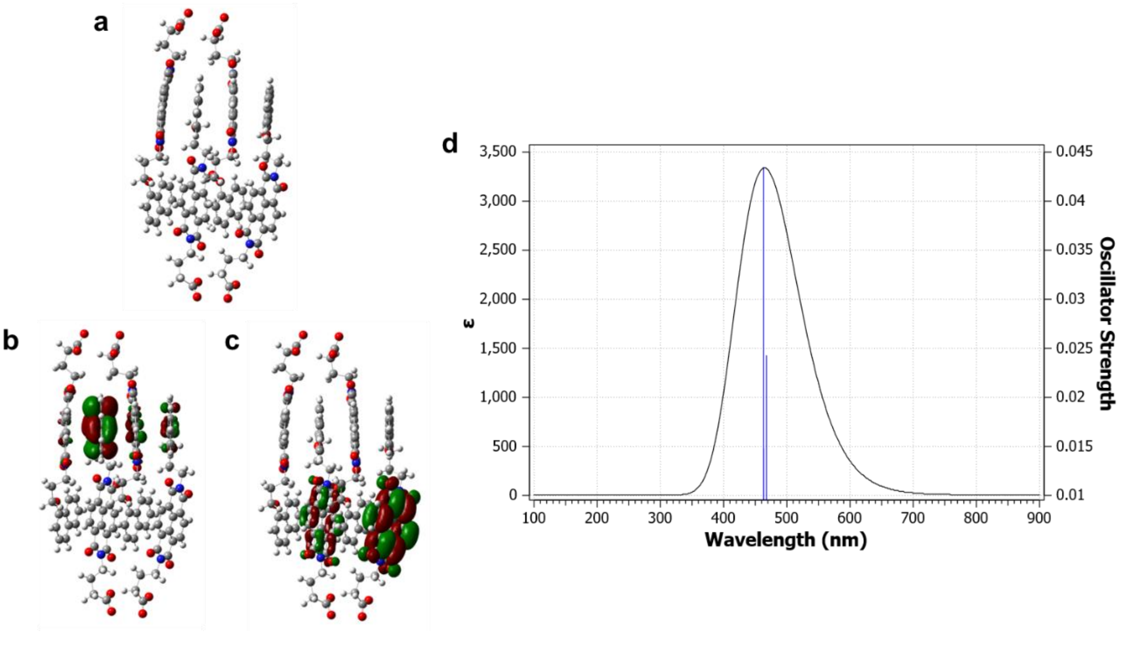

Supplementary Figure 24. DFT optimized geometry and TD-DFT calculations for a cluster of four DA1 molecules. (a) Optimized geometry for four **DA1** molecules showing a delocalized

HOMO (b) and LUMO (c) primarily centered on the naphthalene and NDI moieties, respectively. (d) TD-DFT computed UV-Vis spectrum for a cluster of four **DA1** molecules.

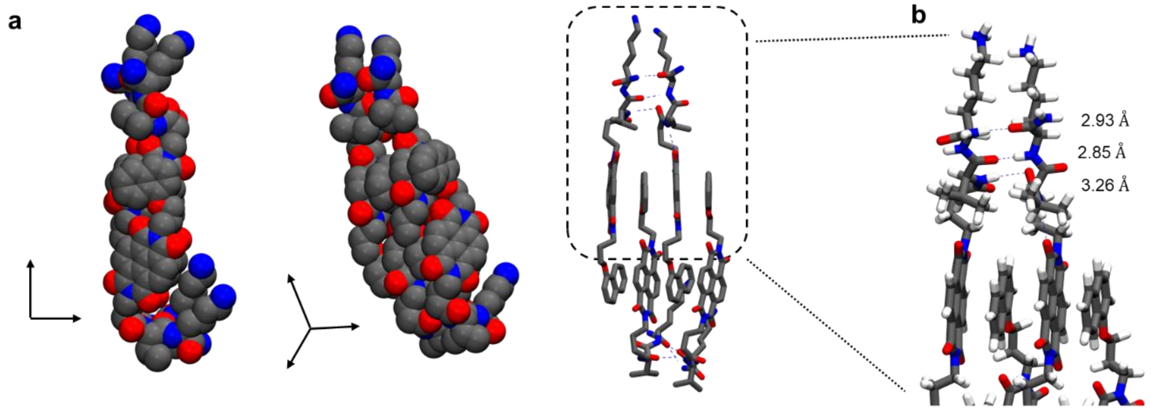

Supplementary Figure 25. DFT calculations for a cluster of four DA1-VK molecules. (a) Geometry optimized structure for the cluster of **DA1-VK** depicted without hydrogens and as vdW representation showing VK sidechain conformational flexibility. (b) Hydrogen bonds between the VK-NH_2 peptide backbones shown as dashed blue lines with distances between hydrogen bond donor-acceptor atoms listed.

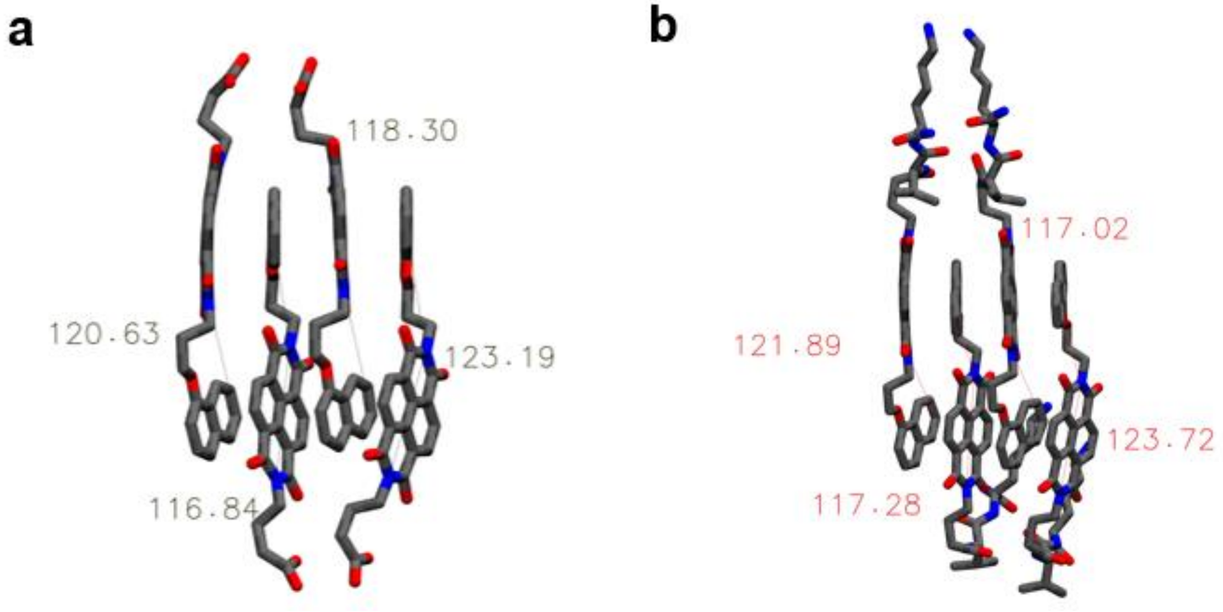

Supplementary Figure 26. DFT optimized structures for clusters of DA1 and DA1-VK. D-A tilt angle (in ˚) comparisons between (a) a cluster of four **DA1** molecules and (b) a cluster of four **DA1-VK** molecules.

5 Racemized DA-PA assemblies

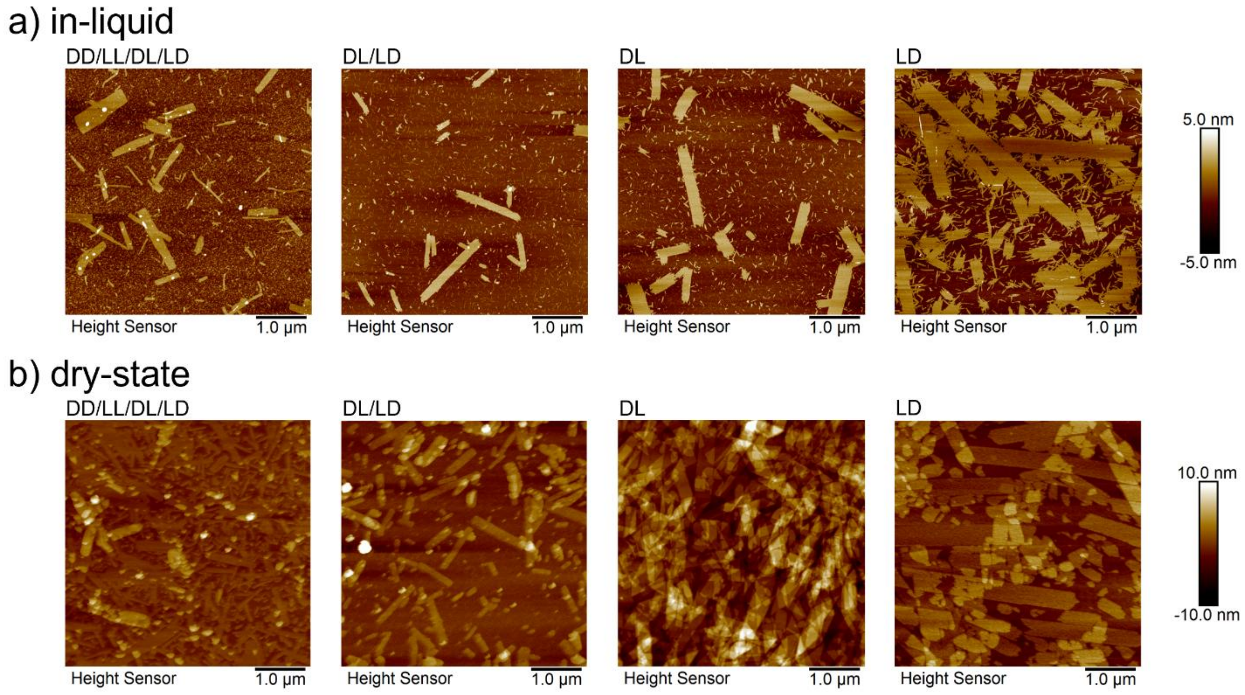

Supplementary Figure 27. AFM images of the racemized DA2-PA nanostructures. Co-assembly of **DA2-(D)V-(D)K/DA2-VK/DA2-(D)V-K/DA2-V-(D)K** in the ratio of 1/1/1/1 (**DD/LL/DL/LD**), co-assembly of **DA2-(D)V-K/DA2-V-(D)K** in the ratio of 1/1 (**DL/LD**), **DA2-(D)V-K** (**DL**), and **DA2-V-(D)K** (**LD**) were measured on (a) in-liquid and (b) dry-state samples after annealing with the total concentration of peptide amphiphile at 5 mM.

The co-assembled systems show smaller ribbon sizes compared to **DA2-VK** which is attributed to the different *β*-sheet structures formed by racemic peptide mixtures[2]. CD measurements of **DA2-V-(D)K** show predominantly the same positive ellipticity as **DA2-VK** in the chromophore absorption range while the **DA2-(D)V-K** and **DA2-(D)V-(D)K** show the expected negative ellipticity (Supplementary Figure 28). This is because valine has a high propensity to form the chiral *β*-sheet secondary structure and the charged lysine does not contribute to the *β*-sheet formation in solution.

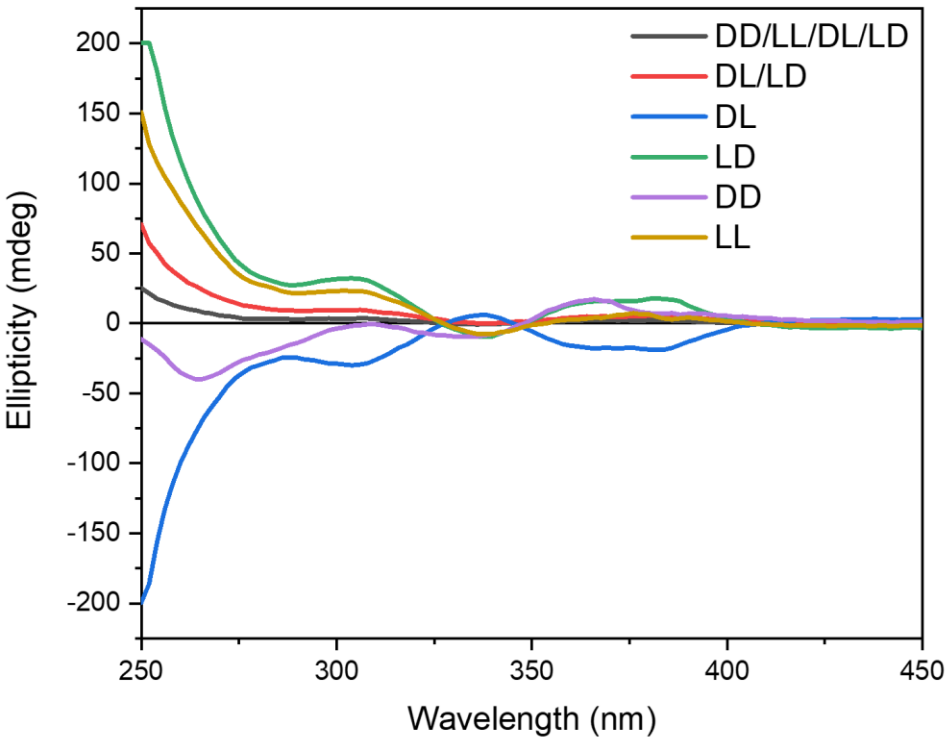

Supplementary Figure 28. CD absorption spectroscopy of the racemized DA2-PA nanostructures. DD/LL/DL/LD, **DL/LD**, **DL**, **LD**, **DD**, and **LL** were measured in the chromophore absorption range. All samples were annealed with the total concentration of peptide amphiphile at 5 mM.

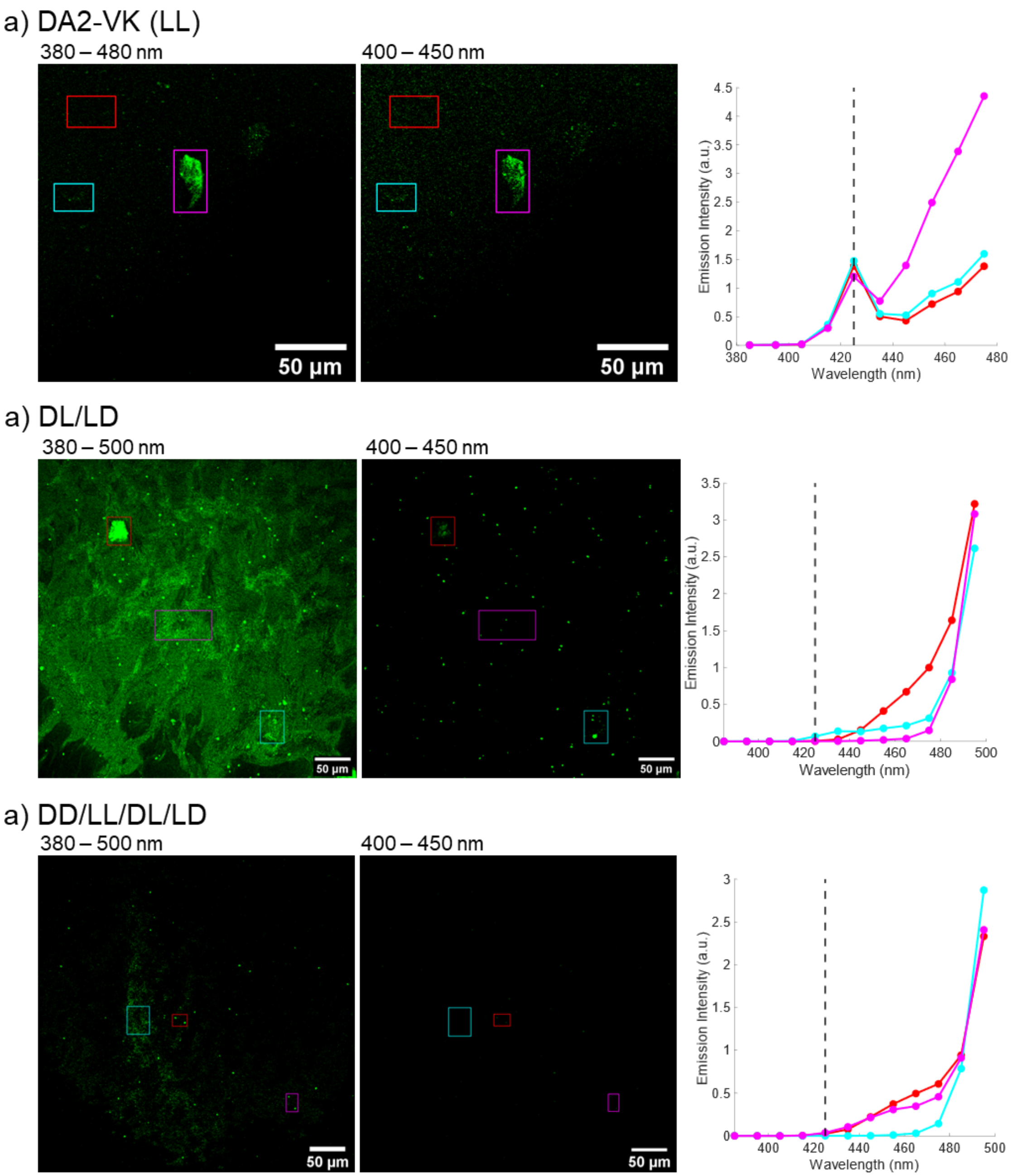

Supplementary Figure 29. Two-photon confocal microscopy of the racemized DA2-PA nanostructures. (a) **LL**, (b) **DL/LD**, and (c) **DD/LL/DL/LD**. All samples were dried from annealed solution with the total concentration of peptide amphiphile at 5 mM. The detection windows of 380-500 nm and 400-450 nm correspond to the full range and SHG active range of the emission spectrum at the excitation wavelength of 850 nm. Emission intensity data were acquired every 10 nm with the data points present the median wavelength. The vertical dashed lines indicate the wavelength where SHG occurs.

6 Electrical properties of DA-PA crystals

6.1 Linear capacitance and resistance values of the DA-PA samples

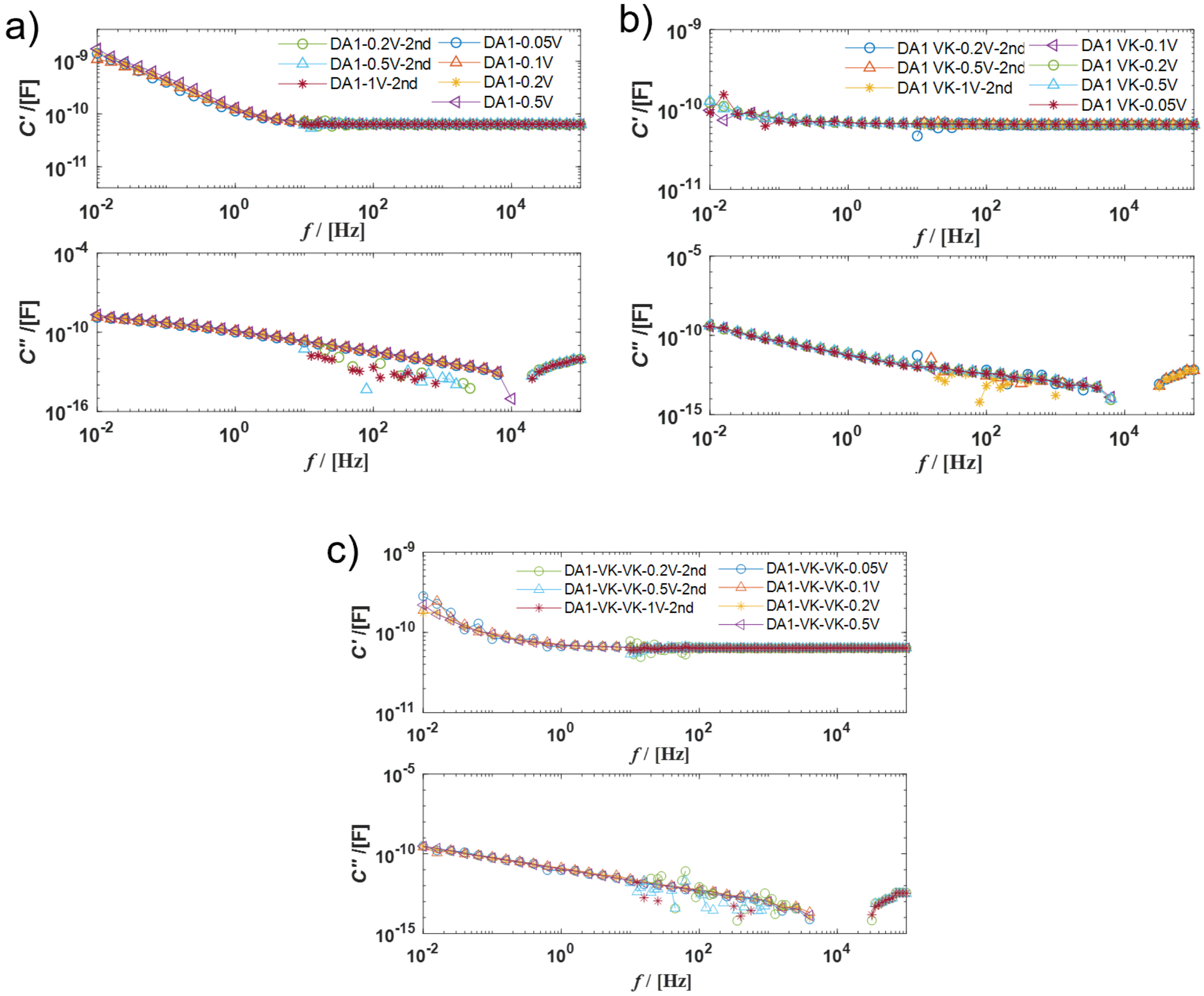

Supplementary Figure 30. Complex capacitance of DA1-PA assemblies. (a) **DA1**, (b) **DA1-VK** and (c) **DA1-VKVK** were measured under different voltages using Metrohm AutoLab PGSTAT-128N (0.05 to 0.5 V) and Solartron 1260 (0.2 to 1.0 V, distinguished by suffix '-2nd') indicating the linearity of measured capacitance. The real capacitance *C*' near the base frequency of the triangular waveform applied in P-E loop measurement (20 Hz) is used as the linear capacitance of the sample.

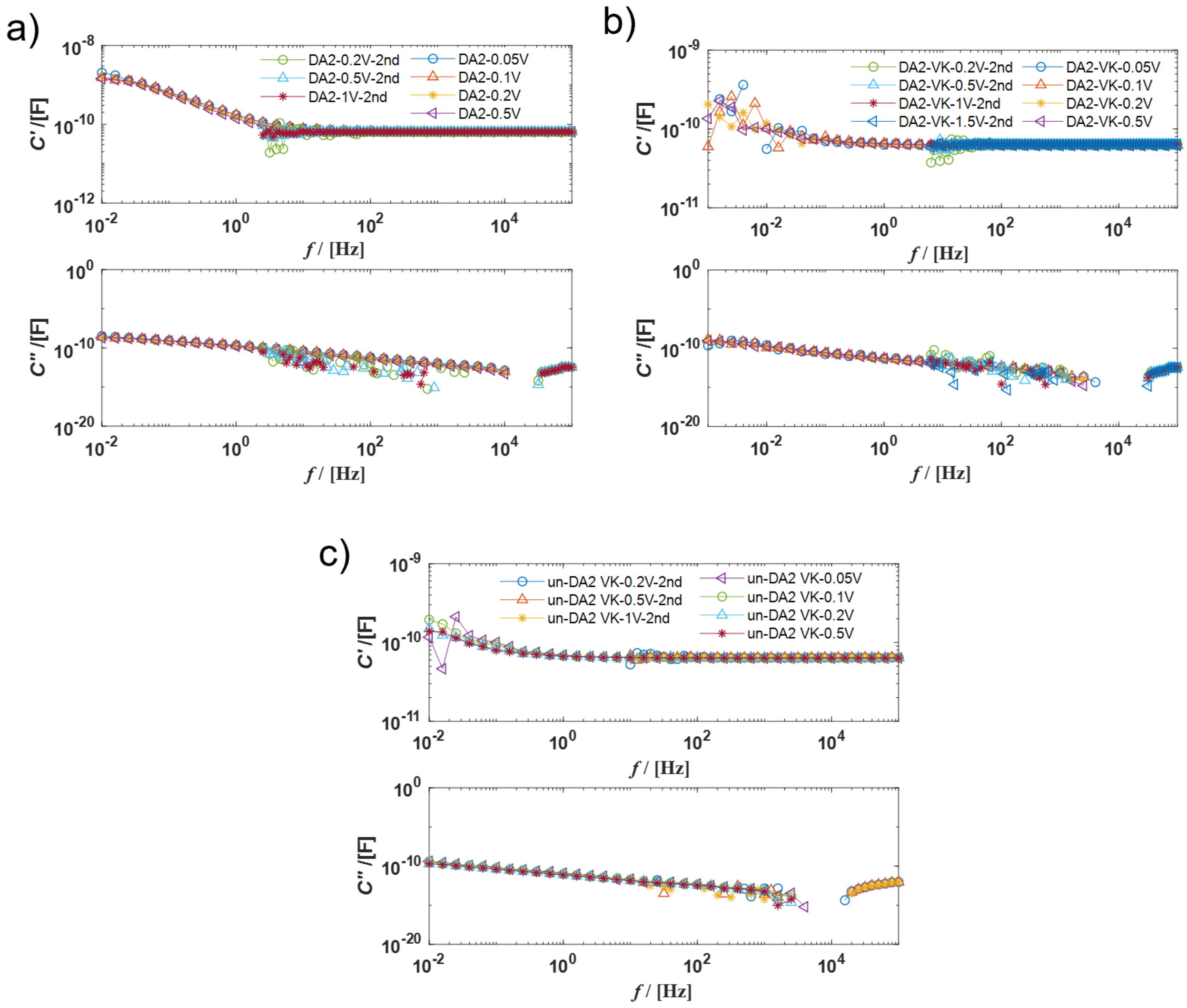

Supplementary Figure 31. Complex capacitance of DA2-PA assemblies. (a) **DA2**, (b) **DA2-VK** and (c) unannealed **DA2-VK** were measured under different voltages using Metrohm AutoLab PGSTAT-128N (0.05 to 0.5 V) and Solartron 1260 (0.2 to 1.0 V, distinguished by suffix '-2nd') indicating the linearity of measured capacitance. The real capacitance *C*' near the base frequency of the triangular waveform applied in P-E loop measurement (20 Hz) is used as the linear capacitance of the sample.

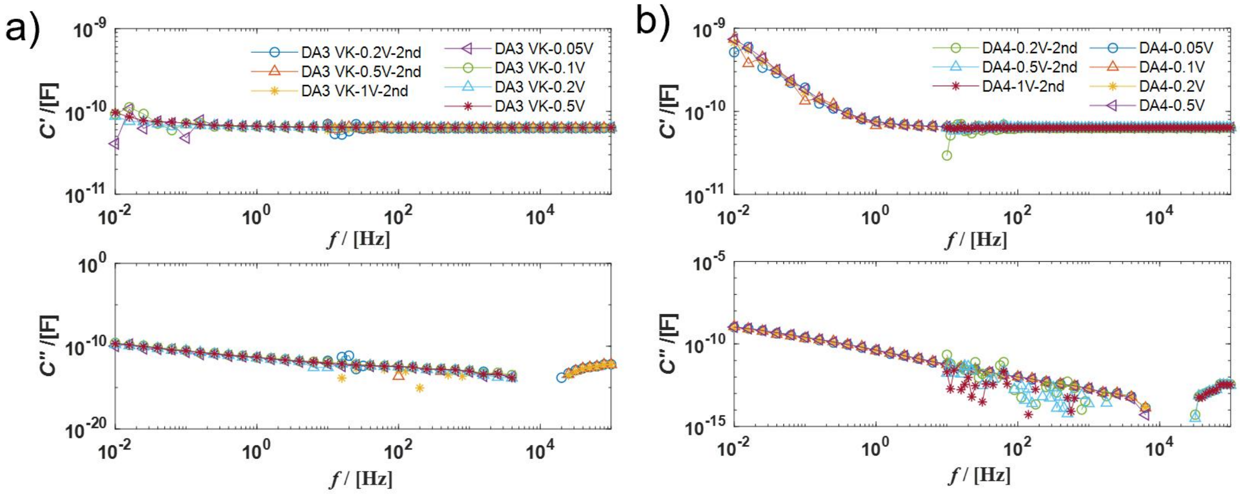

Supplementary Figure 32. Complex capacitance of DA3-VK and DA4. (a) **DA3-VK** and (b) **DA4** were measured under different voltages using Metrohm AutoLab PGSTAT-128N (0.05 to 0.5 V) and Solartron 1260 (0.2 to 1.0 V, distinguished by suffix '-2nd') indicating the linearity of measured capacitance. The real capacitance *C*' near the base frequency of the triangular waveform applied in P-E loop measurement (20 Hz) is used as the linear capacitance of the sample.

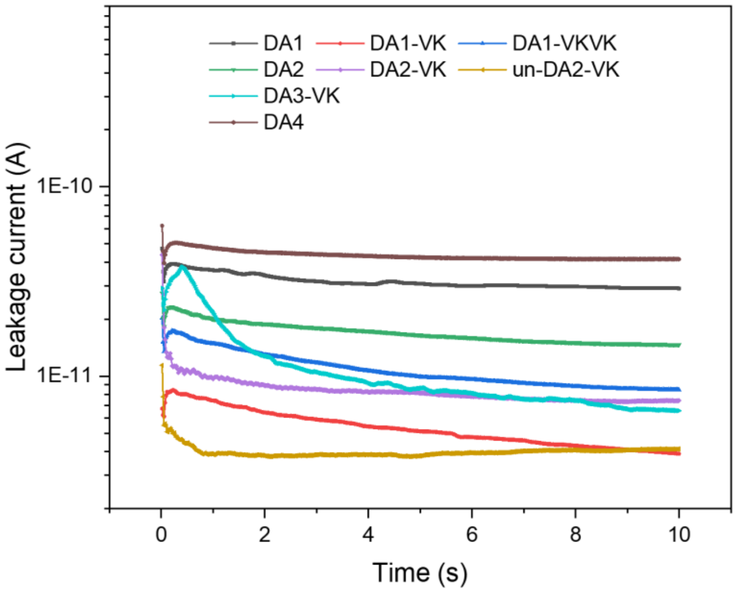

Supplementary Figure 33. Leakage current of the samples under a DC voltage of 1.0 V.

Supplementary Table 1. The C_1 and R_1 values of the samples calculated from the frequency-domain impedance and leakage current measurements.

	DA1	DA1-VK	DA1-VKVK	DA2	DA2-VK	un-DA2-VK	DA3-VK	DA4
C_1 ($\times 10^{-11}$ F)[a)]	6.563	6.586	6.589	6.674	6.333	6.556	6.429	6.588
R_1 ($\times 10^{9}$ Ω)	34.3	255	117	67.9	134	242	151	24.0

[a)] The average values of the real parts of the complex capacitance obtained from 15~25 Hz data points from Supplementary Figs. 30-32, around the 20 Hz base frequency of the triangular waveform applied in P-E loop measurement.

6.2 Data processing of P-E loops

The linear capacitor C_1 corresponds to the linear polarization mechanisms including electrons/ions elastic displacement, dipole reorientation, etc., and the resistor R_1 is related to the conduction loss of the sample[3]. The linear capacitance C_1 of the samples was determined by frequency-domain impedance measurements (Supplementary Figure 30 to 32) and the resistance R_1 of the samples was obtained from the leakage current measurements (Supplementary Figure 33). The introduction of the peptide head group improves the resistance of DA-PA compared to the pure donor-acceptor samples (Supplementary Table 1), which is favorable for potential ferroelectric functionalities. The contribution of C_1 to polarization, P_{C1}, is

$$P_{C1} = C_1 Ed / S \tag{1}$$

where E is the applied electric field, d is the channel width, S is the cross-sectional area of the electrode. According to the Sawyer–Tower circuit of the ferroelectric tester, the contribution of R_1 to polarization under sine waveform, P_{R1}, can be derived from the following equation:

$$\left(SR_1\omega/U_0\right)^2 P_{R1}^2 + \left(d / U_0\right)^2 E^2 = 1 \tag{2}$$

where ω is the angular frequency of the applied voltage, and U_0 is the voltage amplitude. For the applied triangle waveform, the base frequency component of the waveform is used to calculate P_{R1}. As an example, the P-E loops of non-ferroelectric **DA1** and ferroelectric **DA2-VK** before and after subtracting the contribution of C_1 and R_1 are shown in Supplementary Figure 34.

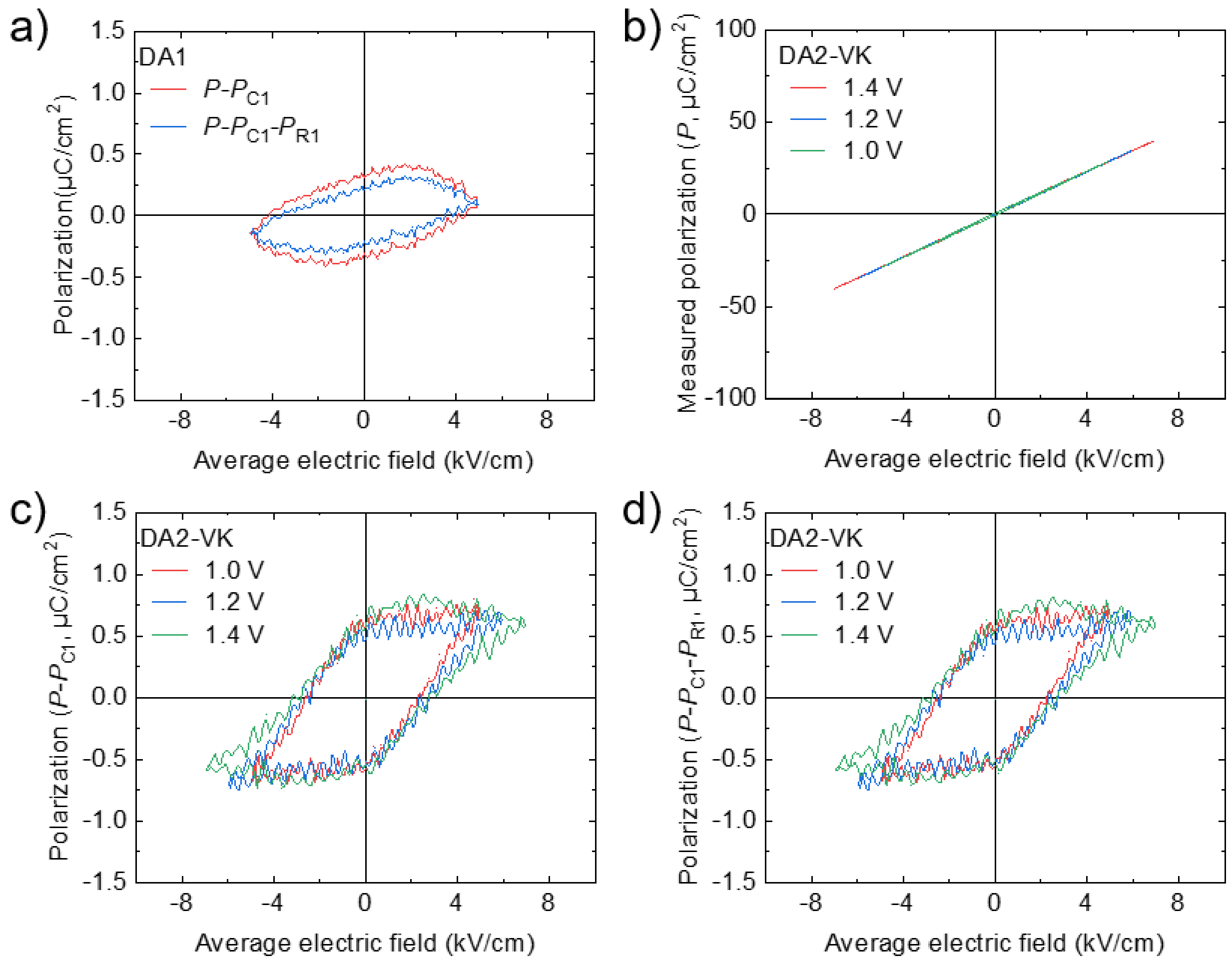

Supplementary Figure 34. Data processing of P-E loops measured on DA2-VK assemblies. P-E loops of non-ferroelectric **DA1** (a) and ferroelectric **DA2-VK** (b-d) before and after subtracting the contribution of C_1 and R_1. Measured polarization P (b), P-E loops after subtracting the contribution of linear capacitance C_1, P-P_{C1} (c), and P-E loops after subtracting the contribution of linear resistance R_1, P-P_{C1}-P_{R1} (d) of **DA2-VK**.

6.3 Morphologies and electrical properties of DA-PA samples

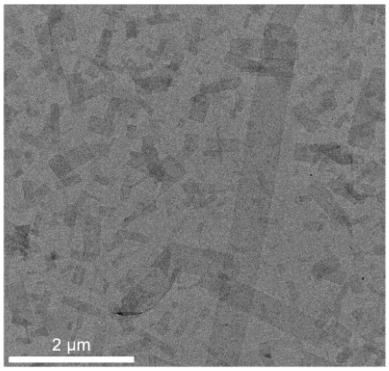

Supplementary Figure 35. TEM micrograph of DA1-VK assemblies. Polydisperse ribbon sizes are observed with many small aggregates much smaller than 2 µm width of test device.

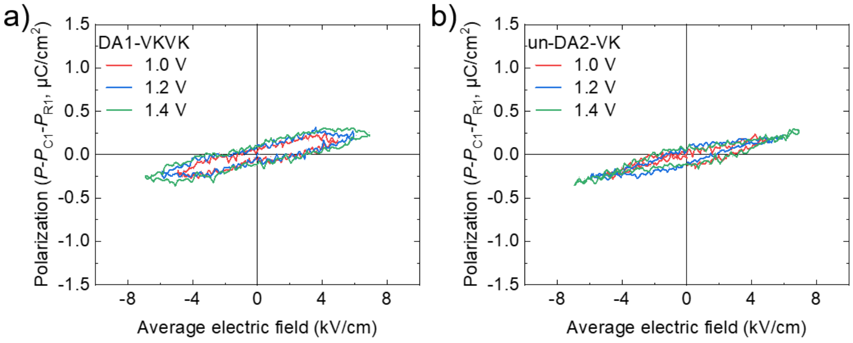

Supplementary Figure 36. P-E loops of DA1-VKVK assemblies and DA2-VK prepared from unannealed solution. Both samples show lossy dielectric behavior.

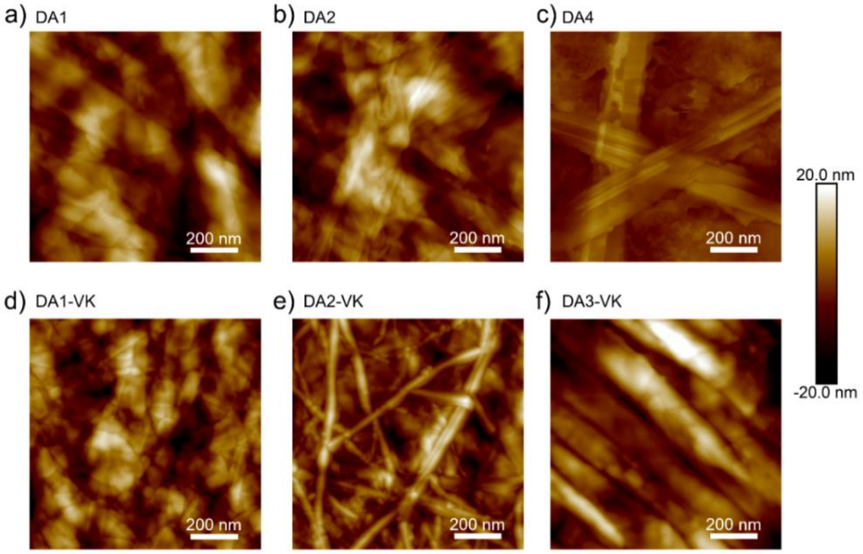

Supplementary Figure 37. AFM morphology of the dry-state DA and DA-PA nanostructures on the testing substrate.

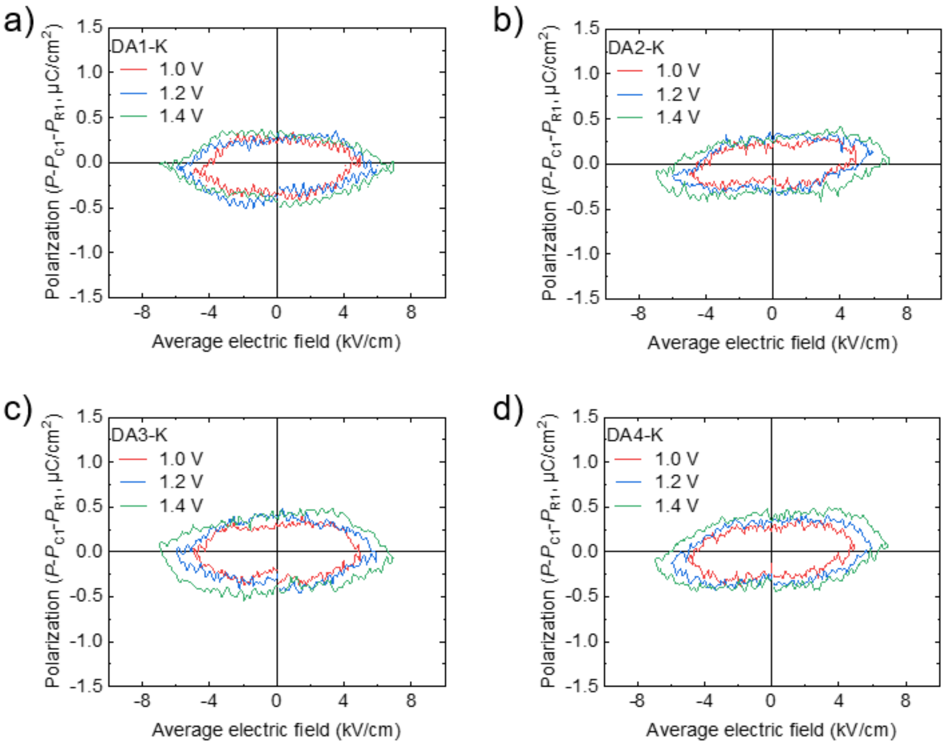

Supplementary Figure 38. The P-E loops of (a) DA1-K, (b) DA2-K, (c) DA3-K and (d) DA4-K. Measurements of linear capacitance C_1 and resistance R_1 are shown in Supplementary Figure 39.

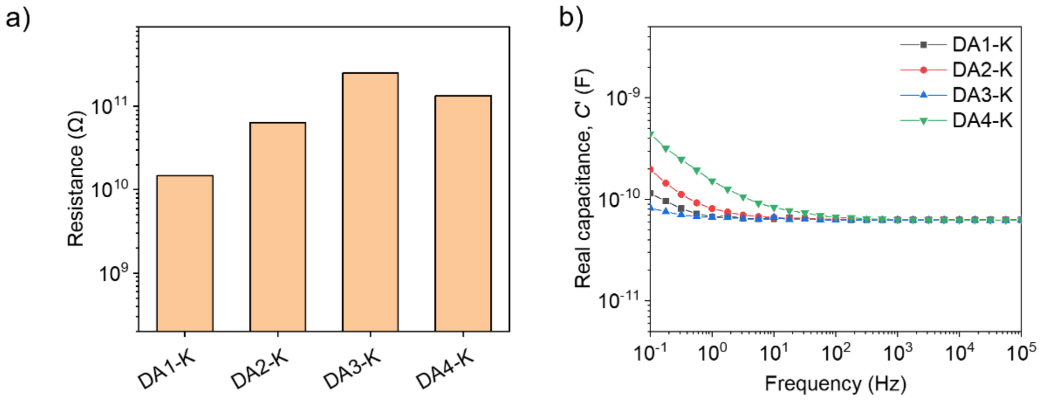

Supplementary Figure 39. Resistance and linear capacitance of DA-K samples. (a) Resistance of the **DA1-K**, **DA2-K**, **DA3-K** and **DA4-K** samples obtained from the leakage current measurement under 1.0 V DC voltage for 10 s. (b) Real capacitance C' of the **DA1-K**, **DA2-K**, **DA3-K** and **DA4-K** samples measured under 0.5 V voltage amplitude.

6.4 Electrical properties of racemized DA-PA samples

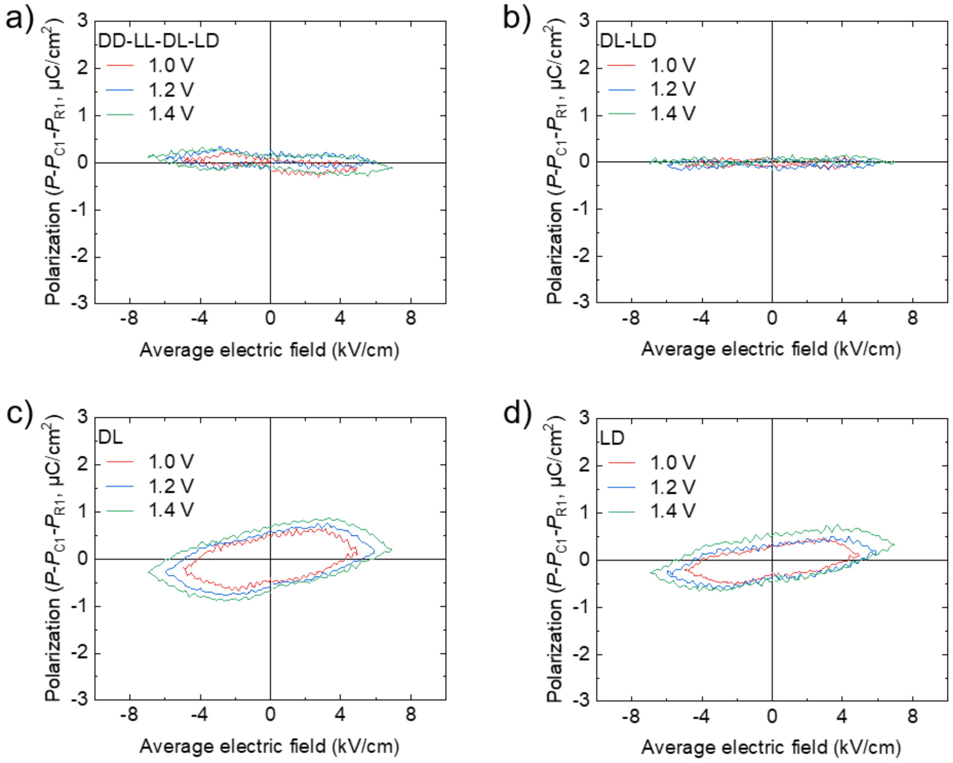

Supplementary Figure 40. The P-E loops of racemized DA-PA samples. (a) **DD/LL/DL/LD**, (b) **DL/LD**, (c) **DL**, and (d) **LD**. Measurements of linear capacitance C_1 and resistance R_1 are shown in Supplementary Figure 41.

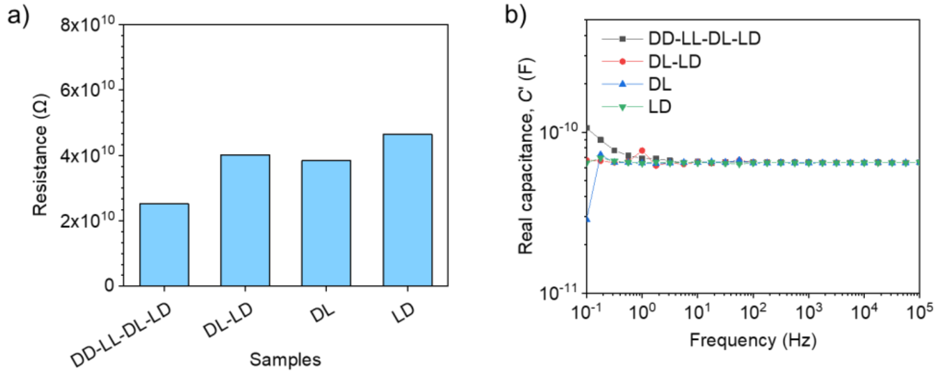

Supplementary Figure 41. Resistance and linear capacitance of racemized DA-PA samples. (a) Resistance of the **DD/LL/DL/LD**, **DL/LD**, **DL**, and **LD** samples obtained from the leakage current measurement under 1.0 V DC voltage for 10 s. (b) Real capacitance C' of the **DD/LL/DL/LD**, **DL/LD**, **DL**, and **LD** samples measured under 0.5 V voltage amplitude.

6.5 Low-field ferroelectric properties and piezoelectric responses of DA-PA samples

Supplementary Table 2. Comparison of P_r, E_c and P_r/E_c values among ferroelectric DA-PA assemblies and other metal-free room-temperature organic ferroelectrics.

Legends in Fig.		P_r (μC/cm^2)	E_c (kV/cm)	P_r/E_c (nF/m)	Year[ref.]
DA-PA assemblies	**DA1-VK**	0.355	5.40[b)]	6.60	**this work**
	**DA2-VK**	0.530	4.21[b)]	12.6	
	**DA3-VK**	0.195	3.15[b)]	6.20	
PVDF- and OVDF-based ferroelectrics	VDF_6-VEVE[a)]	0.650	3.49[b)]	18.6	2024[4]
	VDF_6-VEVE (aligned)	3.80	8.72[b)]	43.6	
	PBI-OVDF	0.550	230	0.239	2016[5]
	Pc-OVDF	3.70	205	1.81	
	P(VDF-TrFE)	10.0	490	2.04	2006[6]
CT complexes/co crystals	PMI-Py-1	1.50	8.50	17.6	2012[7]
	PMI-Np	2.00	14.0	14.3	2017[8]
	AN-F_4TCNQ	0.001	0.2	0.5	2018[9]
	NDI-Py	1.20	8.50	14.1	2022[10]
	P1-Py	0.250	25.0	1.00	2022[11]
	BTA-NDI	0.210	11.0	1.91	2023[12]
hydrogen-bonding crystals	$HOCH_2(CF_2)_3CH_2OH$	6.34	18.0	35.2	2024[13]
	(−)-camphanic acid	5.20	50.0	10.4	2022[14]

Abbreviations: AN=acenaphthene, F_4TCNQ=2,3,5,6-tetrafluoro-7,7,8,8-tetracyanoquinodimethane, Py=pyrene, NDI=naphthalene-diimide, P1=NDI-appended polyurethane, PBI=perylenes bisimide, Pc=phthalocyanines, BTA=benzene tricarboxamide.

a) Measured on the randomly cast sample under a voltage amplitude of 1.4 V.

b) Due to the nonuniform electric field generated by the in-plane electrodes, the E_c values are calibrated by multiplying the coefficient 1.53 according to our previous research using the same experimental setup[4].

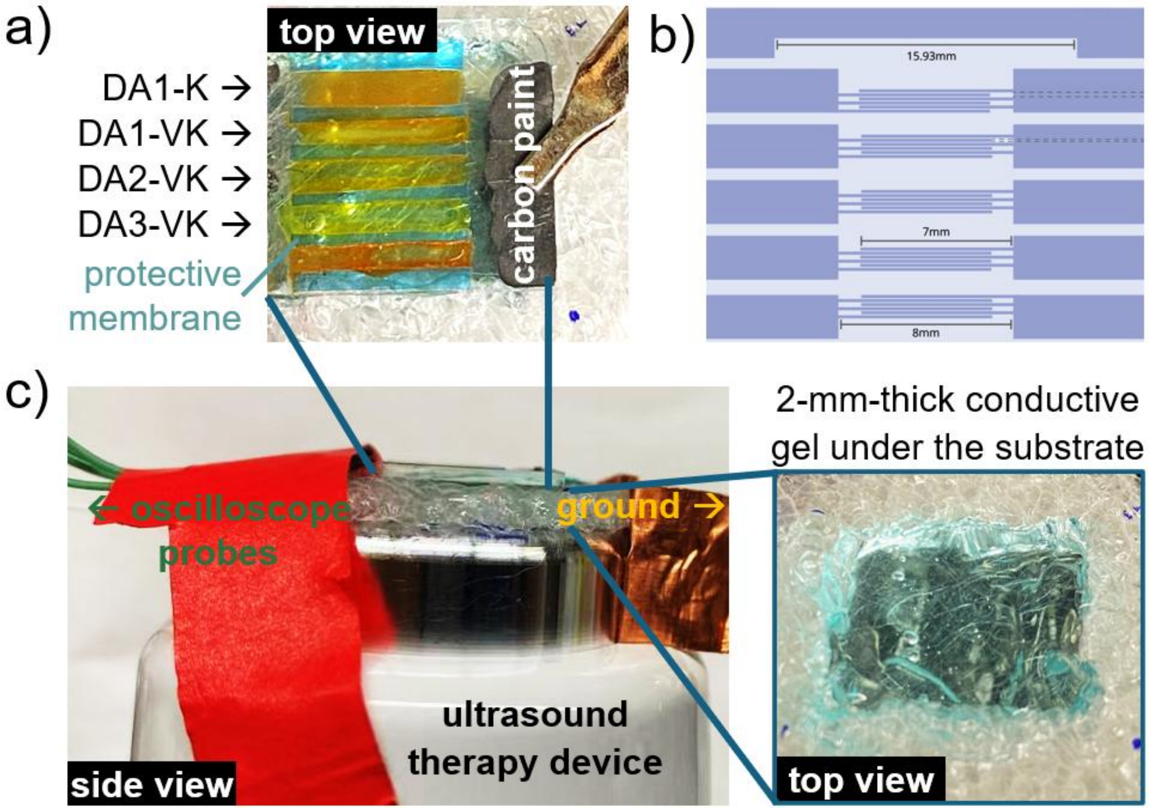

Supplementary Figure 42. The experimental setup for measuring the piezoelectric response of DA-PA samples. (a) A top-view photograph of the dry-state DA-PA samples coated on individual channels of interdigitated indium tin oxide (ITO) electrodes isolated by the plastic protective membrane. (b) A schematic of the ITO electrode pattern on the testing substrate. Each electrode channel features a constant gap size of 50 μm and a length of 30 (5×6) mm. (c) A side-view photograph of the sample substrate mounted on an ultrasound therapy device. The zoom-in top-view photograph shows the 2-mm-thick conductive gel applied for transmitting ultrasonic stimulation.

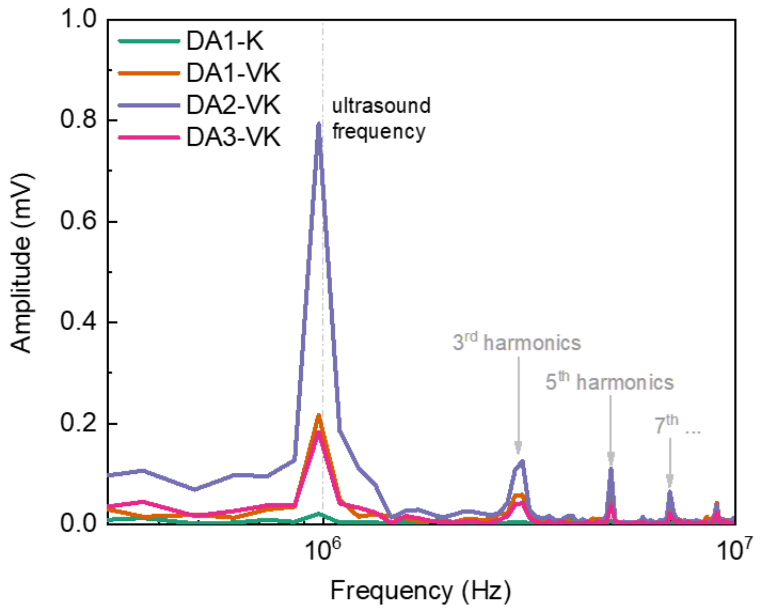

Supplementary Figure 43. Fast Fourier transform (FFT) analysis of the piezoelectric voltage waveforms in Figure 4k.

The longitudinal open-circuit piezoelectric voltage can be derived as

$$U = gYst \tag{1}$$

where g is the piezoelectric voltage coefficient, Y is Young's modulus, s is strain, and t is the sample thickness (electrode gap size). Considering the electrostriction approximation[15]

$$g \approx 2QP_r \tag{2}$$

where Q is the electrostrictive coefficient and P_r is the remnant polarization, the relationship between the piezoelectric voltage and remnant polarization can be derived as

$$U \approx 2QP_rYst \tag{3}$$

Considering their similar chemical compositions, we assume that the samples of DA-VK assemblies share similar values of Q and Y. This explains the observed correlation between the highest piezoelectric voltage and remnant polarization observed on **DA2-VK** among the ferroelectric DA-VK samples. Due to the inherent inhomogeneity of the supramolecular nanomaterial coatings and the in-plane configuration of the electrode setup, the measured piezoelectric voltages are intended solely for qualitative comparison among the DA-PA samples.

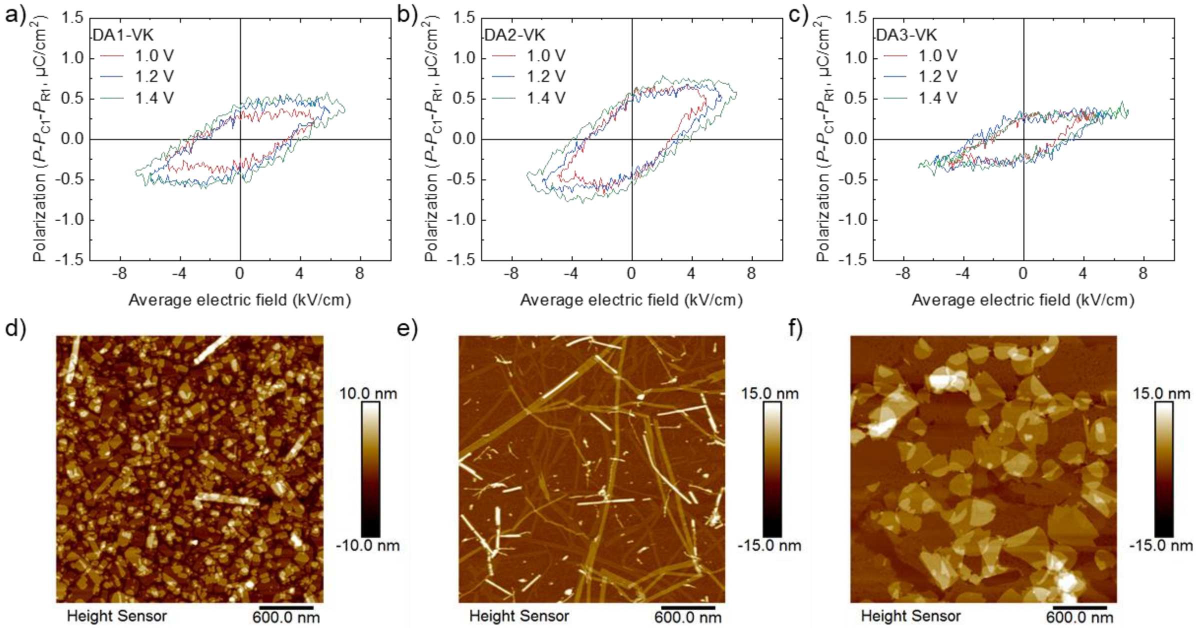

Supplementary Figure 44. P-E loops and morphologies of a different batch of ferroelectric DA-PA samples. (a-c) P-E loops of **DA1-VK** (a), **DA2-VK** (b), **DA3-VK** (c). (d-f) AFM morphologies of **DA1-VK** (d), **DA2-VK** (e), **DA3-VK** (f).

7 Neuron experiments

7.1 Neuronal culture on DA-PA coatings

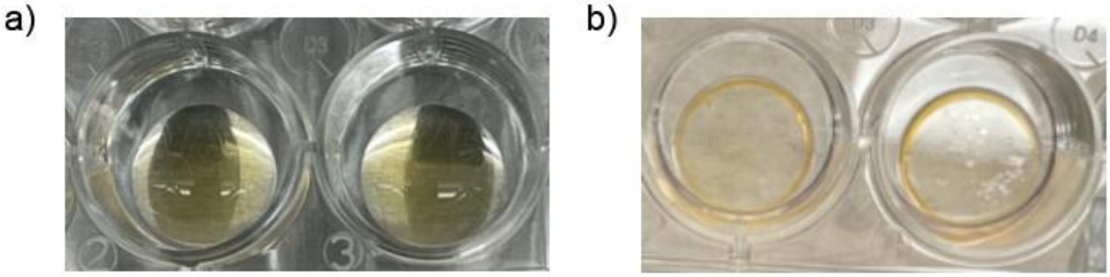

Supplementary Figure 45. Evaporation method for preparing DA-PA coated poly-D-lysine (PDL) glass coverslips. (a) Coverslip immediately following the application of 1 mM solution of DA-PA. (b) DA-PA coated coverslip following overnight evaporation.

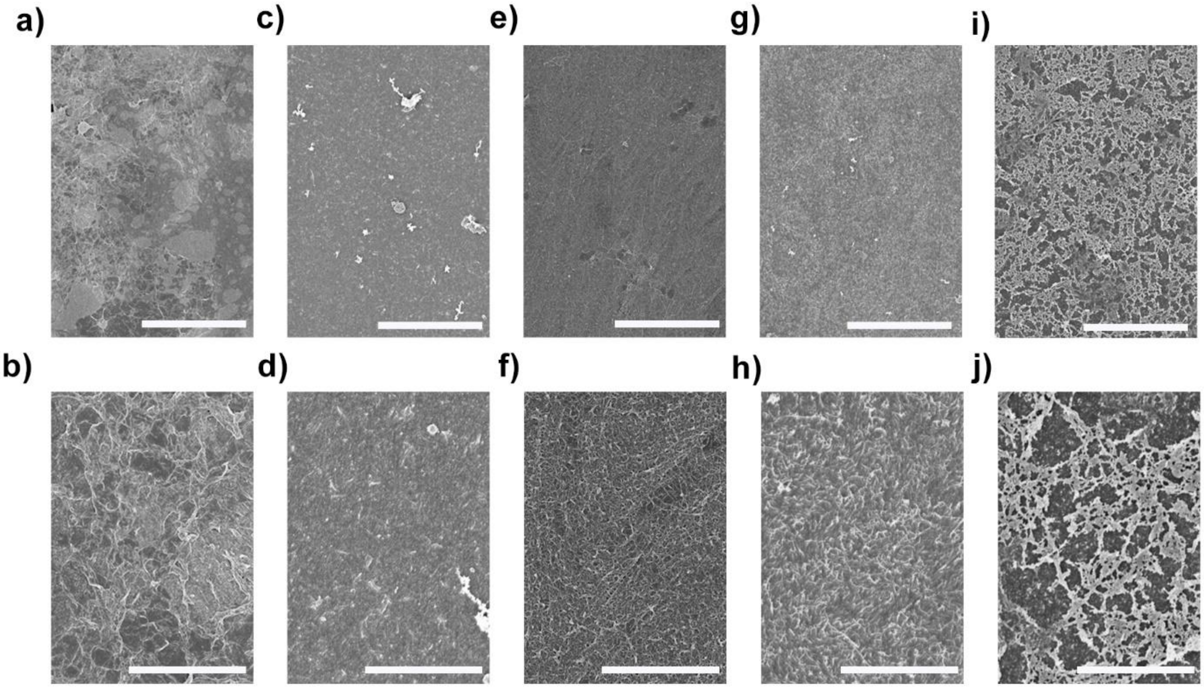

Supplementary Figure 46. Scanning electron microscopy (SEM) images of DA-PA coated PDL glass coverslips prepared via the evaporation method after 48 hours of culture. (a-b) **DA1-K**, (c-d) **DA1-VK**, (e-f) **DA1-VKVK**, (g-h) **DA2-VK**, and (i-j) **DA3-VK**. Scale bar a, c, e, g, i = 20 µm. Scale bar b, d, f, h, j = 5 µm.

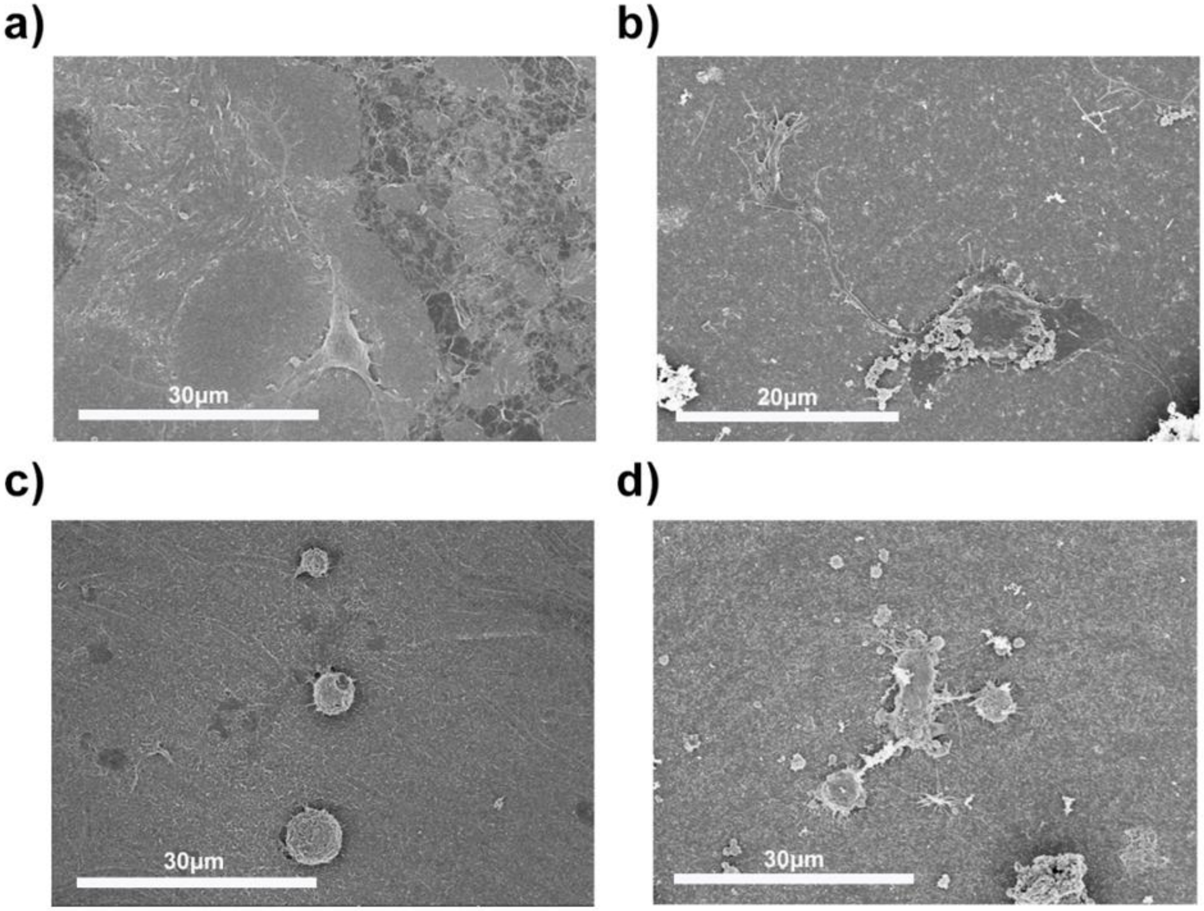

Supplementary Figure 47. SEM images of neurons cultured on DA-PA coatings after 48 hours. (a) **DA1-K**, (b) **DA1-VK**, (c) **DA1-VKVK**, and (d) **DA2-VK** coatings were prepared via the evaporation method on PDL glass coverslips.

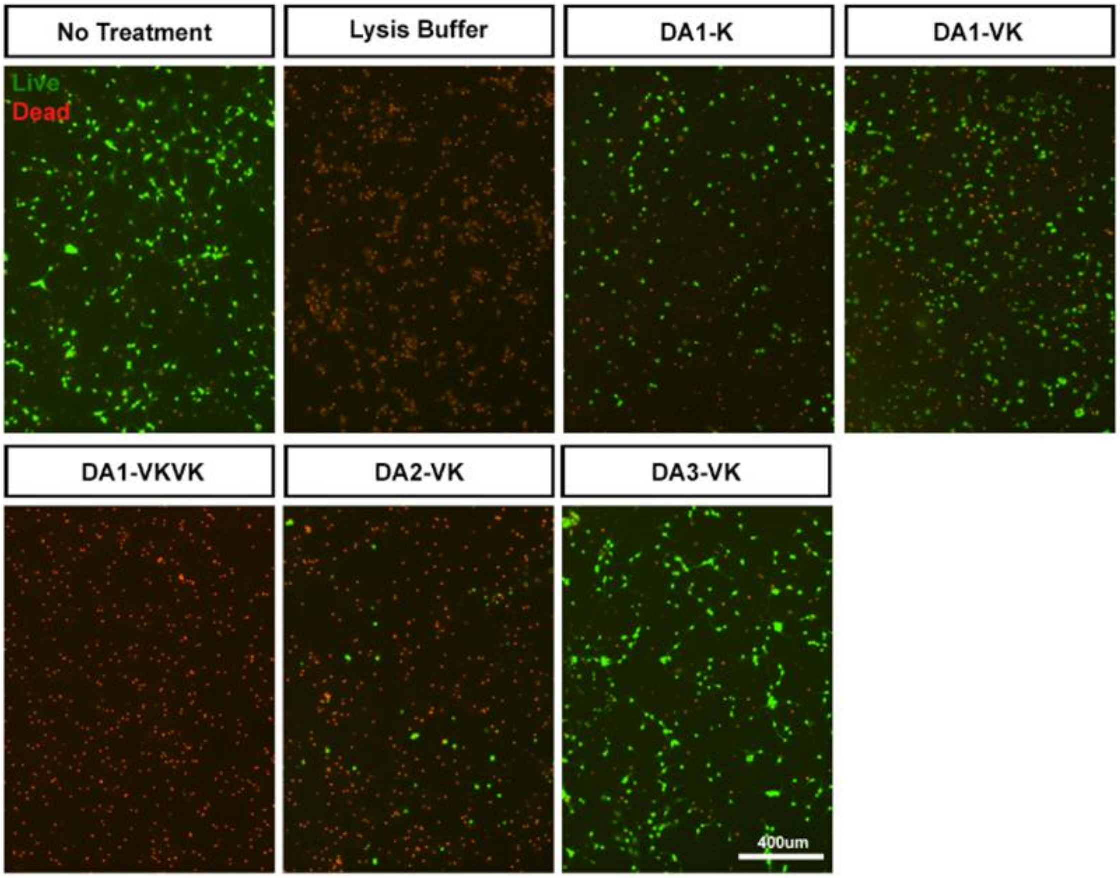

Supplementary Figure 48. Representative fluorescent images of neurons cultured on DA-PA coatings after one week. The samples were stained with acridine orange (green in live cells and red in dead cells) and propidium iodide (red).

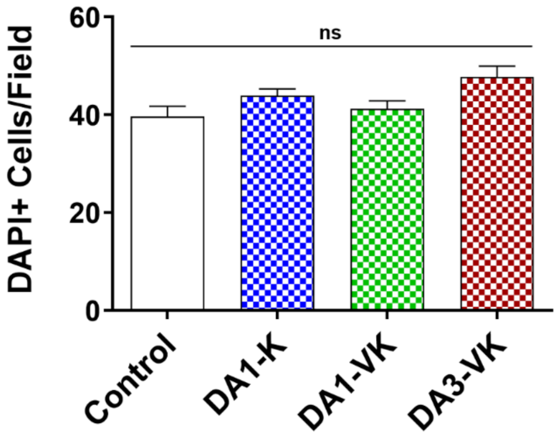

Supplementary Figure 49. Quantification of DAPI positive cells per field after a 48-hour culture. One-way ANOVA with a Tukey's multiple comparisons test ($\alpha = 0.05$) was performed: (*) $p < 0.05$, (**) $p < 0.01$, (***) $p < 0.001$, (****) $p < 0.0001$.

Neurons grown on **DA1-K** and **DA1-VK** for 48 hours had similar primary axon lengths to that of the control, while neurons grown on **DA3-VK** had significantly longer axons than all other experimental conditions (Figure 5d-e), with no significant differences in the number of neuronal cell bodies (Supplementary Figure 49).

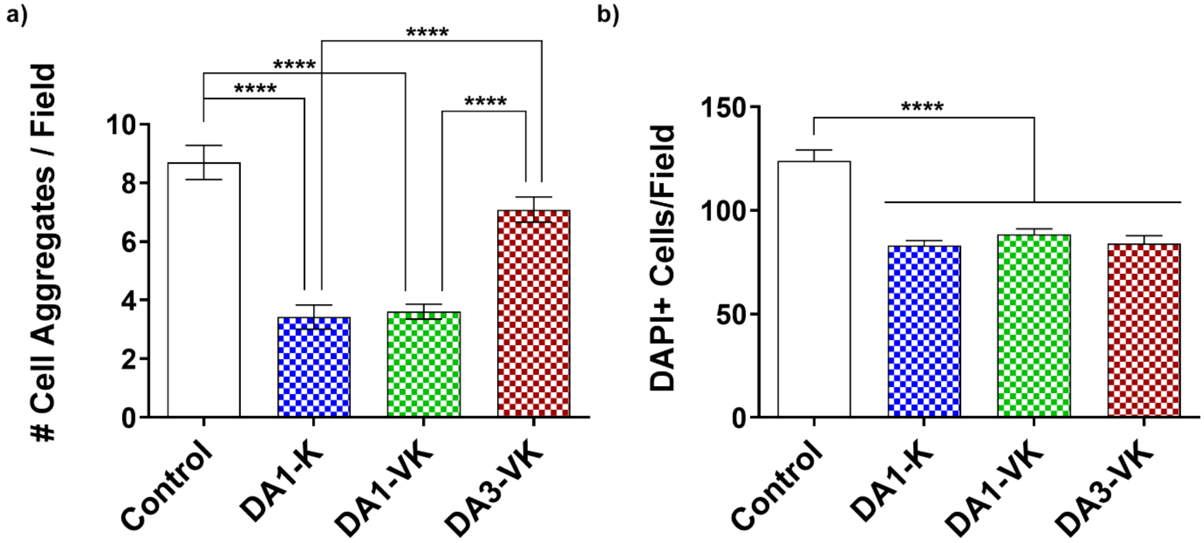

Supplementary Figure 50. Quantifications of cell aggregates and DAPI positive cells after a one-week culture. (a) Quantification of the number of cell aggregates per field. A cell aggregate was defined as three or more cells clustered together. (b) Quantification of DAPI positive cells per field. One-way ANOVA with a Tukey's multiple comparisons test ($\alpha = 0.05$) was performed: (*) $p < 0.05$, (**) $p < 0.01$, (***) $p < 0.001$, (****) $p < 0.0001$.

7.2 Whole-cell current-clamp experiments

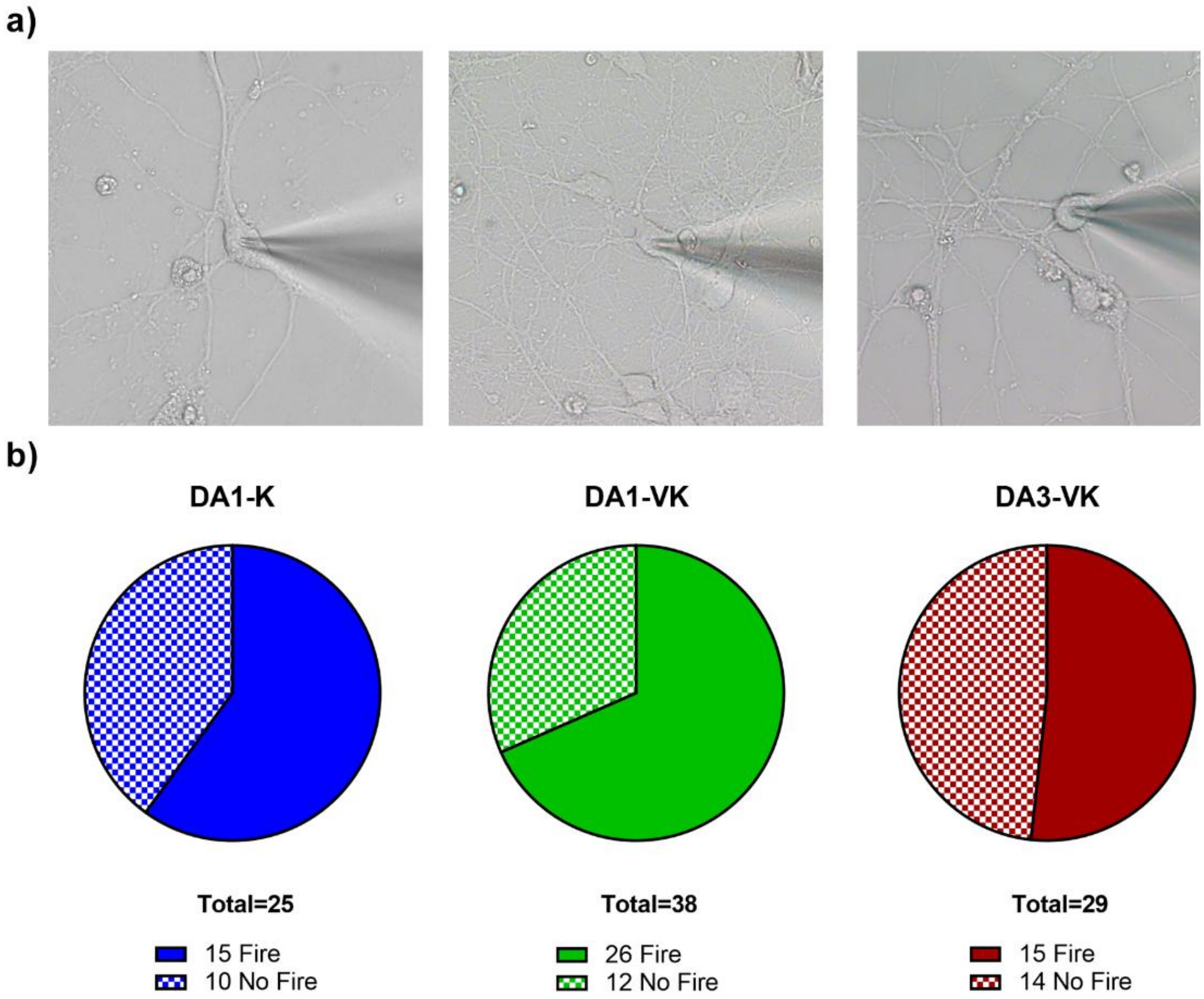

Supplementary Figure 51. Whole-cell current-clamp experiments. (a) Representative micrographs of a patched neuron grown on each condition. (b) Quantification of the total number of fire cells versus no fire cells in each condition. “Fire” cells were defined as neurons that were able to evoke at least one spike with an action potential peak (mV) reaching greater than -5 mV. “No fire” cells were defined as neurons that were unable to produce a spike.

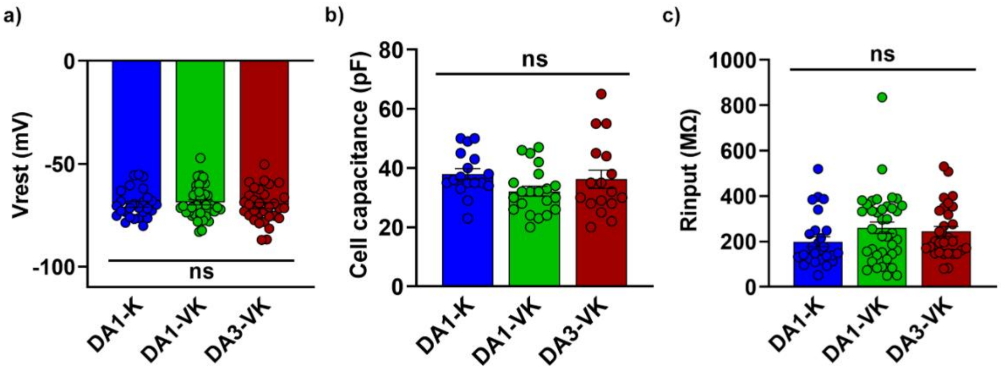

Supplementary Figure 52. Average values of (a) resting membrane potential voltage, (b) cell capacitance, and (c) input resistance of primary cortical neurons cultured for 12-14 days on DA-PA coatings. A poly-DL-lysine coating control was used for all experiments. For (a-c) one-way ANOVA with a Tukey's multiple comparisons test ($\alpha = 0.05$) was performed: (*) $p < 0.05$, (**) $p < 0.01$, (***) $p < 0.001$, (****) $p < 0.0001$.

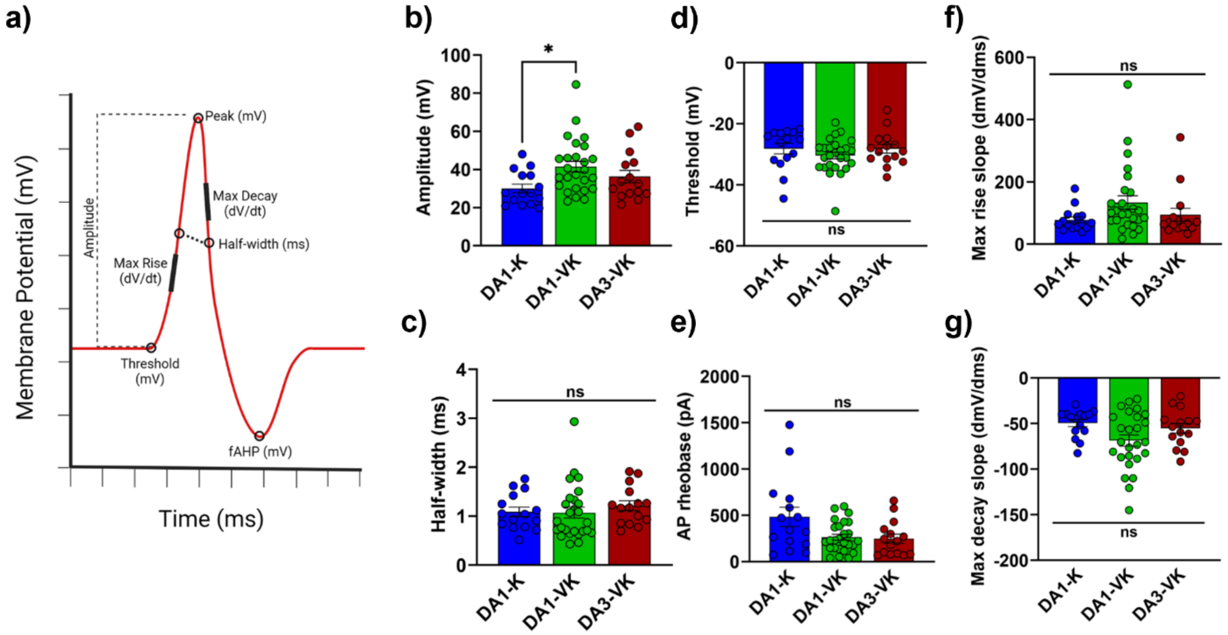

Supplementary Figure 53. Action potential properties of neurons cultured for 10-15 days on DA-PA coatings. (a) Representative action potential plot labelled with the action potential properties amplitude, peak, half-width, threshold, fAHP, maximum rise, and maximum decay. Quantification of the action potential properties (b) peak, (c) half-width, (d) threshold, (e) rheobase, (f) maximum rise, and (g) maximum decay. For (b-g) one-way ANOVA with a Tukey's multiple comparisons test ($\alpha = 0.05$) was performed: (*) $p < 0.05$, (**) $p < 0.01$, (***) $p < 0.001$, (****) $p < 0.0001$.

Reference:

[1] A. K. Blackburn, A. C. H. Sue, A. K. Shveyd, D. Cao, A. Tayi, A. Narayanan, B. S. Rolczynski, J. M. Szarko, O. A. Bozdemir, R. Wakabayashi, J. A. Lehrman, B. Kahr, L. X. Chen, M. S. Nassar, S. I. Stupp, J. F. Stoddart, *J Am Chem Soc* **2014**, *136*, 17224.

[2] J. A. Raskatov, J. P. Schneider, B. L. Nilsson, *Acc Chem Res* **2021**, *54*, 2488.

[3] C. Xiaogang, Y. Xi, Z. Desheng, Z. Liangying, *Ferroelectrics* **2001**, *259*, 55.

[4] Y. Yang, H. Sai, S. A. Egner, R. Qiu, L. C. Palmer, S. I. Stupp, *Nature* **2024**, *634*, 833.

[5] M. García-Iglesias, B. F. M. de Waal, A. V. Gorbunov, A. R. A. Palmans, M. Kemerink, E. W. Meijer, *J Am Chem Soc* **2016**, *138*, 6217.

[6] Furukawa, Nakajima, Takahashi, *IEEE Transactions on Dielectrics and Electrical Insulation* **2006**, *13*, 1120.

[7] A. S. Tayi, A. K. Shveyd, A. C. H. Sue, J. M. Szarko, B. S. Rolczynski, D. Cao, T. Jackson Kennedy, A. A. Sarjeant, C. L. Stern, W. F. Paxton, W. Wu, S. K. Dey, A. C. Fahrenbach, J. R. Guest, H. Mohseni, L. X. Chen, K. L. Wang, J. Fraser Stoddart, S. I. Stupp, *Nature* **2012**, *488*, 485.

[8] A. Narayanan, D. Cao, L. Frazer, A. S. Tayi, A. K. Blackburn, A. C. H. Sue, J. B. Ketterson, J. F. Stoddart, S. I. Stupp, *J Am Chem Soc* **2017**, *139*, 9186.

[9] R. A. Wiscons, N. R. Goud, J. T. Damron, A. J. Matzger, *Angewandte Chemie International Edition* **2018**, *57*, 9044.

[10] S. Barman, S. Bandyopadhyay, A. Ghosh, S. Das, T. Mondal, A. Datta, S. Ghosh, A. Datta, *Chemical Communications* **2022**, *58*, 10508.

[11] A. Mukherjee, S. Barman, A. Ghosh, A. Datta, A. Datta, S. Ghosh, *Angewandte Chemie International Edition* **2022**, *61*, DOI 10.1002/anie.202203817.

[12] Deepak, Z. Mallick, U. Sarkar, D. Mandal, R. K. Roy, *Chemistry of Materials* **2023**, *35*, 3316.

[13] H.-Y. Zhang, Y.-Y. Tang, Z.-X. Gu, P. Wang, X.-G. Chen, H.-P. Lv, P.-F. Li, Q. Jiang, N. Gu, S. Ren, R.-G. Xiong, *Science (1979)* **2024**, *383*, 1492.

[14] Y. Ai, P.-F. Li, M.-J. Yang, Y.-Q. Xu, M.-Z. Li, R.-G. Xiong, *Chem Sci* **2022**, *13*, 748.

[15] W. R. BUESSEM, L. E. CROSS, A. K. GOSWAMI, *Journal of the American Ceramic Society* **1966**, *49*, 33.
